# Mortality and Morbidity Among Individuals With Hypertension Receiving a Diuretic, ACE Inhibitor, or Calcium Channel Blocker: A Secondary Analysis of a Randomized Clinical Trial

**DOI:** 10.1001/jamanetworkopen.2023.44998

**Published:** 2023-12-04

**Authors:** Jose-Miguel Yamal, Journey Martinez, Mikala C. Osani, Xianglin L. Du, Lara M. Simpson, Barry R. Davis

**Affiliations:** 1 Coordinating Center for Clinical Trials, Department of Biostatistics and Data Science, School of Public Health, The University of Texas Health Science Center at Houston, Houston; 2 Department of Epidemiology, Human Genetics and Environmental Sciences, School of Public Health, The University of Texas Health Science Center at Houston, Houston; 3 Zimmer Biomet, Warsaw, Indiana

## Abstract

**Importance:**

The long-term relative risk of antihypertensive treatments with regard to mortality and morbidity is not well understood.

**Objective:**

To determine the long-term posttrial risk of primary and secondary outcomes among trial participants who were randomized to either a thiazide-type diuretic, calcium channel blocker (CCB), or angiotensin-converting enzyme (ACE) inhibitor with up to 23 years of follow-up.

**Design, Setting, and Participants:**

This prespecified secondary analysis of the Antihypertensive and Lipid-Lowering Treatment to Prevent Heart Attack Trial (ALLHAT), a multicenter randomized, double-blind, active-controlled clinical trial, followed up with participants aged 55 years or older with a diagnosis of hypertension and at least 1 other coronary heart disease risk factor for up to 23 years, from February 23, 1994, to December 31, 2017. Trial participants were linked with administrative databases for posttrial mortality (N = 32 804) and morbidity outcomes (n = 22 754). Statistical analysis was performed from January 2022 to October 2023.

**Interventions:**

Participants were randomly assigned to receive a thiazide-type diuretic (n = 15 002), a CCB (n = 8898), or an ACE inhibitor (n = 8904) for planned in-trial follow-up of approximately 4 to 8 years and posttrial passive follow-up for up to 23 years.

**Main Outcomes and Measures:**

The primary end point was mortality due to cardiovascular disease (CVD). Secondary outcomes included all-cause mortality, combined fatal and nonfatal (morbidity) CVD, and both mortality and morbidity for coronary heart disease, stroke, heart failure, end-stage renal disease, and cancer.

**Results:**

A total of 32 804 participants (mean [SD] age, 66.9 [7.7] years; 17 411 men [53.1%]; and 11 772 Black participants [35.9%]) were followed up for all-cause mortality and a subgroup of 22 754 participants (mean [SD] age, 68.7 [7.2] years; 12 772 women [56.1%]; and 8199 Black participants [36.0%]) were followed up for fatal or nonfatal CVD through 2017 (mean [SD] follow-up, 13.7 [6.7] years; maximum follow-up, 23.9 years). Cardiovascular disease mortality rates per 100 persons were 23.7, 21.6, and 23.8 in the diuretic, CCB, and ACE inhibitor groups, respectively, at 23 years after randomization (adjusted hazard ratio [AHR], 0.97 [95% CI, 0.89-1.05] for CCB vs diuretic; AHR, 1.06 [95% CI, 0.97-1.15] for ACE inhibitor vs diuretic). The long-term risks of most secondary outcomes were similar among the 3 groups. Compared with the diuretic group, the ACE inhibitor group had a 19% increased risk of stroke mortality (AHR, 1.19 [95% CI, 1.03-1.37]) and an 11% increased risk of combined fatal and nonfatal hospitalized stroke (AHR, 1.11 [95% CI, 1.03-1.20]).

**Conclusions and Relevance:**

In this secondary analysis of a randomized clinical trial in an adult population with hypertension and coronary heart disease risk factors, CVD mortality was similar between all 3 groups. ACE inhibitors increased the risk of stroke outcomes by 11% compared with diuretics, and this effect persisted well beyond the trial period.

**Trial Registration:**

ClinicalTrials.gov Identifier: NCT00000542

## Key Points

**Question:**

Is there a difference in the long-term risk of mortality and morbidity outcomes for adults with hypertension starting 1 of 3 antihypertensive treatments: thiazide-type diuretic, calcium channel blocker, or angiotensin-converting enzyme (ACE) inhibitor?

**Findings:**

In this prespecified secondary analysis of outcomes of 32 804 participants in a randomized clinical trial and posttrial up to 23 years later, there was no significant difference in mortality due to cardiovascular disease among the 3 antihypertensive treatment groups. However, there was an increased risk of stroke outcomes for ACE inhibitors compared with diuretics; after accounting for multiple comparisons, this increased risk was no longer significant.

**Meaning:**

Long-term follow-up supports the main findings that the diuretic group had similar cardiovascular outcomes and the ACE inhibitor group had higher stroke mortality risk.

## Teaser

This prespecified secondary analysis of a randomized clinical trial assesses the long-term posttrial risk of morbidity and mortality outcomes among participants who were randomized to a thiazide-type diuretic, calcium channel blocker, or ACE inhibitor with up to 23 years of follow-up.

## Introduction

The Antihypertensive and Lipid-Lowering Treatment to Prevent Heart Attack Trial (ALLHAT) was a multicenter, randomized, double-blind, active-controlled clinical trial comparing initial antihypertensive treatment with a calcium channel blocker (amlodipine), an angiotensin-converting enzyme inhibitor (lisinopril), or an α-blocker (doxazosin), all compared with a thiazide-type diuretic (chlorthalidone) for the composite outcome of fatal coronary heart disease (CHD) or nonfatal myocardial infarction (MI).^1^ The doxazosin group was terminated early due to a higher incidence of cardiovascular events. Participants in the other 3 groups were followed up for a mean (SD) of 4.9 (1.3) years during the trial, with 6-year primary outcome rates per 100 persons ranging from 11.3 to 11.5.^2^ No significant differences were detected among the 3 treatment groups for the primary outcome and all-cause mortality.^3^ Compared with the chlorthalidone group, the amlodipine group had a higher rate of heart failure (HF), and the lisinopril group had higher rates of cardiovascular disease (CVD), stroke, and HF.

A previous study reported mortality and morbidity outcomes of ALLHAT participants during 8 to 13 years after randomization using in-trial data plus posttrial data from administrative databases to assess the long-term effects of first-step antihypertensive treatment.^4^ During the posttrial period, no significant differences were detected among the 3 treatment groups for the primary outcome. Compared with chlorthalidone, the risk of HF was higher for the amlodipine group, and the risk of stroke mortality was higher for the lisinopril group (particularly for Black participants).

We linked the data to obtain an additional 11 years of passive administrative data since the last report, for a total of 19 to 24 years of follow-up after randomization. We aimed to determine whether the aforementioned legacy effects (ie, the observed in-trial differences) persisted after the trial and whether the increase in the incidence of events resulting from longer follow-up led to sufficient power to detect differences among the randomization groups.

## Methods

This prespecified secondary analysis of the ALLHAT, a multicenter, randomized, double-blind, active-controlled clinical trial, followed up with participants for up to 23 years, from February 23, 1994, to December 31, 2017. The methods and primary results of the ALLHAT and posttrial outcome results through 2006 have previously been published.^1,2,3,4,5^ Posttrial follow-up data were gathered from the National Death Index (NDI), Social Security Administration, and Center for Medicare & Medicaid Services (CMS) databases from the earliest available date through 2017. Participants in the trial provided written informed consent before study onset, and each of the 623 participating North American study centers obtained institutional review board approval for the trial. The institutional review board of The University of Texas Health Science Center at Houston likewise approved the long-term follow-up study (extension protocol and trial protocol in Supplement 1). This study followed the Consolidated Standards of Reporting Trials (CONSORT) reporting guideline.

### ALLHAT Participants and Double-Blind Protocol

Participants aged 55 years or older who received a diagnosis of hypertension and had a documented history of CVD, atherosclerotic CVD (ASCVD), and/or another risk factor for CHD were eligible for the ALLHAT.^2^ Participants were randomized to 4 treatment groups using a 1.7:1 ratio: chlorthalidone (n = 15 255), lisinopril (n = 9054), amlodipine (n = 9048), or doxazosin (n = 9061). At the beginning of the trial, participants engaged in a titration protocol of their randomized (step 1) medication group aimed at achieving a target blood pressure under 140/90 mm Hg. Participants were offered open-label step 2 and step 3 medications if their blood pressure could not be controlled at the maximum allowed daily dose of the step 1 drug. After completion of the initial titration phase, routine participant visits were scheduled for once every 3 months through the first year of the trial and once every 4 months from that point forward. Because of the limited in-trial follow-up due to early stopping, the doxazosin group was excluded from this extended posttrial follow-up (9061 participants excluded).

### Extended Follow-Up Outcome Definitions

#### Mortality Outcomes

Canadian participants (n = 553) were excluded from long-term follow-up because a lack of identifying information precluded the acquisition of comprehensive mortality records for the posttrial period, leaving 32 804 in the current study’s analytic sample (15 002, 8898, and 8904 in the chlorthalidone, amlodipine, and lisinopril groups, respectively). Deaths that occurred during the in-trial period were ascertained and classified by investigators and confirmed by medical records, including death certificates. If the cause of death was unknown to an investigator, a death certificate was obtained from the NDI. Posttrial mortality data, including date and cause of death, were obtained from the NDI Plus service, as was information for unknown causes of in-trial deaths. Details of the categorization of the causes of death and the handling of *International Statistical Classification of Diseases and Related Health Problems* coding were described in previous reports of posttrial mortality and morbidity and were demonstrated to be highly reliable.^4^ In brief, the causes of death were coded according to the *International Classification of Diseases, Ninth Revision* (*ICD-9*) for those occurring prior to 1999 and the *International Statistical Classification of Diseases and Related Health Problems, Tenth Revision* for those occurring in and after the year 1999 via the World Health Organization’s translation tool.^6^

The primary end point of this long-term follow-up study was mortality due to CVD, which was defined as death due to MI, stroke, or HF. Key secondary outcomes included all-cause mortality, mortality due to CHD, stroke mortality, HF mortality, and mortality due to end-stage renal disease (ESRD). Additional mortality outcomes included death due to the following causes: other CVD falling outside the predefined category, cancer of any type, and other non-CVD causes.

#### Morbidity Outcomes (Fatal and Nonfatal Events)

Nonfatal events were defined by hospitalization due to a prespecified cause. Except for ESRD, the availability of nonfatal outcome data was limited to participant medical records originating from US clinical centers unaffiliated with Veterans Affairs (VA) that were linked to valid Medicare or Social Security numbers. Therefore, participant medical records originating from VA sources (n = 5558) and non-Medicare participants (n = 4492) were excluded from long-term morbidity follow-up. Events that occurred during the in-trial period were ascertained and classified by investigators and confirmed by discharge summaries. Information on ESRD events (chronic dialysis or kidney transplant) that occurred during the in-trial period was gathered from the United States Renal Data System (USRDS) database through 2006. Data on posttrial morbidity outcomes were gathered from the CMS and USRDS databases. Classification of events identified through CMS data was accomplished using *ICD-9* codes originating from the source.

The following morbidity outcomes were prespecified as secondary end points: fatal or nonfatal CVD (death or hospitalization due to MI, stroke, or HF), fatal or nonfatal CHD (death or hospitalization due to CHD), fatal or nonfatal stroke (death or hospitalization due to stroke), fatal or nonfatal HF (death or hospitalization due to HF), and fatal or nonfatal ESRD (death or hospitalization due to ESRD). Cancer mortality and morbidity (death or hospitalization due to cancer of any type) were included as additional outcomes of interest.

### Covariates

Multivariable analyses were adjusted for relevant baseline demographic and clinical data, including dichotomized categories of 65 years of age or older, sex, self-identified Black race, self-identified Hispanic ethnicity, educational attainment, prior receipt of antihypertensive drug therapy, aspirin use, estimated glomerular filtration rate, high-density lipoprotein cholesterol level, past or current cigarette use, type 2 diabetes status, history of CHD, history of ASCVD, history of other ASCVD (history of angina pectoris; history of intermittent claudication, gangrene, or ischemic ulcers; history of transient ischemic attack; coronary, peripheral vascular, or carotid stenosis 50% or more documented by angiography or Doppler studies; ischemic heart disease documented by reversible or fixed ischemia on stress thalium or dipyridamole thalium, ST depression ≥1 mm for ≥1 minutes on exercise testing or Holter monitoring; reversible wall motion abnormality on stress echocardiogram; ankle-arm index <0.9; abdominal aortic aneurysm detected by ultrasonography, computed tomography scan, or X-ray; carotid or femoral bruits), history of MI or stroke, history of coronary artery bypass graft, major ST segment depression detected on electrocardiogram, left ventricular hypertrophy (ascertained from centrally coded baseline electrocardiograms using the Minnesota Code criteria of tall R‐wave in the presence of ST‐segment depression or T‐wave inversion), obesity (body mass index [BMI, calculated as weight in kilograms divided by height in meters squared] ≥30), systolic and diastolic blood pressure, and participation in the Lipid Lowering Trial. Because outcome data were evaluated from the time of randomization to the date of the last follow-up, it was not possible to make use of covariate measurements gathered during the in-trial period, extension period, or via external data sources (ie, CMS) after the date of randomization.

### Statistical Analysis

Statistical analysis was performed from January 2022 to October 2023. Differences in demographic and clinical characteristics among the randomized study groups at trial baseline were assessed using the χ^2^ test of independence for dichotomous and categorical variables and 1-way analysis of variance for continuous variables. The cumulative incidence of mortality and morbidity outcomes was calculated from the date of randomization (first randomization date for the mortality cohort: February 23, 1994; first randomization date for the morbidity cohort: March 10, 1994) to the date of last follow-up (December 31, 2017). For the evaluation of mortality outcomes, all participants whose date of death according to NDI records fell after the date of randomization were considered part of the population at risk. The population at risk for analyses of morbidity outcomes comprised members of the population at risk for the mortality cohort who had not experienced a nonfatal event of interest up to the date of randomization. The Kaplan-Meier method was used to calculate the 10-year and 23-year cumulative incidence rates of mortality and morbidity outcomes per 100 persons. Effect estimates of assigned treatments for the primary and secondary outcomes over the course of the full follow-up period were calculated using Cox proportional hazards regression and reported as hazard ratios (HRs) with 95% CIs. Multivariable Cox proportional hazards regression analyses adjusting for baseline covariates were also performed for the primary and secondary outcomes. The proportionality assumption was evaluated by the Schoenfeld residuals test and through visual inspection of the log-log Kaplan-Meier curves to ensure no intersection. To assess the heterogeneity of treatment effects by covariates (age, sex, race, type 2 diabetes status, and time period), interactions were tested using an unadjusted Cox proportional hazards regression model of each mortality and morbidity outcome separately for each covariate. As an alternative method for variable selection for the multivariable models, we applied the least absolute shrinkage and selection operator (LASSO) penalty to multivariable Cox proportional hazards regression models, using 10-fold cross-validation procedures to estimate the optimal lambda (tuning parameter). Selected variables were then used in supplementary multivariable Cox proportional hazards regression models. To test whether treatment effects differed during vs after the trial, we tested interactions between the randomization groups and a time-dependent indicator of whether the event occurred after the trial. To help guide the interpretation of the many analyses, we also applied the Holm multiple test procedure for the main effects separately for each randomization group pairwise comparison.^7^ All analyses were conducted using R, version 4.0.2 (R Project for Statistical Computing). All *P* values were from 2-sided tests and results were deemed statistically significant at *P* < .05.

## Results

### Patient Characteristics

**Figure 1.**
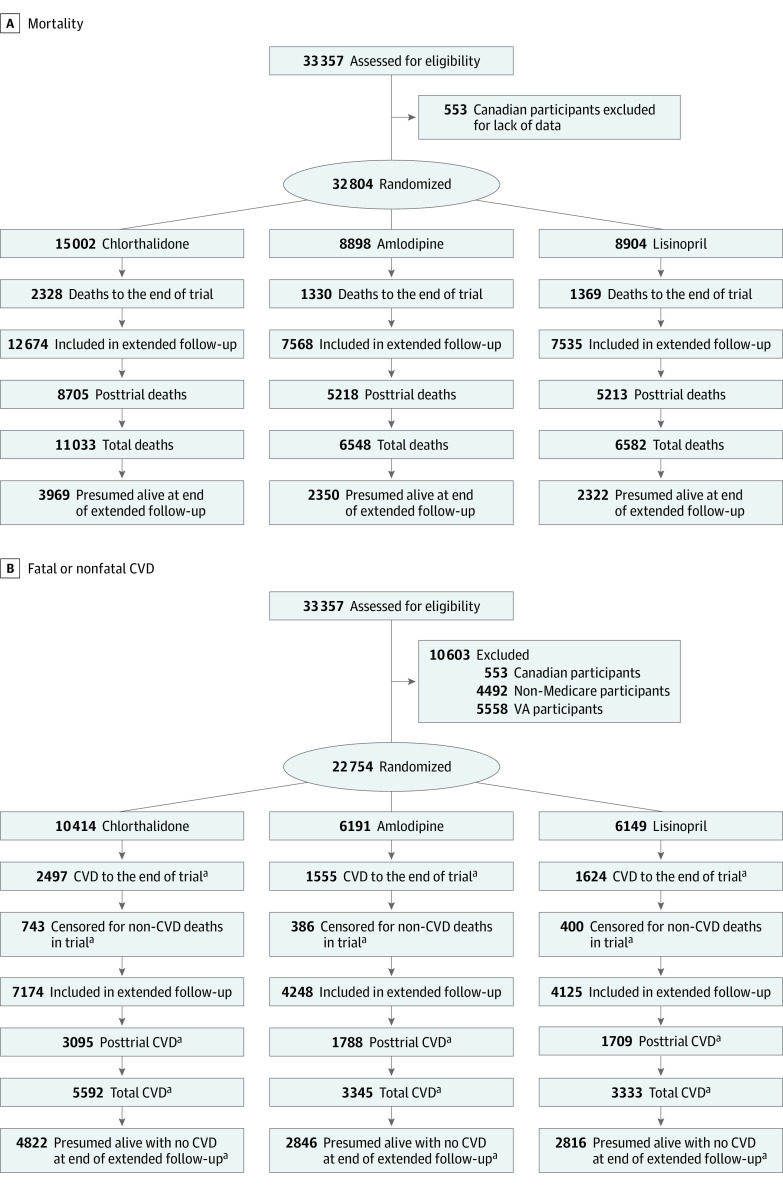
CONSORT Flow Diagram A, Mortality. B, Fatal or nonfatal cardiovascular disease (CVD). VA indicates Veterans Affairs. ^a^Fatal or nonfatal confirmed CVD included hospitalization or death due to myocardial infarction, stroke, or heart failure.

The mortality cohort comprised 32 804 participants (mean [SD] age at baseline, 66.9 [7.7] years; 17 411 men [53.1%] and 15 393 women [46.9%]; 11 772 Black participants [35.9%] and 6337 Hispanic participants [19.4%]) (Table 1; Figure 1A). Baseline factors were similarly distributed across randomization groups. The mean (SD) decrease in systolic blood pressure from baseline to the most recent reading date before the trial end date of March 31, 2002, was 9.9 (19.7) mm Hg, and the mean (SD) decrease in diastolic blood pressure was 6.7 (11.6) mm Hg (Table 1). The mean blood pressure decreased over time (until the latest reading prior to the end of the trial) for all 3 groups and most markedly in the chlorthalidone group for systolic blood pressure and the amlodipine group for diastolic blood pressure. The mean (SD) length of follow-up, including the posttrial period, was 13.7 (6.7) years, with a maximum follow-up of 23.9 years. The baseline characteristics for 22 754 participants (mean [SD] age, 68.7 [7.2] years; 9982 men [43.9%] and 12 772 women [56.1%]; 8199 Black participants [36.0%] and 4872 Hispanic participants [21.5%]) included in the analyses of extension morbidity outcomes (eg, fatal or nonfatal CVD) were well balanced among the randomization groups (eTable 1 in Supplement 2; Figure 1B).

**Table 1.** Baseline Characteristics for the Full Sample and by Randomized Group for Mortality Outcome Cohort

Characteristic	Participants, No. (%)^a^				
	Full sample (N = 32 804)	Chlorthalidone (n = 15 002)	Amlodipine (n = 8898)	Lisinopril (n = 8904)	*P* value^b^
Age, mean (SD), y	66.9 (7.7)	66.9 (7.7)	66.9 (7.7)	66.9 (7.7)	.93
Age group at trial baseline, y					
<65	13 899 (42.4)	6331 (42.2)	3774 (42.4)	3794 (42.6)	.82
≥65	18 905 (57.6)	8671 (57.8)	5124 (57.6)	5110 (57.4)	
Sex					
Male	17 411 (53.1)	7935 (52.9)	4684 (52.6)	4792 (53.8)	.24
Female	15 393 (46.9)	7067 (47.1)	4214 (47.4)	4112 (46.2)	
Race					
Black	11 772 (35.9)	5358 (35.7)	3209 (36.1)	3205 (36.0)	.84
Not Black	21 032 (64.1)	9644 (64.3)	5689 (63.9)	5699 (64.0)	
Hispanic or Latino ethnicity					
Hispanic	6337 (19.4)	2925 (19.6)	1675 (18.9)	1737 (19.6)	.38
Non-Hispanic	26 300 (80.6)	11 998 (80.4)	7179 (81.1)	7123 (80.4)	
Educational level, mean (SD), y	11.0 (4.0)	11.0 (4.0)	10.9 (4.0)	10.9 (4.1)	.87
Educational level at trial baseline					
High school or less	21 996 (72.0)	10 022 (71.6)	6005 (72.5)	5969 (72.2)	.31
More than high school	8550 (28.0)	3975 (28.4)	2277 (27.5)	2298 (27.8)	
Treatment with antihypertensive drugs prior to trial baseline					
Treated	29 633 (90.3)	13 544 (90.3)	8045 (90.4)	8044 (90.3)	.95
Untreated	3170 (9.7)	1457 (9.7)	853 (9.6)	860 (9.7)	
Aspirin use at trial baseline	11 693 (36.1)	5310 (35.9)	3197 (36.3)	3186 (36.3)	.70
Women taking estrogen at trial baseline	2683 (17.8)	1239 (17.9)	737 (17.8)	707 (17.6)	.92
eGFR at trial baseline, mean (SD), mL/min/1.73 m^2^	77.8 (19.8)	77.6 (19.7)	78.2 (19.7)	77.8 (19.9)	.07
eGFR <60 mL/min/1.73 m^2^ at trial baseline	5545 (17.7)	2565 (17.8)	1479 (17.4)	1501 (17.7)	.71
HDL cholesterol at trial baseline, mean (SD), mg/dL	46.8 (14.7)	46.8 (14.9)	47.2 (14.7)	46.6 (14.5)	.02
HDL <35 mg/dL at trial baseline	3812 (11.6)	1764 (11.8)	1004 (11.3)	1044 (11.7)	.51
Cigarette smoking at trial baseline					
Never smoker	12 463 (38.0)	5670 (37.8)	3404 (38.3)	3389 (38.1)	.97
Current smoker	7169 (21.9)	3284 (21.9)	1942 (21.8)	1943 (21.8)	
Former smoker	13 170 (40.1)	6048 (40.3)	3551 (39.9)	3571 (40.1)	
Diabetes classification at trial baseline					
Diabetes	13 010 (42.7)	5947 (42.7)	3572 (43.2)	3491 (42.2)	.38
Nondiabetes	17 468 (57.3)	7993 (57.3)	4689 (56.8)	4786 (57.8)	
History of CHD at trial baseline	8238 (25.3)	3862 (25.9)	2155 (24.4)	2221 (25.1)	.03
ASCVD at trial baseline	16 868 (51.4)	7753 (51.7)	4521 (50.8)	4594 (51.6)	.40
History of MI or stroke at trial baseline	7584 (23.1)	3514 (23.4)	2057 (23.1)	2013 (22.6)	.35
History of CABG at trial baseline	4224 (12.9)	1941 (12.9)	1084 (12.2)	1199 (13.5)	.04
Other ASCVD at trial baseline	7715 (23.5)	3514 (23.4)	2097 (23.6)	2104 (23.6)	.93
Major ST segment depression at trial baseline	3366 (10.4)	1552 (10.4)	891 (10.1)	923 (10.5)	.67
Left ventricular hypertrophy by Minnesota code at trial baseline	1480 (5.3)	672 (5.2)	395 (5.2)	413 (5.4)	.73
Lipid Lowering Trial participants	8036 (24.5)	3706 (24.7)	2194 (24.7)	2136 (24.0)	.43
BMI, mean (SD), at trial baseline	29.8 (6.2)	29.7 (6.2)	29.8 (6.3)	29.8 (6.2)	.56
Obesity (BMI ≥30) at trial baseline	13 797 (42.1)	6270 (41.8)	3787 (42.6)	3740 (42.0)	.51
Baseline BP, mm Hg					
Systolic BP, mean (SD)	145.5 (13.2)	145.5 (13.2)	145.5 (13.3)	145.6 (13.2)	.63
Diastolic BP, mean (SD)	83.6 (9.1)	83.6 (9.0)	83.5 (9.2)	83.7 (9.0)	.30
BP change from baseline to the latest BP reading prior to 3/31/2002, mm Hg					
Systolic BP, mean (SD)	−9.9 (19.7)	−10.5 (19.3)	−10.0 (19.2)	−8.8 (20.7)	<.001
Diastolic BP, mean (SD)	−6.7 (11.6)	−6.7 (11.5)	−7.1 (11.4)	−6.3 (12.0)	<.001

Abbreviations: ASCVD, atherosclerotic cardiovascular disease; BMI, body mass index (calculated as weight in kilograms divided by height in meters squared); BP, blood pressure; CABG, coronary artery bypass graft; CHD, coronary heart disease; eGFR, estimated glomerular filtration rate; HDL, high-density lipoprotein; MI, myocardial infarction.

SI conversion factor: To convert HDL cholesterol to millimoles per liter, multiply by 0.0259.

^a^ Percentages are calculated excluding missing values.

^b^ Significance level of the χ^2^ test of independence among randomized groups for binary and categorical variables or the 1-way analysis of variance among randomized groups for continuous variables. *P* < .05 is statistically significant.

### Primary and Secondary Outcomes

**Figure 2.**
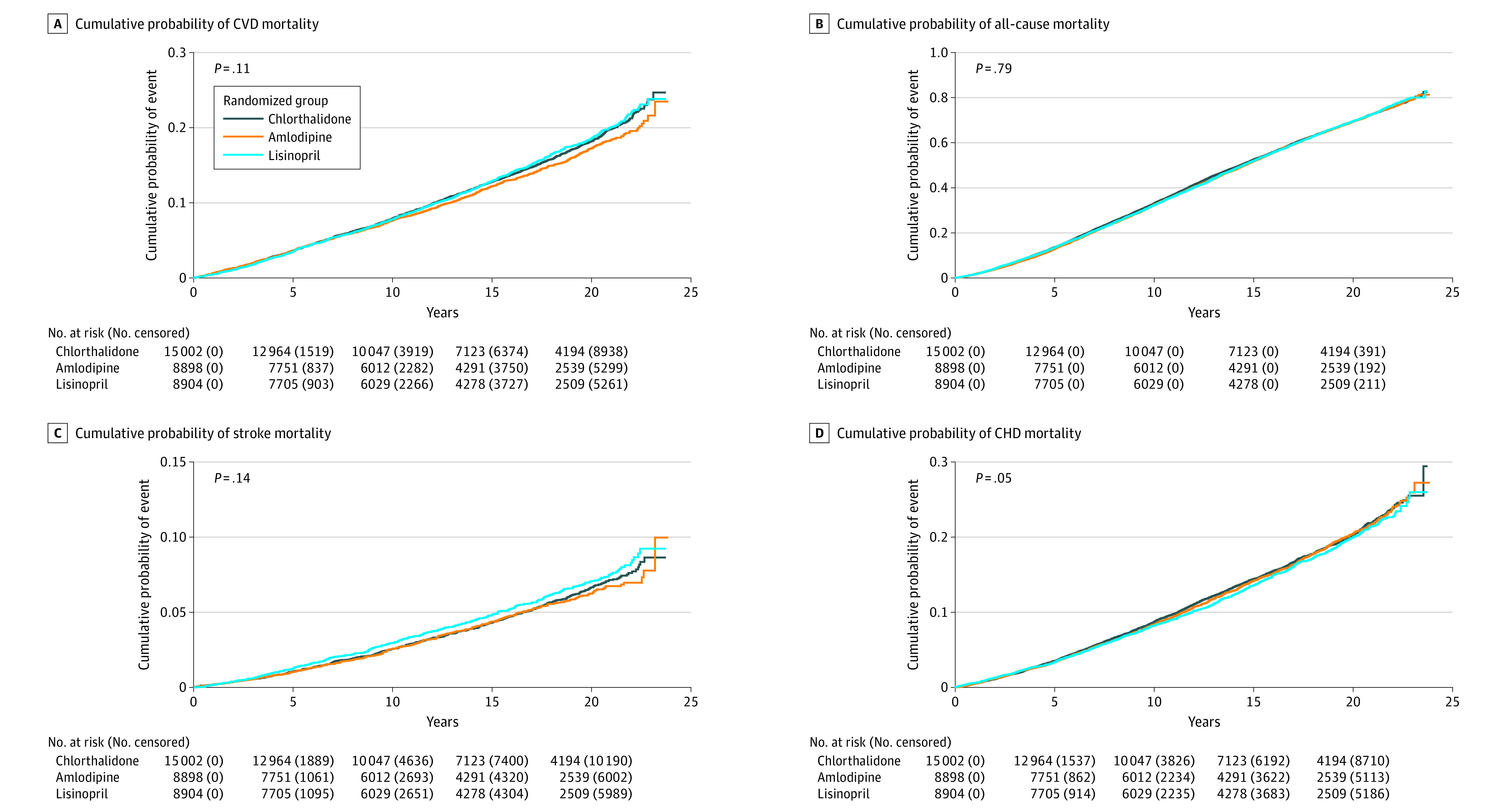
Kaplan-Meier Plots of Primary and Secondary Outcomes Through the Extended Follow-Up (by December 31, 2017) A, Cumulative probability of cardiovascular disease (CVD) mortality. B, Cumulative probability of all-cause mortality. C, Cumulative probability of stroke mortality. D, Cumulative probability of coronary heart disease (CHD) mortality.

Cumulative incidence rates of mortality and morbidity outcomes by treatment group are shown in Table 2 and Figure 2; eFigure 1A and 1B in Supplement 2. Cardiovascular disease mortality (the primary outcome) rates increased from 7.9 per 100 persons at 10 years after randomization to 23.7 per 100 persons at 23 years after randomization in the chlorthalidone group. Similarly, the rates increased from 7.7 per 100 persons at 10 years to 21.6 per 100 persons at 23 years in the amlodipine group and from 7.8 per 100 persons at 10 years to 23.8 per 100 persons at 23 years in the lisinopril group (Table 2), for an adjusted HR (AHR) of 0.97 (95% CI, 0.89-1.05) for amlodipine vs chlorthalidone and an AHR of 1.06 (95% CI, 0.97-1.15) for lisinopril vs chlorthalidone; Table 3). Rates of all-cause mortality increased from 33.1 to 79.2 per 100 persons in the chlorthalidone group, from 32.4 to 79.3 in the amlodipine group, and from 32.3 to 80.1 in the lisinopril group (Table 2). With a few exceptions, outcomes generally did not differ by randomization groups, as shown by the unadjusted HRs (Table 2) and after adjustment for baseline factors, including age, sex, race, Hispanic ethnicity, educational attainment, smoking status, treatment with antihypertensive drugs, obesity, ASCVD, other ASCVD, participation in the Lipid-Lowering Trial, diabetes, high-density lipoprotein cholesterol level less than 35 mg/dL (to convert to millimoles per liter, multiply by 0.0259), estimated glomerular filtration rate less than 60 mL/min/1.73 m^2^, ST segment depression, left ventricular hypertrophy, history of MI or stroke, history of CHD, aspirin use, and baseline blood pressures (Table 3). Lisinopril had a 19% increase in risk for kidney disease mortality compared with chlorthalidone over the 23 years of follow-up (HR, 1.19 [95% CI, 1.00-1.41]; Table 2), but this was not statistically significant after covariate adjustment (AHR, 1.18 [95% CI, 0.97-1.44]; Table 3). The Kaplan-Meier curves indicate an increased risk of kidney disease mortality after approximately 13 years (eFigure 1A in Supplement 2). Stroke mortality rates were 9.2 per 100 persons in the lisinopril group compared with 8.6 per 100 persons in the chlorthalidone group (HR, 1.10 [95% CI, 0.97-1.24]; AHR, 1.19 [95% CI, 1.03-1.37]), with sustained higher rates after 4 years postrandomization (Table 2, Table 3, and Figure 2C). Combined fatal and nonfatal hospitalized strokes also occurred more in the lisinopril group compared with the chlorthalidone group (HR, 1.06 [95% CI, 0.99-1.14]; AHR, 1.11 [95% CI, 1.03-1.20]). The full model regression coefficients for all factors are presented in eTables 2 to 4 in Supplement 2 for all-cause and CVD-related mortality outcomes, fatal or nonfatal hospitalized outcomes, and non-CVD mortality outcomes, respectively. In a post hoc comparison, lisinopril had a 19% higher risk of stroke mortality compared with amlodipine (AHR, 1.19 [95% CI, 1.01-1.40]; Table 3). After adjustment for multiple comparisons, no adjusted *P* values were less than .05 (not shown).

**Table 2.** Cumulative Incidence of Mortality and Morbidity Outcomes by Randomized Group With Follow-Up From February 23, 1994, to December 31, 2017^a^

Outcome	Chlorthalidone			Amlodipine vs chlorthalidone				Lisinopril vs chlorthalidone				Lisinopril vs amlodipine, unadjusted HR (95% CI)
	Events, No./total No.	10-y Rate (SE) per 100 persons	23-y Rate (SE) per 100 persons	Events, No./total No.	10-y Rate (SE) per 100 persons	23-y Rate (SE) per 100 persons	Unadjusted HR (95% CI)^b^	Events, No./ total No.	10-y Rate (SE) per 100 persons	23-y Rate (SE) per 100 persons	Unadjusted HR (95% CI)^b^	
**Mortality outcomes**												
All-cause mortality	11 025/15 002	33.1 (0.4)	79.2 (0.6)	6544/8898	32.4 (0.5)	79.3 (0.7)	0.99 (0.96-1.02)	6581/8904	32.3 (0.5)	80.1 (0.7)	1.00 (0.97-1.03)	1.01 (0.97-1.04)
CVD mortality^c^	1978/15 002	7.9 (0.2)	23.7 (0.9)	1107/8898	7.7 (0.3)	21.6 (1.1)	0.93 (0.87-1.01)	1197/8904	7.8 (0.3)	23.8 (1.1)	1.01 (0.94-1.09)	1.08 (1.00-1.18)
CHD mortality	2216/15 002	8.7 (0.2)	25.5 (0.8)	1310/8898	8.4 (0.3)	25.9 (1.1)	0.99 (0.92-1.06)	1272/8904	8.2 (0.3)	26.0 (1.3)	0.96 (0.90-1.03)	0.97 (0.92-1.05)
Stroke mortality	651/15 002	2.6 (0.1)	8.6 (0.6)	373/8898	2.6 (0.2)	7.8 (0.7)	0.96 (0.84-1.09)	427/8904	3.0 (0.2)	9.2 (0.7)	1.10 (0.97-1.24)	1.14 (1.00-1.32)
Heart failure mortality	440/15 002	1.5 (0.1)	7.0 (0.7)	255/8898	1.4 (0.1)	6.0 (0.6)	0.96 (0.83-1.12)	263/8904	1.4 (0.1)	6.5 (0.5)	1.00 (0.86-1.16)	1.03 (0.87-1.23)
Other CVD mortality	1343/15 002	4.5 (0.2)	17.8 (0.7)	809/8898	4.6 (0.2)	18.6 (1.0)	1.00 (0.92-1.09)	770/8904	3.9 (0.2)	18.0 (0.9)	0.96 (0.88-1.05)	0.95 (0.86-1.05)
Non-CVD mortality	6223/15 002	19.2 (0.3)	59.5 (0.9)	3708/8898	18.7 (0.4)	60.0 (1.1)	0.99 (0.95-1.03)	3752/8904	18.9 (0.4)	60.7 (1.1)	1.01 (0.97-1.05)	1.01 (0.97-1.06)
Cancer	2055/15 002	8.1 (0.2)	22.7 (0.7)	1228/8898	8.3 (0.3)	22.1 (0.7)	1.00 (0.93-1.07)	1294/8904	8.5 (0.3)	24.7 (0.9)	1.05 (0.98-1.13)	1.06 (0.98-1.14)
Kidney disease	326/15 002	1.1 (0.1)	5.7 (1.0)	194/8898	1.1 (0.1)	4.1 (0.3)	1.00 (0.83-1.19)	232/8904	1.1 (0.1)	5.9 (0.9)	1.19 (1.00-1.41)^d^	1.19 (0.99-1.44)
Other non-CVD disease	3581/15 002	10.3 (0.3)	42.5 (0.9)	2151/8898	9.7 (0.3)	44.4 (1.4)	1.00 (0.95-1.05)	2071/8904	9.5 (0.3)	42.6 (1.3)	0.96 (0.91-1.02)	1.15 (0.91-1.45)
Unknown causes of mortality	152/15 002	1.0 (0.1)	1.4 (0.1)	89/8898	1.0 (0.1)	1.2 (0.1)	0.98 (0.76-1.27)	97/8904	1.1 (0.1)	1.4 (0.1)	1.07 (0.83-1.38)	1.09 (0.82-1.46)
**Combined fatal and nonfatal hospitalized events**												
CVD^c^	5592/10 414	43.2 (0.5)	65.8 (0.8)	3342/6189^e^	43 (0.7)	66.1 (1)	1.00 (0.96-1.05)	3333/6149	43.4 (0.7)	66.6 (1.2)	1.02 (0.98-1.07)	1.02 (0.97-1.07)
CHD	2697/10 414	18.6 (0.4)	39.5 (0.8)	1603/6191	18.2 (0.5)	39.1 (1.3)	0.98 (0.92-1.04)	1561/6149	18.1 (0.5)	39.3 (1.6)	0.98 (0.92-1.04)	0.99 (0.93-1.06)
Heart failure	3962/10 414	29.8 (0.5)	51.7 (0.9)	2411/6191	30.3 (0.6)	52.4 (1.1)	1.02 (0.97-1.07)	2405/6149	31.1 (0.6)	53.5 (1.4)	1.04 (0.99-1.09)	1.02 (0.96-1.08)
Stroke	2251/10 414	18 (0.4)	30.3 (0.7)	1344/6189^e^	17.9 (0.5)	30.3 (1)	0.99 (0.93-1.06)	1410/6149	18.7 (0.5)	31.7 (0.9)	1.06 (0.99-1.14)	1.07 (0.99-1.15)
Cancer	2294/10 414	16.1 (0.4)	32.8 (0.8)	1374/6191	16.3 (0.5)	32.1 (0.9)	1.00 (0.93-1.07)	1431/6149	17.0 (0.5)	35.1 (1.1)	1.06 (0.99-1.13)	1.06 (0.99-1.14)
Kidney disease	625/10 414	4.3 (0.2)	12.0 (1.3)	384/6191	4.1 (0.3)	10.5 (0.6)	1.02 (0.90-1.16)	416/6149	4.3 (0.3)	12.7 (1.2)	1.12 (0.99-1.27)	1.10 (0.95-1.26)

Abbreviations: CHD, coronary heart disease; CVD, cardiovascular disease; HR, hazard ratio.

^a^ First randomization date in the mortality cohort.

^b^ Hazard ratios calculated with reference to chlorthalidone group.

^c^ Fatal or nonfatal confirmed CVD included hospitalization or death due to myocardial infarction, stroke, or heart failure.

^d^ Statistically significant at *P* < .05.

^e^ Excludes 2 participants with record of stroke hospitalization on the date of randomization.

**Table 3.** Adjusted Hazard Ratios for Mortality and Morbidity With Follow-Up From February 23, 1994, to December 31, 2017

Outcome	Adjusted hazard ratio (95% CI)^a^		
	Amlodipine vs chlorthalidone	Lisinopril vs chlorthalidone	Lisinopril vs amlodipine
**Mortality outcomes**			
All-cause mortality	1.00 (0.96-1.03)	1.01 (0.97-1.04)	1.01 (0.97-1.05)
CVD mortality^b^	0.97 (0.89-1.05)	1.06 (0.97-1.15)	1.10 (1.00-1.21)
CHD mortality	1.01 (0.94-1.10)	0.97 (0.90-1.06)	0.96 (0.88-1.05)
Stroke mortality	1.00 (0.86-1.16)	1.19 (1.03-1.37)^c^	1.19 (1.01-1.40)^c^
Heart failure mortality	1.01 (0.85-1.21)	1.03 (0.86-1.22)	1.01 (0.83-1.24)
Other CVD mortality	1.00 (0.91-1.11)	0.95 (0.85-1.05)	0.94 (0.84-1.06)
Non-CVD mortality	0.99 (0.95-1.04)^d^	1.01 (0.96-1.06)	1.02 (0.97-1.08)
Cancer	0.96 (0.88-1.04)	1.05 (0.97-1.14)	1.09 (1.00-1.20)
Kidney disease	1.00 (0.81-1.23)	1.18 (0.97-1.44)	1.18 (0.95-1.48)
Accident or suicide	0.86 (0.67-1.09)^d^	1.08 (0.86-1.35)^d^	1.25 (0.96-1.64)
Other non-CVD disease	1.02 (0.96-1.08)	0.97 (0.91-1.03)	0.95 (0.89-1.02)
Unknown causes of mortality	1.01 (0.74-1.38)	0.97 (0.70-1.33)	0.95 (0.67-1.36)^d^
**Combined fatal and nonfatal hospitalized events**			
CVD^b^	1.01 (0.96-1.06)	1.04 (0.99-1.09)^d^	1.02 (0.97-1.08)
CHD	0.99 (0.92-1.06)	0.98 (0.91-1.05)	0.99 (0.91-1.07)
Heart failure	1.02 (0.97-1.09)^d^	1.03 (0.97-1.09)^d^	1.00 (0.94-1.07)
Stroke	1.04 (0.96-1.12)	1.11 (1.03-1.20)^c^	1.07 (0.98-1.17)
Cancer	0.99 (0.92-1.07)	1.02 (0.94-1.10)	1.03 (0.94-1.12)
Kidney disease	1.05 (0.90-1.22)	1.12 (0.97-1.30)	1.07 (0.91-1.26)

Abbreviations: CHD, coronary heart disease; CVD, cardiovascular disease.

^a^ Adjusted for age 65 years or older at trial baseline, sex, race, Hispanic ethnicity, educational attainment, smoking status, treatment with antihypertensive drugs prior to trial baseline, obesity at trial baseline, atherosclerotic CVD at trial baseline, other atherosclerotic CVD at trial baseline, participation in Lipid Lowering Trial, diabetes status at trial baseline, high-density lipoprotein cholesterol less than 35 mg/dL (to convert to millimoles per liter, multiply by 0.0259) at trial baseline, estimated glomerular filtration rate less than 60 mL/min/1.73 m^2^ at trial baseline, ST segment depression at trial baseline, left ventricular hypertrophy (ascertained from centrally coded baseline electrocardiograms using the Minnesota Code criteria of tall R‐wave in the presence of ST‐segment depression or T‐wave inversion) at trial baseline, history of myocardial infarction or stroke at trial baseline, history of CHD at trial baseline, aspirin use at trial baseline, baseline systolic blood pressure per 10 mm Hg, and baseline diastolic blood pressure per 10 mm Hg.

^b^ Fatal or nonfatal confirmed CVD included hospitalization or death due to myocardial infarction, stroke, or heart failure.

^c^ Statistically significant at *P* < .05.

^d^ Significant interactions with in-trial period were found. Amlodipine vs chlorthalidone: non-CVD mortality, *P* = .04; accident or suicide mortality, *P* = .02; combined hospitalizations for congestive heart failure, *P* ≤ .001. Lisinopril vs chlorthalidone: accident and suicide mortality, *P* = .005; combined hospitalizations for congestive heart failure, *P* = .004; combined hospitalizations for CVD, *P* = .009. Lisinopril vs amlodipine: unknown cause mortality, *P* = .04.

Alhough the candidate covariates for some models differed when LASSO-penalized regression methods were applied as a variable selection procedure, the resulting AHRs were nearly identical to those that were produced from the full multivariable model, and the 95% CIs were of comparable width (eTables 5-7 in Supplement 2).

Subgroup analyses by age, sex, race and ethnicity, and type 2 diabetes status demonstrated consistent results with overall analyses for most outcomes, including CVD mortality (eFigures 2A-C in Supplement 2). Among those with baseline diabetes, participants in the lisinopril group had a lower risk of all-cause mortality (HR, 0.96 [95% CI, 0.92-1.01]; *P* = .02 for interaction) and CHD mortality (HR, 0.88 [95% CI, 0.79-0.97; *P* = .03 for interaction) than those in the chlorthalidone group (eFigure 2A in Supplement 2). Older individuals (≥65 years) had a lower risk of stroke mortality in the amlodipine group compared with the chlorthalidone group (HR, 0.88 [95% CI, 0.75-1.02]; *P* = .03 for interaction). Among men, those in the lisinopril group had a higher risk of fatal or nonfatal CVD than those in the chlorthalidone group (HR, 1.07 [95% CI, 1.01-1.14]; *P* = .04 for interaction; eFigure 2B in Supplement 2), and those in the amlodipine group had a higher risk of fatal or nonfatal stroke (HR, 1.09 [95% CI, 0.98-1.20]; *P* = .03 for interaction; eFigure 2B in Supplement 2) and fatal or nonfatal HF (HR, 1.08 [95% CI, 1.00-1.17]; *P* = .046 for interaction; eFigure 2C in Supplement 2). Among participants who were not Black, those in the lisinopril group had a lower risk of fatal and nonfatal CHD (HR, 0.93 [95% CI, 0.86-1.01]; *P* = .04 for interaction).

## Discussion

The present study uses several national databases to obtain long-term clinical outcomes in a subset of ALLHAT participants. Posttrial follow-up of ALLHAT participants was accomplished by linking trial data with Medicare data from the CMS from 2002 to 2006.^4^ Several studies used these 4-year posttrial data to examine the risk of mortality, HF, stroke, ESRD, and hip and pelvic fractures.^4,8,9,10,11^ However, the 4-year posttrial period was short. It is still unknown whether any of the original legacy effects persist or diminish or whether new effects will arise over the next 10 years or beyond.

The use of administrative data has gained increased popularity in clinical trials and has been recognized by the US Food and Drug Administration as important to advance the development of therapeutic products.^12^ Ascertainment of these long-term posttrial outcomes in a large study such as ALLHAT using traditional methods would have been very expensive and resource intensive. Although limited, the use of administrative data has made this type of analysis feasible.

In contrast to the in-trial and 8-year to 13-year analyses, we now observed that the lisinopril group had an increased risk of kidney disease mortality that emerged after approximately 13 years after randomization, but this effect was attenuated after adjustment for baseline variables. During the in-trial period, lisinopril was associated with a higher risk of stroke, which persisted during our follow-up periods, as seen in the Figure 2 separation of the lisinopril curve around year 4 and then the parallel curves throughout the follow-up. Therefore, this observed difference is mainly attributed to the in-trial difference, although the interaction with the in-trial period was not significant. A previous study hypothesized that the in-trial to posttrial difference (up to 12 years) for CVD (in-trial HR, 1.05 [95% CI, 0.98-1.14]; posttrial HR, 0.92 [95% CI, 0.85-1.00]) suggests a delayed effect for lisinopril.^4^ However, the current extended posttrial results suggest that there is no significant difference between lisinopril and chlorthalidone for the CVD outcome.

### Limitations

This analysis has some important limitations similar to the last follow-up report. First, participants may have stopped the randomization drug once they were unblinded. The unblinding also may have lead to biases. Second, we were not able to conduct a passive outcome ascertainment for all ALLHAT participants due to data availability (Canadian participants were omitted from mortality and combined mortality and morbidity analyses, and VA participants and those without a Medicare number were omitted from the combined mortality or morbidity analyses), resulting in a reduced sample size. Therefore, the individuals in the morbidity and mortality cohorts may not be representative of the general population. However, there were no noted significant differences in the measured baseline demographic distribution for this cohort compared with the full study. Third, posttrial antihypertensive medication use is unknown between study completion (2002) and 2006, and crossover or regression to more similar medications is possible, which could dilute the original treatment distinctions among the 3 randomization groups. A previous study examined posttrial antihypertensive medications based on Medicare Part D–linked data from 2007 and found significant mixing of antihypertensive classes.^13^ For example, in 2007, 44%, 40%, and 41% of those in the chlorthalidone, lisinopril, and amlodipine groups, respectively, were prescribed a thiazide or a thiazide-type diuretic. Fourth, blood pressure and laboratory data were not obtained after the trial. Fifth, after adjustment for multiple comparisons, none of the analyses were statistically significant. We, therefore, interpreted the magnitude of the HRs for consistency with in-trial analysis and previous posttrial analysis with particular caution. However, with 11 years of additional passive follow-up (2006-2017), the results for lisinopril vs chlorthalidone for stroke and stroke mortality are almost the same.

## Conclusions

In this prespecified secondary analysis of a randomized clinical trial using Medicare billing data to obtain follow-up for up to 23 years after baseline, we found similar results as those found during the ALLHAT. Angiotensin-converting enzyme inhibitors were associated with an increased risk of stroke outcomes (11% increased risk of combined fatal and nonfatal hospitalized stroke) compared with diuretics, and this effect persisted well beyond the trial period.

## Notes

Supplement 1.
Trial Protocols

Supplement 2. **eTable 1.** Baseline Characteristics for the Full Sample and by Randomized Group for Morbidity Study at the Time of Randomization **eTable 2.** Adjusted Hazard Ratios for All-Cause and CVD Mortality With Follow-Up From 2/23/1994 to 12/31/2017 **eTable 3.** Adjusted Hazard Ratios for Morbidity With Follow-Up From 3/10/1994 to 12/31/2017 **eTable 4.** Adjusted Hazard Ratios for Non-CVD Mortality With Follow-Up From 2/23/1994 to 12/31/2017 **eTable 5.** Hazard Ratios Adjusted by Variables Selected Through Penalized Regression for All-Cause and CVD Mortality With Follow-Up From 2/23/1994 to 12/31/2017 **eTable 6.** Hazard Ratios Adjusted by Variables Selected Through Penalized Regression for Morbidity With Follow-Up From 3/10/1994 to 12/31/2017 **eTable 7.** Hazard Ratios Adjusted by Variables Selected Through Penalized Regression for Non-CVD Mortality With Follow-Up From 2/23/1994 to 12/31/2017 **eFigure 1A.** Kaplan-Meier Plots of Primary and Secondary Outcomes Through the Extended Follow-Up (by 12/31/2017) **eFigure 1B.** Kaplan-Meier Plots of Primary and Secondary Outcomes Through the Extended Follow-Up (by 12/31/2017) **eFigure 2A.** Unadjusted Hazard Ratios (95% CI) and Forest Plots of Overall and Subgroup Analyses of Mortality and Morbidity Outcomes Through the Extended Follow-Up (by 12/31/2017) – Amlodipine vs. Chlorthalidone and Lisinopril vs. Chlorthalidone **eFigure 2B.** Unadjusted Hazard Ratios (95% CI) and Forest Plots of Overall and Subgroup Analyses of Mortality and Morbidity Outcomes Through the Extended Follow-Up (by 12/31/2017) – Amlodipine vs. Chlorthalidone and Lisinopril vs. Chlorthalidone **eFigure 2C.** Unadjusted Hazard Ratios (95% CI) and Forest Plots of Overall and Subgroup Analyses of Mortality and Morbidity Outcomes Through the Extended Follow-Up (by 12/31/2017) – Amlodipine vs. Chlorthalidone and Lisinopril vs. Chlorthalidone

Supplement 3.
Data Sharing Statement
