# Supplementary material for: Mortality and Morbidity Among Individuals With Hypertension Receiving a Diuretic, ACE Inhibitor, or Calcium Channel Blocker: A Secondary Analysis of a Randomized Clinical Trial
Source: JAMA Netw Open. 2023 Dec 4;6(12):e2344998. doi: 10.1001/jamanetworkopen.2023.44998 (PMC10696481; doi:10.1001/jamanetworkopen.2023.44998)
Supplement: Supplement 1. — Trial Protocols [file jamanetwopen-e2344998-s001.pdf]

Post-Trial Follow-up of ALLHAT Participants Planned Through 2006

Active follow-up of ALLHAT participants ended on March 31, 2002 (February 15, 2000 for doxazosin participants), for an average follow-up of 4.9 years (3.2 years for doxazosin). To answer additional scientific questions, a post-trial follow-up of participants through 2006 is planned to obtain data on post-trial morbidity and mortality. The extended follow-up will be carried out passively using national databases available through the National Death Index (NDI), Social Security Administration (SSA), Centers for Medicare and Medicaid Services (CMS), Department of Veterans Affairs (DVA), and United States Renal Data System (USRDS). When completed, endpoint data will be available for between 9 and 10 years of total follow-up.

The overall research objective of this extended follow-up is to compare long-term effects of antihypertensive treatment with a thiazide-type diuretic, a CCB, an ACE-inhibitor, or an α-receptor blocker when each drug is used as initial treatment with step-up drugs added as needed and, for the lipid component, to assess long-term effects of pravastatin compared with usual care. To meet these objectives, we will evaluate whether new differences emerge for outcomes that were not statistically different, especially CVD and total mortality, and whether the differences observed during the trial continue.

The ALLHAT extension protocol was developed by the CTC and the NHLBI Project Office and approved by the ALLHAT Steering Committee.

The original ALLHAT Protocol may be found at http://allhat.sph.uth.tmc.edu/Forms/protocol.pdf.

EXTENSION PROTOCOL
The Antihypertensive and Lipid-Lowering Treatment to Prevent Heart Attack Trial (ALLHAT)
August 31, 2007

I. Introduction

The purpose of this document is to provide details on data to be collected during the post-trial phase of ALLHAT, to pose hypotheses the data can be expected to address, and to describe how the data will be analyzed. The previously published ALLHAT data presented here are from the September 30, 2002, locked ALLHAT database; the projections, however, are based on observed results from the Limited Access Data Set (LADS) of July 31, 2006, which is updated with deaths up to the end of the trial identified through June 27, 2006.

II. Background

a. ALLHAT Antihypertensive Trial (AHT)

The AHT was a multi-center randomized double-blind, active-controlled clinical trial, designed to determine whether the occurrence of fatal coronary heart disease (CHD) or nonfatal myocardial infarction (MI), the AHT primary endpoint, is lower for high-risk hypertensive patients (*n*=42,418) treated initially with a calcium channel blocker (CCB), an ACE inhibitor, or an alpha-blocker compared with those initially treated with a thiazide-type diuretic. Pre-specified secondary endpoints are listed in Tables 1 and 2.[1]

Eligible participants were men and women $\geq$ 55 years of age with untreated SBP $\geq$ 140 mm Hg, and/or DBP $\geq$ 90 mm Hg, or who took antihypertensive medication (<3 drugs) with blood pressure $\leq$160/100 mm Hg ($\leq$180/110 mm for untreated and treated for less than 2 months), and who had at least one additional risk factor for CHD.[1] Of 42,418 randomized participants, 47% were women, 81% were aged 60 or older (mean 67), and 36% had type 2 diabetes. By race/ethnicity, 35% were black and 19% were Hispanic.[2]

a.1 Antihypertensive Trial: Chlorthalidone, Amlodipine, Lisinopril

Participants in the chlorthalidone (*n*=15,255), amlodipine (*n*=9,048), and lisinopril (*n*=9,054) treatment groups were followed for a mean of 4.9 years. The primary outcome occurred in 2,956 participants, with no difference between treatment arms. Compared with chlorthalidone (6-year rate, 11.5%), the relative risks (RRs) were 0.98 (95% CI, 0.90-1.07) for amlodipine (6-year rate, 11.3%) and 0.99 (95% CI, 0.91-1.08) for lisinopril (6-year rate, 11.4%). Likewise, all-cause mortality did not differ between the randomized treatment groups. For amlodipine vs chlorthalidone, secondary outcomes were similar except for a higher 6-year rate of heart failure (HF) with amlodipine (10.2% vs 7.7%; RR, 1.38; 95% CI, 1.25-1.52). For lisinopril vs chlorthalidone, lisinopril had higher 6-year rates of combined cardiovascular disease (CCVD; 33.3% vs 30.9%; RR, 1.10; 95% CI, 1.05-1.16); stroke (6.3% vs 5.6%; RR, 1.15; 95% CI, 1.02-1.30); and HF (8.7% vs 7.7%; RR, 1.19; 95% CI, 1.07-1.31).[3] For stroke, there was a statistically significant race by treatment interaction; chlorthalidone was superior to lisinopril in preventing incident and recurrent stroke only in Blacks (RR; 95% CI=1.40; 1.17- 1.68 for Blacks and 1.00; 0.85- 1.17 for non-Blacks). When cardiovascular and renal outcomes were analyzed by e-GFR

(estimated glomerular filtration rate) strata the results were consistent with those for the entire cohort. *Post-hoc* analyses showed modestly higher incidence of diabetes in the diuretic group compared to both amlodipine and lisinopril groups. While there is no conclusive or consistent evidence that the diuretic-associated increase in incident diabetes leads to an increase in clinical events concerns have been raised about long term cardiovascular effects of the excess incident diabetes. In contrast, no excess morbidity or mortality was noted in the diuretic arm in ALLHAT participants with diabetes at baseline or diagnosed during the trial. A long-term follow-up of the SHEP trial cohort (14.3 years) confirmed these findings with respect to mortality in those with diabetes at baseline.[4]

a.2 Antihypertensive Trial: Chlorthalidone, Doxazosin

Participants in the doxazosin treatment group (*n*=9061), and the comparable chlorthalidone group (*n*=15,255), were followed for a mean of 3.2 years as the doxazosin arm was terminated early (February 15, 2000) due to a nearly two-fold higher risk of incident HF and futility of reaching a statistically significant difference in the primary endpoint.[5, 6] The primary outcome occurred in 1317 (818 + 499) participants, with no difference between treatment arms. Compared with chlorthalidone (4-year rate, 7.76%), the relative risk (RR) was 1.03 (95% CI, 0.92-1.15) for doxazosin (4-year rate, 7.91%). Likewise, all-cause mortality did not differ between the groups (RR, 1.03; 95% CI, 0.94-1.13). Significantly higher risks of stroke and combined CVD, however, occurred in doxazosin treated participants relative to those assigned to chlorthalidone: for stroke, RR = 1.26 (95% CI: 1.10-1.46); for combined CVD, RR = 1.20 (95% CI: 1.13-1.27). The RR (95% CI) for HF, a pre-specified component of CCVD, was 1.80 (1.61- 2.02).[6] The clinical outcomes were similar for those with and without glucose disorders at baseline despite lower glucose levels in the doxazosin arm during follow-up.[7]

The ALLHAT antihypertensive trial results led to a conclusion that inexpensive thiazide-type diuretics are superior to an ACE-inhibitor, a calcium channel blocker and an alpha-receptor blocker in preventing one or more major forms of CVD. They should be preferred first-step antihypertensive therapy.

b. ALLHAT Lipid-Lowering Trial (LLT)

The LLT was a randomized open-label clinical trial in a subset of ALLHAT participants (n= 10,335) assigned to either pravastatin (PV) or usual care (UC). Eligibility criteria included 1) prior enrollment in ALLHAT; 2) fasting LDL-C of 120 to 189 mg/dL for those with no known CHD, or 100 to 129 mg/dL for those with known CHD, and 3) fasting triglyceride (TG) levels < 350 mg/dL. Mean follow-up was 4.8 years. All-cause mortality (primary endpoint) did not differ between the two randomized treatment groups (relative risk [RR], 0.99; 95% confidence interval [CI], 0.89-1.11; *P*=.88), with 6-year mortality rates of 14.9% for the PV group vs 15.3% for UC. CHD event rates (fatal CHD or non-fatal MI) were not significantly different between the groups (HR, 0.91; 95% CI, 0.79-1.04; *P*=.16), with 6-year CHD event rates of 9.3% for the PV group and 10.4% for UC. CVD or any other cause-specific mortality did not differ significantly between the two randomized treatment groups. Similarly, no difference was observed in the incidence of cancer. However, statistically significant race by outcome interaction (black versus non-black) was noted for stroke and CHD events with a greater benefit for CHD in Blacks and for stroke in non-Blacks.[8] These unexpected outcomes by race are currently being explored in a manuscript in progress.

These results led to a conclusion that pravastatin did not reduce either all-cause mortality or CHD significantly when compared with usual care in older participants with well-controlled hypertension and moderately elevated LDL-C. The results may be due to the modest differential in total cholesterol (9.6%) and LDL-C (16.7%) between PV and UC compared with prior statin trials supporting their use for cardiovascular disease prevention.

III. Research Questions

An important expectation at the outset of ALLHAT was that better metabolic profiles of the newer antihypertensive drugs might provide an advantage over the diuretic. The ALLHAT results confirmed the superior metabolic profiles of the newer drugs, but did not find them superior to the diuretics in preventing clinical outcomes. Moreover, the diuretic was superior to each of the comparator drugs in preventing one or more cardiovascular outcomes. For the LLT, the expectation was that total mortality and CHD events, as well as cause-specific mortality will be lower in PV compared to UC. After an average 4.8 years of follow-up, no difference was observed in either total mortality or CHD event rates possibly due to a lesser difference in the total and LDL-cholesterol levels than in other trials, and there was no difference in the incidence of cancer.

For the AHT, the overall objective of this passive post-trial mortality and morbidity surveillance using administrative data bases is to assess long-term effects of antihypertensive treatment with a thiazide-type diuretic, a CCB, an ACE-inhibitor, or an α-receptor blocker when each drug is used as initial treatment with step-up drugs added as needed. To meet this objective, we will evaluate 1) whether statistically significant differences in clinical outcomes observed during the trial persisted or disappeared; 2) whether differences emerge for outcomes that were not statistically different at the trial's end; and 3) whether observed differences in non-fatal outcomes result in long-term differences in CVD and/or total mortality. For the LLT, as no statistically significant differences were observed during the trial, the post-trial surveillance can only consider the questions related to persistence of the lack of differences between treatment groups or a potential emergence of differences.

In engaging the research questions, it has to be recognized that given the passive nature of this follow-up we will not have any information about medications used by ALLHAT participants after the trial close-out. In addition, information about post-trial hospitalizations will be available only for the Medicare and VA populations. These issues will be addressed through appropriate analytical approaches.

In the AHT, we expect that following the trial's closeout, the majority of patients continued to be treated with antihypertensive drugs. While a number of them, especially those whose BP was controlled during the trial, likely continued on medications they had been on during the trial, others might have their treatment regimens changed for various reasons and these would generally reflect treatment patterns in the community. As a result, there would be a lesser difference in treatment regimens across ALLHAT original randomized treatment groups. While we would expect a large proportion of participants, especially in the chlorthalidone group, to be treated with a thiazide or a thiazide-type diuretic alone or in combination with other drugs, it will likely not be chlorthalidone due to prevailing practice patterns and a general perception of these diuretics having a class effect. We also expect BP control to be slightly lesser than during the trial, but not differ across treatment groups.

Two large clinical trials, the Hypertension Detection and Follow-up Program (HDFP) and the Multiple Risk Factors' Intervention Trial (MRFIT), published results of a long-term mortality follow-up of patients randomized to treatment of hypertension and related them to BP treatment and control.[9, 10] HDFP Post Trial Follow-up (PTS) actively followed all participants (*n*=10,940) for 8.2 years. The trial had a 5-year fixed duration of follow-up, but a proportion of the participants was treated as long as 6.7 years. Data collected were 1) total and cause-specific mortality and 2) post-trial treatment status and BP control in Stepped Care (SC) and Referred Care (RC). The post-trial use of antihypertensive medications declined in SC and increased in RC participants so that by the end of the post-trial period there was little difference in percentages of SC and RC participants taking medication. Control of DBP was slightly better for SC than for RC participants. The total mortality advantage found at 6.7 years persisted and increased throughout the post-trial period of follow-up despite discontinuation of the formal SC program. In MRFIT, a mortality follow-up (total and cause-specific) of 12,866 men was conducted 16 years after randomization to Special Intervention (SI) or Usual Care (UC) to address long-term effects of CV risk factor intervention on CHD, CVD, and total mortality. Information on levels of risk factors was obtained on participants at 4 of 22 clinical centers at ~ 3 years after the trial ended. Virtually no difference existed between the study groups in the proportion of men using antihypertensive medication while some difference in DBP persisted. In both trials these may have reflected acquisition of better adherence behavior in the SI (MRFIT) and SC (HDFP) groups during in-trial care at study sites. Similar to HDFP, differences between SI and UC in mortality rates from MI, CHD, and all causes were greater during the post-trial compared to in-trial period.

Since ALLHAT compared 4 active treatment regimens with the same BP goal across treatment groups, and because the achieved BP levels were similar, the follow-up outcomes will in part reflect long-term effects of medications with the small observed BP level differences being a part of an overall "drug package." This is different from HDFP and MRFIT where in-trial outcome differences were primarily driven by differential BP control, though in MRFIT differences within the class of thiazide/thiazide-like diuretics were engaged.[9, 10]

In the LLT, we expect that following the trial's closeout most patients in both arms were treated with statins, maybe with the exception of those without CHD and LDL<130 mg/dL. It is likely that in the PV group, the majority continued on pravastatin while in the UC, the majority was treated with other statins or life-style approaches as during the trial investigators were asked to avoid using pravastatin in participants assigned to PV. Recently, two clinical trials of lipid-lowering with statins in patients with CHD--the Long-term Intervention with Pravastatin in Ischemic Disease (LIPID) and the Scandinavian Simvastatin Survival Study (4S)--have reported results of their extended follow-ups.[11, 12] In LIPID, following an early termination of the placebo-controlled trial (*n*=9104) due to efficacy, all patients without contraindications were offered an open-label pravastatin irrespective of their original therapy and were invited to participate in a long-term follow-up. Mortality and morbidity as well as total and LDL-cholesterol levels were assessed over an additional 2 years of follow-up. During the follow-up period (*n*=7680), patients originally assigned pravastatin had almost identical cholesterol concentrations to those assigned placebo, but lower risk of death from all-causes, CHD death, and CHD death or non-fatal MI. When the entire period was considered, the risk of stroke was also lower. In 4S (*n*=4444), patients were also followed for an additional 2 years (median overall follow-up 7.4 years). All patients were advised to take simvastatin, but no medication was provided. Cholesterol levels and use of lipid-lowering drugs were ascertained through questionnaires sent to all participants. Slightly, though statistically significantly, fewer patients were taking cholesterol-lowering drugs (mainly simvastatin) in

the placebo compared to the simvastatin group 3.4 years after study closure. Cholesterol levels were also slightly lower in the original statin group. At the end of the extended follow-up, there were fewer deaths in the simvastatin group, mainly due to fewer CHD deaths. Non-cardiovascular mortality did not differ.

Participants in the Lipid Research Clinics Coronary Primary Prevention Trial, a randomized, cholesterol-lowering trial comparing cholestyramine (*n*=1907) with placebo (*n*=1899) treatment in asymptomatic men conducted between 1973 and 1983 (7.4 years, mean follow-up), were followed up annually (by mail, phone or in-person) from 1985 until 1989 (a total of additional 6 years).[13] Post-trial treatment was not provided. Similar increasing proportions of cholestyramine and placebo participants reported use of cholesterol-lowering drugs post-trial (25-55%). The difference in the mean lipid levels disappeared after the double-blind was broken and drug intervention ceased despite the persistence of slightly higher use of drugs (mainly resins) in the original cholestyramine group. Notably, use of diet was slightly higher in the original placebo group. While the cumulative mortality in the placebo group tended to exceed that in the intervention group throughout the period of follow-up, it has never reached statistical significance. The statistically significant in-trial reduction in CHD incidence rates in the cholestyramine group did not continue after cessation of the study intervention. Cancer incidence rates were similar, except for GI tumors which were non-significantly increased in the original intervention arm.

The following specific research questions will be considered (also see Table 3):

a.1 AHT: Will the observed advantages of diuretics over comparator drugs continue?

i. Amlodipine versus Chlorthalidone

In the amlodipine comparison with chlorthalidone, there was no difference in cardiovascular mortality (6-year rate 8.5% in the amlodipine arm versus 8.0% in the chlorthalidone arm; *p*=0.76). Non-cardiovascular mortality was lower in the amlodipine arm (6-year rate 8.0% versus 8.9% in the chlorthalidone; *p*=0.04) with unintentional injury/homicide/suicide being statistically significant (*p*=0.005). As a result, the all-cause mortality slightly, though not significantly, favored amlodipine (6-year rate 17.3% versus 16.8%; *p*=0.20).

Across CVD endpoints (fatal or non-fatal), the only statistically significant difference between chlorthalidone and amlodipine was a 38% highly statistically significant increased risk of incident HF in the amlodipine group (p<.001). The combined CVD occurrence non-significantly favored chlorthalidone (RR=1.04; *p*=0.12); the RR for PAD (hospitalized or treated) was 0.87, *p*=0.06 and for stroke 0.93, p=.28. Consequently, the most important research question in the extended follow-up is whether the substantial increase in the incidence of HF in the amlodipine arm will continue (see **a.5**) and whether it will result in an increased CVD morbidity and/or mortality.

ii. Lisinopril versus Chlorthalidone

In the lisinopril comparison with chlorthalidone, there were no differences in either cardiovascular or non-cardiovascular mortality (6-year rate 8.5% and 8.6% in the lisinopril arm compared with 8.0% and 8.9% in the chlorthalidone arm, *p*=0.39 and 0.57 for CVD and non-CVD mortality, respectively). Consequently, there was no difference in total mortality (6-year rates 17.3% in the chlorthalidone arm versus 17.2% in the lisinopril arm, *p*=0.90).

While the primary outcome did not differ between the treatment groups (RR=0.99, *p*=0.81), the CCVD rates were higher in the lisinopril compared to the chlorthalidone arm (RR=1.10; *p*<0.001). The RR; *p*-value for stroke were 1.15; *p*=0.02, for HF (hospitalized or treated without hospitalization) 1.19; *p*=0.001 and for angina (hospitalized or treated without hospitalization) 1.11; *p*=0.01. Notably, there was a statistically significant outcome by race interaction (black versus non-black) for stroke and for CCVD, but not for other outcomes. Of note is also an age by outcome interaction for a combined CHD outcome with increased risk in those 65+. The interaction was statistically significant only for the pre-specified dichotomous age subgroups (<65 versus 65+) but not when age was modeled as a continuous variable. The increased risk of combined CHD may become more pronounced as the ALLHAT population ages and may even affect the CVD outcome.

The extended follow-up will have a potential to determine whether these differences persist and result in a difference in CVD mortality, especially in Blacks and those 65+.

iii. Doxazosin versus chlorthalidone

The doxazosin arm was terminated early due to an observed 25% increased risk of CCVD (with risk of HF nearly doubled) and futility of observing a statistically significant difference in the primary endpoint by the scheduled end of the trial. The final report was based on data through February 15, 2000. No difference in the all-cause mortality was observed. However, CVD mortality favored chlorthalidone (RR; 95% CI=1.15; 1.01-1.32) and included a 39% increased stroke mortality (*p*=0.03). In addition, relative to chlorthalidone, the doxazosin group experienced an 80% greater risk of HF (fatal, hospitalized, or treated, *p*<0.001), a 26% greater risk of stroke (*p*<0.001), and a 13% greater risk of angina (with or without hospitalization, *p*=0.01). Thus, the most important research question for the extended follow-up will be whether these higher risks will contribute to further divergence in the CVD mortality curves for the two groups.

a.2 AHT: Will the observed lack of differences across randomized comparisons with respect to CHD (AHT primary endpoint) persist?

The better metabolic profiles of the newer drugs did not translate into a superior CHD outcome in any of the comparisons. Notably, BP control was superior in the chlorthalidone group, especially in comparison with the doxazosin and the lisinopril arms. The observed lack of advantage of the amlodipine-, lisinopril-, and doxazosin-based treatments over the chlorthalidone-based strategy for CHD (CHD mortality and non-fatal MI), including in those with diabetes at baseline, will be explored to especially

address concerns about potential long-term effects of the diuretic-induced dysglycemia and resulting incident diabetes on progression of atherosclerosis (see a.4). The stroke outcome will also be evaluated.

a.3 AHT: Will the observed lack of difference across randomized comparisons with respect to total mortality persist?

During ALLHAT active follow-up, non-CVD mortality constituted about half of the total mortality. The ratio of non-CVD to total mortality may even become larger as the ALLHAT population (55+ at the time of enrollment) ages. Since ALLHAT interventions did not aim to improve non-CVD morbidity, we would not expect any changes in non-CVD mortality except for a small shift resulting from competing risks of death if CVD deaths are prevented. During the trial, no statistically significant differences in total mortality were observed.

Notably, during the active phase of the trial, the amlodipine group had lower incidence of non-CVD mortality primarily due to a lower incidence of accidental deaths (unintentional injury/homicide/suicide). This finding was most likely due to chance as it was not seen in previous studies and it has no known biological explanation. In addition, prior to ALLHAT there were concerns about gastrointestinal bleeding and cancer being potential adverse effects of amlodipine – findings not confirmed in ALLHAT. In contrast, cancer mortality trended lower in the amlodipine group (6-year rate per 100 = 4.3 versus 3.8; p=0.31).

Another surprising outcome, though not statistically significant, was a trend towards higher incidence of hospitalizations for gastrointestinal bleeding (RR=1.11; p=0.07) in the lisinopril group.

These findings will be explored in the context of total mortality and its components. Cancer mortality is of special interest as it is known to take longer to emerge.

a.4 AHT: Will diuretic-induced dysglycemia result in adverse clinical outcomes?

In light of the well documented lower incidence of diabetes, especially in patients treated with ACE-inhibitors and alpha-blockers compared to those treated with thiazide diuretics, an important research question for the ALLHAT extension is whether the difference in the CCVD outcome will be maintained over the longer period of follow-up, especially in participants with a history of diabetes at baseline or in those who developed diabetes during active follow-up, and whether the observed difference in the CCVD outcome will result in a difference in CVD mortality over the longer period of observation. CCVD components of special interest are the primary endpoint (no difference), HF (difference in favor of chlorthalidone), and stroke (difference in favor of chlorthalidone in Blacks).

Data related to the long-term effects of incident diabetes are crucial to the interpretation of ALLHAT's findings. Such hypotheses for total mortality and CVD mortality were addressed in SHEP by the SHEP extension study.[4] In addition to the SHEP-extension-

type hypotheses, we also intend to analyze CVD events. In the ALLHAT diabetes incidence paper for the A/L/C comparison, which related CVD outcomes to incident diabetes occurring during the trial in a time-dependent manner, participants assigned to chlorthalidone had a higher rate of incident diabetes than did participants assigned to amlodipine or to lisinopril.[14] There was no significant association of fasting glucose change at 2 years with subsequent CHD, stroke, CVD, total mortality, or ESRD. There was no significant association of incident diabetes at 2 years with clinical outcomes, except for CHD (RR=1.64, *p*=0.006), but RR was lower and non-significant in the chlorthalidone group. The extended follow-up will allow for a total 6-7 years of observation following incident diabetes compared to about 3 years in the published data.

For the doxazosin comparison with chlorthalidone, only diabetes status at baseline was analyzed (known diabetes and newly diagnosed). While there was no difference in the primary endpoint or total mortality, HF rates were higher in those treated with doxazosin in both those with known and newly diagnosed diabetes at baseline (RR; 95% CI= 1.85; 1.56- 2.19 and 1.63; 1.05- 2.55, respectively).

a.5 AHT: Will the differences in heart failure (HF) incidence observed during the trial continue over a longer period of follow-up? Will the sustained advantage for C vs A continue? Will the similar HF rates for C and L after the first year of follow-up become an advantage for L? Will the mortality and hospitalization burden associated with HF remain similar, increase or decrease?

ALLHAT extended follow-up will also provide an opportunity to assess the incidence of hospitalized HF over a longer period of time as well as the case-fatality and mortality associated with incident HF events. The average duration of follow-up post-hospitalized HF will be 6-7 years. Mortality associated with HF events with preserved and impaired ejection fraction will also be evaluated. Assessment of a burden of hospitalizations following incident HF events may also be included.

a.6 AHT: Will the observed lack of superiority for kidney outcomes of lisinopril over chlorthalidone persist and/or will the observed higher estimated glomerular filtration rate (e-GFR) in the amlodipine group translate into lower incidence of ESRD (initiation of chronic renal dialysis or kidney transplant)? Will the advantage of chlorthalidone on CVD outcomes across the renal function spectrum at baseline persist?

Given the morbidity, mortality and costs associated with progressive kidney disease, it is important to establish the long term renal outcomes associated with antihypertensive drug therapy. Further, given the increase in CVD risk associated with impaired renal function, long-term CVD outcomes by renal functions at baseline are also of interest.

i. Amlodipine versus Chlorthalidone

At baseline, eGFR was similar between ALLHAT participants assigned to amlodipine and chlorthalidone.[15] However, at the end of the study participants assigned to

amlodipine had a significantly higher eGFR than those on chlorthalidone.[15] This may suggest a "renoprotective" effect of amlodipine compared to chlorthalidone. However, the incidence of end stage renal disease did not differ between the groups. If the elevated GFR reflects preservation of renal function, it would be expected that over a longer follow-up period, the amlodipine arm will have a lower ESRD rate than chlorthalidone. We hypothesize that the high GFR with amlodipine is a manifestation of the effect of amlodipine on renal microcirculation; amlodipine causes afferent vasodilatation that results in increased intraglomerular pressure.[16] While this results in a transient elevation in GFR, long term elevation in intraglomerular pressure may promote glomerulosclerosis and actually accelerate decline in renal function. This physiologic concept is supported by data from ALLHAT which shows an early increase in GFR in participants assigned to amlodipine. Similarly, in the AASK study which compared amlodipine with ramipril, GFR was initially higher in participants on amlodipine, but clinical renal events were lower with ramipril.[17]

Patients with impaired renal functions are at increased risk for CVD events. Across eGFR strata ($\geq$90; 60- 89; and <60 mL/min per 1.73 m^2) at baseline, outcomes across treatment groups were similar to those for the entire cohort (see **a.1**). An important question is whether the observed superiority of chlorthalidone over amlodipine will persist over a longer period of follow-up.

ii. Lisinopril versus Chlorthalidone

The lack of added benefit in the lisinopril arm with respect to ESRD was rather surprising, and it will be important to determine whether a difference will emerge over a longer period of follow-up. CVD outcomes across the spectrum of renal functions, which were similar to those in the entire cohort, will also be of interest.

iii. Doxazosin versus chlorthalidone

ESRD and CVD outcomes will be assessed for the overall cohort and by e-GFR strata.

a.7 AHT: Will the observed lack of statistically significant difference in hospitalizations for gastrointestinal bleeding continue?

Hospitalization for gastrointestinal bleeding was one of two major safety outcomes which were prespecified in the ALLHAT original protocol; the other one was angioedema. Prior to ALLHAT results, a body of literature based on observational studies and secondary CHD prevention trials of short-acting CCBs has suggested that CCBs, especially DHP-CCBs, may increase the risk of gastrointestinal bleeding, cancer, and all-cause mortality. The results of ALLHAT did not support these findings. Unexpectedly, the mortality from all-causes was significantly lower in the amlodipine group, primarily due to lower incidence of accidental deaths. Notably, the incidence of hospitalizations for gastrointestinal bleeding did not differ between the amlodipine and the diuretic arms (RR, 95% CI=0.92; 0.82- 1.03). However, for L versus C this RR, 95% CI was 1.11,

0.99- 1.24. The extended follow-up will allow addressing these interesting findings with respect to an important safety concern over a longer period of observation.

a.8 LLT: Will the small observed difference in cholesterol levels favoring pravastatin result in improved CHD and/or stroke outcomes without affecting cancer or total mortality?

The ALLHAT lipid component was an open-label comparison of pravastatin (PV) versus usual care (UC). The drop-in rate in the control group was nearly 20% by year 4 and nearly 30% by year 6, resulting in a lower that anticipated difference in the achieved lipid levels between the randomized treatment groups (9% for total cholesterol and 14% for LDL cholesterol). As the investigators were asked to avoid treating the UC group participants with pravastatin, it is likely that other statins were predominantly used in the UC group (information not collected during the trial).

At the end of the ALLHAT active follow-up, there was no difference between treatment groups in the all-cause mortality (6-year rate 19.9 in PV and 15.3 in UC; RR=0.99, p=0.88) – the LLT primary endpoint. The RRs for the CVD and non-CVD mortality were 0.99 and 1.01, respectively. The fatal CHD and non-fatal MI endpoint was slightly, but not significantly, lower in the PV group (RR; 95% CI = 0.91; 0.79- 1.04) and similarly was fatal and non-fatal stroke (RR; 95% CI= 0.91; 0.75- 1.09). For both the stroke and the CHD endpoints, there was a significant heterogeneity by race. For the CHD event, RR (95% CI) was 0.73 (0.58-0.92) for Blacks and 1.02 (0.86-1.21) for non-blacks (interaction p = 0.03). For stroke, the RR (95% CI) were 1.12 (0.86-1.48) in Blacks versus 0.74 (0.57-0.96) in non-Blacks (interaction p = 0.03).

Cancer mortality was non-significantly higher in the PV group compared to UC (1.11; 0.89-1.30); the RR (95% CI) for a composite of fatal and non-fatal cancer was 1.03 (0.89-1.19).

ALLHAT enrolled older individuals with no history of CHD, including those with LDL<130 mg/dL. While evidence on long-term safety of statins has accumulated over the past decade, there is still some uncertainty about long-term outcomes, CHD benefit versus non-CHD risk (especially cancer) in this relatively low-CHD risk subgroup. At the end of the active follow-up, we reported for this post-hoc subgroup a non-significantly higher risk of total mortality (1.18; 0.90-1.56) and also a non-significantly lower risk of a composite endpoint of CHD deaths and non-fatal MI (0.73; 0.49- 1.07). Cancer incidence and cancer mortality risks in this subgroup were not significantly different in PV compared to UC (0.92; 0.64-1.33 and 1.69; 0.95-3.01, respectively). Consequently, both total and CVD mortality over extended period of time will be of great interest as well as CHD mortality and morbidity and cancer mortality and morbidity.

Given the paucity of information on renal outcomes, long-term effects on ESRD should be evaluated. Also of potential interest are the effects on HF incidence and re-hospitalizations – in light of the recent approval of a statin for prevention of HF hospitalizations.[18]

In interpreting the results of the ALLHAT-LLT, consideration should be given to the substantial drop-in rate of patients likely treated with statins other than pravastatin.

IV. Hypotheses

Table 3 presents the specific hypotheses for various outcomes with accompanying estimates of event rates and power. The estimated 9-year and 10-year rates for cardiovascular disease (CVD) mortality and total mortality, CHD, stroke, HF, CVD, and ESRD in the chlorthalidone and usual care groups are based on the observed results in the original study extended through 9 and 10 years of follow-up. Cancer mortality and HF mortality rate estimates are based upon the proportion of total mortality that these outcomes represented at the end of the main trial for the chlorthalidone and usual care groups. HF case-fatality rates are also based on those seen within the trial at present but extended into the post-trial follow-up period for the chlorthalidone and usual care groups. Since the average follow-up time to first HF hospitalization was 2.9 years, the case-fatality rate projections will be for 6 and 7 years rather than 9 and 10 years. The effect sizes for the comparisons are based on the achieved sample size and the use of 90% power.

The comparisons within subgroups are also based on the observed in-trial rates and the achieved sample size within these subgroups. The CHD, stroke, and HF non-fatal outcomes associated with hospitalization will be based upon 82% of the total cohort which we estimate captures those individuals available to be followed by databases – VA, Health Care Financing Administration (now the Centers for Medicare and Medicaid Services [CMS]), etc. In the Cardiovascular Health Study (CHS), the agreement rate between HCFA and CHS was high for MI; 84.8% of HCFA-eligible incident MI's were ascertained by both CHS and HCFA. These results for other endpoints include 84.8% for angina, 84.0% for HF, 80.0% for PVD, and 81.0% for stroke.[19] About 15.8% of all HCFA-eligible incident events were ascertained by CHS but not by HCFA. We believe that these agreement rates are high enough to allow for meaningful comparisons across treatment groups using only events ascertained through database searches.

It should be noted that silent MI (a component of the CHD endpoint) cannot be determined during extended follow-up.

Hypotheses regarding the effect of in-trial and post-trial occurrences of hospitalized nonfatal heart failure (Hypotheses 20-23) on subsequent events will be analyzed using time-dependent covariate analyses. Post-trial new nonfatal hospitalized HF will be ascertained through searches of the HCFA/VA databases.

Hypotheses regarding the effect of incident diabetes at 2 years (Hypotheses 17-19) on subsequent events will be analyzed using Cox regression models beginning at 2 years.

There are several factors that relate some of the hypotheses:

- Over time, ALLHAT participants develop diabetes, which is an important risk factor for CHD and HF.
- Over time, ALLHAT participants develop CHD, which is an important risk factor for HF.

- HF and stroke both have high case fatality rates and are important risk factors for CVD and total mortality.

These possible relationships will be explored as time-dependent variables in the context of evaluating risk factors for CHD, HF, and total mortality.

V. Methods

a. Sources of outcome data

All-cause mortality, mortality by cause, and specific nonfatal events as listed previously will be the outcomes of interest for our analysis. Table 4 lists the sources of outcome data.

All-cause mortality will be ascertained from the National Death Index (NDI). Data on all-cause mortality will be obtained from the National Death Index using Social Security number, name, sex, and date of birth as matching criteria, and cause of death will be ascertained from the NDI Plus database.

Nonfatal events will be ascertained from Centers for Medicare and Medicaid Services (CMS [formerly HCFA]) and the VA databases. These outcome data are only available for participants with valid Medicare numbers and/or who have data available from the VA (82% of all participants). Renal endpoints will be ascertained from the US Renal Data System database.

Due to the lack of access to a Canadian database that would provide outcome information, and lack of identifying information (i.e., a health system number) for Canadian participants, participants randomized in Canada will not be included in post-trial analyses.

b. Definitions

A death identified through passive surveillance is defined as a possible match through NDI or Social Security that is verified at the CTC after receipt of a death certificate from the state. Death certificates are used only for verification of participant identity. Causes of death are obtained from NDI Plus. However, if a cause of death is not available from NDI, the death certificate will be coded by a nosologist using the ICD10 coding scheme. Causes of death will be classified using the ICD10 code, translated to ICD9 and classified as specified in Table 3.

Nonfatal events will be identified through CMS and VA data using the provided ICD9 codes from those sources and classified as in Table 3. Renal endpoints will be determined from CMS and the VA as well as from the US Renal Data System.

c. Statistical Analyses

The proposed analyses will include evaluation of the effect of treatment group on the risk for the primary and secondary outcomes using Cox regression. The follow-up period includes both the randomized trial (mean duration of follow-up, 4.9 years) and subsequent follow-up during the extension period. Refer to table 3 to see the projected percent reduction using this data for each hypotheses proposed. Death will be censored as a competing event in the analyses that include non-fatal events. All

analyses will be performed on an intention-to-treat basis, i.e., participants are analyzed according to their original randomized treatment group. Hazard ratios will be adjusted for baseline factors based on analyses showing which factors were independently associated with the events of interest. Finally, the proportional hazards assumption of the Cox regression models will be checked. Pre-specified tests for interactions will be conducted to determine whether the effects of the treatment intervention differ between subgroups, defined by age, race, sex, or diabetes status.

Time-dependent Cox regression will be used to estimate the hazard ratios associated with the treatment intervention separately for the trial period and the post-trial follow-up period.

VI. Operations

a. Long-Term Follow-up (2002–2006) of the ALLHAT Study

The investigational review board (IRB) of The University of Texas Health Science Center approved the long-term follow-up study. Several databases will be pursued as described in Table 4.

- National Death Index (NDI)
- Social Security Administration (SSA)
- Centers for Medicare and Medicaid Services (CMS)
- Veterans' Administration (VA)
- USRDS

b. Timeline

The average follow-up time in ALLHAT was 4.9 years (4.8 in LLT). Table 4 describes the additional follow-up expected from the databases described above. Follow-up through the NDI and SSA through 2006 will provide an additional 4.75 years of follow-up for a total of 9.65 years average follow-up. The timeline in Figure 1 shows how the additional follow-up data fit into ALLHAT as a whole, and also shows the activities related to obtaining and verifying the additional data (e.g., obtaining death certificates and causes of death), and completion of papers and presentations.

During the main trial, the Clinical Trials Center received possible matches from the NDI and SSA and sent requests to various states for death certificates. The purpose of this was to verify that the decedent was, in fact, the matched ALLHAT participant. Only those with their identities thus verified by death certificate review were counted as deaths for the antihypertensive and lipid-lowering final papers; the cause of death was from NDI Plus. Those potentially matched through NDI or SSA, but for whom death certificates were not received, were not counted as dead, but were counted in a separate category of "dead pending confirmation" (so as to not be counted as lost to follow-up). Many participants who were "dead pending confirmation" at the time of the final papers have since been confirmed. The Clinical Trials Center will continue to request death certificates from the states for participants who are potential SSA or NDI matches during the extension. Death certificates are used for verification of participant identity as described in Section V.b. However, it is anticipated that by the time of the final extension data lock (December 31, 2008), over half of the 2006 death certificates will still be missing (along with some with earlier dates of death). For those without death certificates as of the data lock, we will count those as deceased in the final extension results. It is expected that out of these "unconfirmed" deaths, 94-96% would eventually be confirmed to be the matched ALLHAT

participant, with no difference expected across treatment groups. Causes of death will be obtained from NDI Plus.

c. Papers and presentations

Although the final decision regarding presenting and publishing data from the extended follow-up belong with the Editorial Subcommittee and Steering Committee, it is suggested that we consider presenting the 10-year results of the AHT and LLT at the American College of Cardiology meeting in March 2009, with simultaneous publication.

V. References

1. Davis BR, Cutler JA, Gordon DJ, Furberg CD, Wright JT, Cushman WC, Grimm RH, LaRosa J, Whelton PK, Perry HM, Alderman MA, Ford CE, Oparil S, Francis C, Proschan M, Pressel S, Black HR, Hawkins CM; ALLHAT Research Group. Rationale and design for the Antihypertensive and Lipid Lowering Treatment to Prevent Heart Attack Trial (ALLHAT). *Am J Hypertens*. 1996; 9:342-360.
2. Grimm RH, Margolis KL, Papademetriou V, Cushman WC, Ford CE, Bettencourt J, Alderman MH, Basile JN, Black HR, DeQuattro V, Eckfeldt J, Hawkins CM, Proschan M, for the ALLHAT Collaborative Research Group. Baseline characteristics of the 42,448 high risk hypertensives enrolled in the Antihypertensive and Lipid-Lowering Treatment to Prevent Heart Attack Trial (ALLHAT). Hypertension 2001; 37:19-27.
3. The ALLHAT Officers and Coordinators for the ALLHAT Collaborative Research Group. Major outcomes in high-risk hypertensive patients randomized to angiotensin-converting enzyme inhibitor or calcium channel blocker vs diuretic: the Antihypertensive and Lipid-Lowering Treatment to Prevent Heart Attack Trial (ALLHAT). *JAMA*. 2002; 288:2981-2997.
4. Kostis JB, Wilson AC, Freudenberger RS, Cosgrove NM, Pressel SL, Davis BR. Long-term effect of diuretic-based therapy on fatal outcomes in subjects with isolated systolic hypertension with and without diabetes. Am J Cardiol 2005; 95:29-35.
5. The ALLHAT Officers and Coordinators for the ALLHAT Collaborative Research Group. Major cardiovascular events in hypertensive patients randomized to doxazosin vs chlorthalidone: The Antihypertensive and Lipid-Lowering Treatment to Prevent Heart Attack Trial (ALLHAT). *JAMA*. 2000; 283:1967-1975.
6. The ALLHAT Officers and Coordinators for the ALLHAT Collaborative Research Group. Diuretic versus α-blocker as first-step antihypertensive therapy. Final results from the Antihypertensive and Lipid-Lowering Treatment to Prevent Heart Attack Trial (ALLHAT). *Hypertension* 2003; 42:239-246.
7. Barzilay JI, Davis BR, Bettencourt J, Margolis KL, Goff DC, Black H, Habib G, Ellsworth A, Force RW, Wiegmann T, Ciocon JO, Basile JN, for the ALLHAT Collaborative Research Group. Cardiovascular outcomes using doxazosin vs. chlorthalidone for the treatment of hypertension in older adults with and without glucose disorders: A report from the ALLHAT study. J Clin Hypertens 2004; 6:116-125.
8. The ALLHAT Officers and Coordinators for the ALLHAT Collaborative Research Group. Major Outcomes in Moderately Hypercholesterolemic, Hypertensive Patients Randomized to Pravastatin vs

Usual Care: The Antihypertensive and Lipid-Lowering Treatment to Prevent Heart Attack Trial (ALLHAT-LLT). JAMA. 2002; 288:2998-3007.

9. Hypertension Detection and Follow-up Program Cooperative Group. Persistence of reduction in blood pressure and mortality of participants in the Hypertension Detection and Follow-up Program. JAMA 1988; 259:2113-2122.
10. Multiple Risk Factor intervention Trial Research Group. Mortality after 10½ years for hypertensive participants in the Multiple Risk Factor Intervention Trial. Circulation 1990; 82:1616-1628.
11. LIPID Study Group (Long-term Intervention with Pravastatin in Ischemic Disease). Long-term effectiveness and safety of pravastatin in 9014 patients with coronary heart disease and average cholesterol concentrations: the LIPID trial follow-up. Lancet 2002; 359(9315):1379-1387. [erratum appears in Lancet 2002 Nov 2;360(9343):1430]
12. Pedersen TR, Wilhelmsen L, Faergeman O, Strandberg TE, Thorgeirsson G, Troedsson L, Kristianson J, Berg K, Cook TJ, Haghfelt T, Kjekshus J, Miettinen T, Olsson AG, Pyorala K, Wedel H. Follow-up study of patients randomized in the Scandinavian Simvastatin Survival Study (4S) of cholesterol lowering. Am J Cardiol 2000; 86:257-262.
13. The Lipid Research Clinics Investigators. The Lipid Research Clinics Coronary Primary Prevention Trial. Results of 6 years of post-trial follow-up. Arch Intern Med 1992; 152:1399-1410.
14. Barzilay JI, Davis BR, Cutler JA, Pressel SL, Whelton PK, Basile J, Margolis KL, Ong ST, Sadler LS, Summerson J, for the ALLHAT Collaborative Research Group. Fasting glucose levels and incident diabetes mellitus in older nondiabetic adults randomized to receive 3 different classes of antihypertensive treatment. Arch Intern Med 2006; 166:2191-2201.
15. Rahman M, Pressel S, Davis BR, Nwachuku C, Wright JT Jr, Whelton PK, Barzilay J, Batuman V, Eckfeldt JH, Farber M, Henriquez M, Kopyt N, Louis GT, Saklayen M, Stanford C, Walworth C, Ward H, Wiegmann T, for the ALLHAT Collaborative Research Group, Renal Outcomes in High-Risk Hypertensive Patients treated with an Angiotensin-Converting Enzyme Inhibitor, or Calcium Channel Blocker versus a Diuretic. A report from The Antihypertensive and Lipid-Lowering Treatment to Prevent Heart Attack Trial (ALLHAT). Arch Internal Med 2005; 165:936-946.
16. Sabbatini M, Leonardi A, Testa R, Tomassoni D, Vitaioli L, Amenta F.Effects of dihydropyridine-type Ca2+ antagonists on the renal arterial tree in spontaneously hypertensive rats. J Cardiovasc Pharmacol 2002; 39:39-48.
17. Agodoa LY, Appel L, Bakris GL, Beck G, Bourgoignie J, Briggs JP, Charleston J, Cheek D, Cleveland W, Douglas JG, Douglas M, Dowie D, Faulkner M, Gabriel A, Gassman J, Greene T, Hall Y, Hebert L, Hiremath L, Jamerson K, Johnson CJ, Kopple J, Kusek J, Lash J, Lea J, Lewis JB, Lipkowitz M, Massry S, Middleton J, Miller ER 3rd, Norris K, O'Connor D, Ojo A, Phillips RA, Pogue V, Rahman M, Randall OS, Rostand S, Schulman G, Smith W, Thornley-Brown D, Tisher CC, Toto RD, Wright JT Jr, Xu S. Effect of ramipril vs amlodipine on renal outcomes in hypertensive nephrosclerosis: a randomized controlled trial. JAMA 2001; 285:2719-2728.
18. LaRosa JC, Grundy SM, Waters DD, Shear C, Barter P, Fruchart JC, Gotto AM, Greten H, Kastelein JJ, Shepherd J, Wenger NK, for the Treating to New Targets (TNT) Investigators. Intensive lipid lowering with atorvastatin in patients with stable coronary disease. N Engl J Med 352:1425-1435, 2005.

19. Ives DG, Fitzpatrick, AL, Bild DE, Psaty BM, Kuller LH, Crowley PM, Cruise RG, Theroux S. Surveillance and Ascertainment of Cardiovascular Events – The Cardiovascular Health Study. Ann Epidemiol 1995; 5:278-285.

20. Law MR, Thompson SG, Wald NJ. Assessing possible hazards of reducing serum cholesterol. BMJ 1994;308:373-379.

Table 1 – ALLHAT Outcomes

1. Death
   a. Definite myocardial infarction
   b. Definite coronary heart disease
   c. Possible coronary heart disease
   d. Stroke
   e. Congestive heart failure
   f. Other cardiovascular disease
   g. Cancer
   h. Accident, suicide, or homicide
   i. Other noncardiovascular cause
   j. Unknown cause
2. Myocardial infarction
3. Stroke
4. Angina (hospitalized or treated)
5. Heart failure (hospitalized or treated)
6. Peripheral arterial disease (hospitalized or treated)
7. New cancer diagnosis (hospitalized or treated)
8. Accident or attempted suicide (hospitalized or treated)
9. Left ventricular hypertrophy (biennial study, electrocardiogram)
10. Renal function
    a. Slope of the reciprocal of serum creatinine level versus time
    b. End-stage renal disease (initiation of chronic renal dialysis or kidney transplant)
11. Quality of life
12. Medical care use

Table 2 – Secondary Hypotheses for the ALLHAT Trial Components

Antihypertensive Trial—The following endpoints (or their incidence) will be reduced in patients randomized to receive amlodipine, lisinopril, or doxazosin relative to those receiving chlorthalidone:

1. All-cause mortality
2. Combined coronary heart disease (CHD or revascularization procedures or hospitalized angina)
3. Stroke
4. Combined cardiovascular disease (CHD or stroke or coronary revascularization procedures or angina [hospitalized or medically treated] or HF [hospitalized or medically treated] or peripheral arterial disease [hospitalized or outpatient revascularization procedure]),
5. Left ventricular hypertrophy by ECG
6. Renal disease
   a. Slope and reciprocal of serum creatinine
   b. End-stage renal disease (initiation of chronic renal dialysis or kidney transplant)
7. Health-related quality of life
8. Major costs of medical care

Lipid-Lowering Trial—The following endpoints (or their incidence) will be reduced in patients randomized to receive pravastatin relative to those receiving usual care:

1. The combined incidence of CHD death and nonfatal myocardial infarction, especially in certain subgroups, e.g., blacks, patients over age 65 (the original Cholesterol Reduction in Seniors Program [CRISP] hypothesis[20]), type 2 diabetics, and women
2. Changes in the biennial study ECG indicative of myocardial infarction
3. Cause-specific mortality (e.g., cancer, trauma)
4. Total and site-specific cancer incidence
5. Health-related quality of life
6. Major costs of medical care

CHD, coronary heart disease; HF, heart failure; ECG, electrocardiogram.

**Table 3. Percent Reductions from Comparator Treatment for 90% Power (The general null hypothesis is that there is no difference in event rates at an average of 9-10 years of follow-up.)**					
		9 Years		10 Years	
Statement of hypothesis	Primary outcome variable	Estimated rate of outcome	Relative Risk Reduction	Estimated rate of outcome	Relative Risk Reduction
**Antihypertensive Trial**					
**1. CVD Mortality**					
Is there a CVD mortality difference at an average of 9 or 10 years of follow-up?					
1a – amlodipine vs chlorthalidone<br>1b – lisinopril vs chlorthalidone<br>1c – doxazosin vs chlorthalidone	CVD mortality[1]	14%	11.9%	16%	11.0%
**2. Total Mortality / Non-CVD mortality / Cancer mortality / Other causes**					
Is there a total mortality difference at an average of 9 or 10 years of follow-up?					
2a – amlodipine vs chlorthalidone<br>2b – lisinopril vs chlorthalidone	Total mortality<br>Cancer mortality[3]	29%<br>6.5%	7.6%<br>17.8%	33%<br>7.4%	6.9%<br>16.7%
2c – doxazosin vs chlorthalidone	Total mortality	29%	7.6%	34%	6.9%
**3. CVD Endpoint (CVD mortality or hospitalized non-fatal MI or hospitalized non-fatal HF or hospitalized non-fatal stroke)**					
Is there a CVD endpoint difference at an average of 9 or 10 years of follow-up?					
3a – amlodipine vs chlorthalidone<br>3b – lisinopril vs chlorthalidone<br>3c – doxazosin vs chlorthalidone	CVD endpoint[4]	30.0%	8.1%	33.0%	7.6%
**4. CHD Endpoint (CHD death or hospitalized non-fatal MI)**					
Is there a CHD endpoint difference at an average of 9 or 10 years of follow-up?					
4a – amlodipine vs chlorthalidone<br>4b – lisinopril vs chlorthalidone<br>4c – doxazosin vs chlorthalidone	CHD[4]	18%	11.2%	20%	10.6%

**5. Hospitalized or fatal stroke**					
Is there a difference in hospitalized stroke at an average of 9 or 10 years of follow-up?					
5a – amlodipine vs chlorthalidone<br>5b – lisinopril vs chlorthalidone<br>5c – doxazosin vs chlorthalidone	Stroke[4]	8.0%	17.5%	9.0%	16.5%
**6. Hospitalized heart failure**					
Is there a difference in hospitalized heart failure at an average of 9 or 10 years of follow-up?					
6a – amlodipine vs chlorthalidone<br>6b – lisinopril vs chlorthalidone<br>6c – doxazosin vs chlorthalidone	Hospitalized/fatal HF[4]	10.5%	15.2%	12%	14.1%
**7. Hospitalized GI bleeding**					
Is there a difference in hospitalized GI bleeding at an average of 9 or 10 years of follow-up?					
7a – amlodipine vs chlorthalidone<br>7b – lisinopril vs chlorthalidone<br>7c – doxazosin vs chlorthalidone	Hospitalized GI bleed[4]	13.0%	13.5%	15.0%	18.7%
**8. ESRD (initiation of chronic renal dialysis or kidney transplant)**					
Is there a difference in ESRD at an average of 9 or 10 years of follow-up?					
8a – amlodipine vs chlorthalidone<br>8b – lisinopril vs chlorthalidone<br>8c – doxazosin vs chlorthalidone	ESRD through USRDS[4]	3.2%	27.5%	3.7%	25.7%
**9. AHT Subgroups**					
Are there mortality or morbidity differences seen in the above comparisons in the pre-specified subgroups - [age (<65, 65+), sex, race (Black, non-Black), and history of diabetes (yes/no)] at an average of 10 years of follow-up?<br>9a – amlodipine vs chlorthalidone<br>9b – lisinopril vs chlorthalidone<br>9c – doxazosin vs chlorthalidone	Total mortality (Blacks)	30.4%	12.2%	34.3%	11.2%
	CVD mortality[1] (Blacks)	13.8%	19.7%	15.5%	18.5%
	Hospitalized/fatal HF[4] (Blacks)	10.5%	24.9%	12%	23.2%

Lipid-Lowering Trial					
**10. Total Mortality**					
Is there a total mortality difference between pravastatin and usual care at an average of 9 or 10 years of follow-up?	Total mortality	28%	10.4%	32%[5]	9.3%
**11. CVD Mortality**					
Is there a CVD mortality difference between pravastatin and usual care at an average of 9 or 10 years of follow-up?	CVD mortality[1]	13%	16.2%	15%	14.9%
**12. CHD Endpoint (CHD death or hospitalized non-fatal MI)**					
Is there a CHD endpoint difference between pravastatin and usual care at an average of 9 or 10 years of follow-up?	CHD[4]	16.5%	15.5%	18.5%	14.5%
**13. Hospitalized or fatal stroke**					
Is there a difference in hospitalized stroke between pravastatin and usual care at an average of 9 or 10 years of follow-up?	Stroke[4]	8.5%	22.2%	9.5%	20.9%
**14. Cause-specific mortality (especially cancer)**					
Are there differences in CVD and cancer mortality between pravastatin and usual care at an average of 9 or 10 years of follow-up?	CVD mortality[1]<br>Cancer mortality[3]	13%<br>6.3%	16.2%<br>23.7%	15%<br>7.2%	14.9%<br>22.1%
**15. ESRD (initiation of chronic renal dialysis or kidney transplant), total and by eGFR at baseline.**					
Are there differences in ESRD between pravastatin and usual care at an average of 9 or 10 years of follow-up?	ESRD through USRDS[4]	3.2%	35.9%	3.8%	33.1%
**16. LLT Subgroups**					
Are there mortality or morbidity differences seen in the above comparisons in the pre-specified subgroups - [age (<65, 65+), sex, race (Black, non-Black), and history of diabetes (yes/no)] at an average of 10 years of follow-up?	Total mortality (Blacks)	30.5%	15.5%	35.5%	13.9%
	CVD mortality[1] (Blacks)	13.9%	24.8%	16.1%	22.8%

Additional Hypotheses Regarding Diabetes					
**17. Total Mortality**					
Does long term total mortality differ by diabetes status (no diabetes, diabetes at baseline, new onset diabetes, at 2 years during ALLHAT)<br>17a – amlodipine vs chlorthalidone<br>17b – lisinopril vs chlorthalidone<br>17c – doxazosin vs chlorthalidone	Total mortality<br>no diabetes<br><br>diabetes at baseline<br><br>new onset diabetes	16.1%<br><br>18.8%<br><br>15.7%	23.6%<br><br>26.2%<br><br>78.1%	22.5%<br><br>26.4%<br><br>21.8%	19.4%<br><br>21.4%<br><br>67.7%
**18. CVD Mortality**					
Does long term CVD mortality differ by diabetes status (no diabetes, diabetes at baseline, new onset diabetes, at 2 years during ALLHAT)<br>18a – amlodipine vs chlorthalidone<br>18b – lisinopril vs chlorthalidone<br>18c – doxazosin vs chlorthalidone	CVD mortality[1]<br>no diabetes<br><br>diabetes at baseline<br><br>new onset diabetes	7.1%<br><br>9.2%<br><br>7.4%	35.9%<br><br>38.0%<br><br>>99%	9.9%<br><br>12.9%<br><br>10.3%	30.5%<br><br>32.1%<br><br>91.5%
**19. CVD Endpoint (CVD mortality or hospitalized non-fatal MI or hospitalized non-fatal HF or hospitalized non-fatal stroke)**					
Does long term CVD events differ by diabetes status (no diabetes, diabetes at baseline, new onset diabetes, at 2 years during ALLHAT)<br>19a – amlodipine vs chlorthalidone<br>19b – lisinopril vs chlorthalidone<br>19c – doxazosin vs chlorthalidone	CVD endpoint[4]<br>no diabetes<br><br>diabetes at baseline<br><br>new onset diabetes	17.3%<br><br>23.9%<br><br>21.4%	24.5%<br><br>24.8%<br><br>72.9%	20.3%<br><br>28.3%<br><br>25.6%	22.4%<br><br>22.3%<br><br>67.2%

**Additional Hypotheses Regarding Heart Failure**					
**20. Case Fatality**					
Does the case fatality rate differ by treatment group for those who develop hospitalized HF in ALLHAT? By subgroup?<br>20a – amlodipine vs chlorthalidone<br>20b – lisinopril vs chlorthalidone<br>20c – doxazosin vs chlorthalidone	HF case fatality	59%<br>59% (Blacks)	17.0%<br>29.3%	63% [7]<br>64% (Blacks)	15.8%<br>26.8%
**21. Case Fatality by LV systolic function[8]**					
Does the case fatality rate differ by treatment group for those who develop hospitalized HF with preserved/impaired/unknown left ventricular systolic function?<br>21a – amlodipine vs chlorthalidone<br>21b – lisinopril vs chlorthalidone<br>21c – doxazosin vs chlorthalidone	HF case fatality[8]	Pending finalization and publication of relevant ALLHAT paper.			
**22. Total Mortality by HF Status**					
Does long term total mortality differ by HF status (no HF, new onset HF during ALLHAT)<br>22a – amlodipine vs chlorthalidone<br>22b – lisinopril vs chlorthalidone<br>22c – doxazosin vs chlorthalidone	Total mortality	57%<br>(in HF Group)	8.1%	62%<br>(HF Group)	7.3%
**23. CVD Mortality by HF Status**					
Does long term CVD mortality differ by HF status (no HF, new onset HF during ALLHAT)<br>23a – amlodipine vs chlorthalidone<br>23b – lisinopril vs chlorthalidone<br>23c – doxazosin vs chlorthalidone	CVD mortality[1]	40%<br>(HF Group)	11.3%	44%<br>(HF Group)	10.4%

Additional Hypotheses Regarding Renal Disease					
**24. ESRD (initiation of chronic renal dialysis or kidney transplant) by eGFR**					
Is there a difference in ESRD at an average of 9 or 10 years of follow-up across the spectrum of eGFR at baseline?					
24a – amlodipine vs chlorthalidone<br>24b – lisinopril vs chlorthalidone<br>24c – doxazosin vs chlorthalidone	ESRD through USRDS[4]<br>GFR <60<br>GFR=60-89.9<br>GFR=90+	 <br>11.3%<br>1.6%<br>1.2%	 <br>34.1%<br>51.1%<br>79.0%	 <br>12.9%<br>1.8%<br>1.5%	 <br>31.9%<br>47.5%<br>71.9%
**25. CVD Endpoint (CVD mortality or hospitalized non-fatal MI or hospitalized non-fatal HF or hospitalized non-fatal stroke) by eGFR**					
Is there a difference in CVD endpoints at an average of 9 or 10 years of follow-up across the spectrum of eGFR at baseline?					
25a – amlodipine vs chlorthalidone<br>25b – lisinopril vs chlorthalidone<br>25c – doxazosin vs chlorthalidone	ESRD through USRDS[4]<br>GFR <60<br>GFR=60-89.9<br>GFR=90+	 <br>41.5%<br>28.9%<br>23.7%	 <br>15.5%<br>11.3%<br>18.9%	 <br>45.3%<br>31.8%<br>26.2%	 <br>14.4%<br>10.6%<br>17.8%

Notes:
[1] CVD mortality is estimated at 40 - 49% of total mortality, depending on subgroup.
[2] The estimated mortality rate in the chlorthalidone group at 6 years is about 18%.
[3] Cancer mortality is assumed 22.5% of total mortality.
[4] Assumes follow-up on 82% of cohort. Silent MI is not available during extended follow-up.
[5] The estimated mortality rate in the usual care group at 6 years is about 16%.
[6] Incident diabetes is not available during extended follow-up.
[7] The estimated case-fatality rates are for 6 and 7 years post HF hospitalization because the mean time to nonfatal HF event was 2.5 years. Likewise, the post-HF mortality and CVD mortality are restricted to 6 and 7 years post HF event.
[8] The data for this hypothesis is currently being validated.

TABLE 4
Assuming End Date 3/31/2009
ALLHAT EXTENSION
SUMMARY OF OUTSIDE DATABASE SEARCHES
FOR VITAL STATUS AND STUDY EVENTS

Database	Purpose	Status & plans
SSA	Dead, alive during benefits year	CTC has searches through September 2004<br>Submit search June 2007 for remainder of 2004 and for 2005 and 2006 status<br>(Verified deaths will be "searched" through the NDI for death certificate information and causes of death)
NDI	Death, cause of death	CTC has NDI Plus through 2002<br>Planned searches:<br>• June 2007 – 2003 through 2005 deaths & causes<br>• May 2008 – 2006 deaths & causes<br>• All searches include information to be verified from SSA searches
CMS	Hospitalized events	June 2007 – Request 2001-2006 events
VA	Hospitalized events	June 2007 – Request 2001-2006 events
USRDS	ESRD cases and related data; for several ALLHAT papers	July 2007 – Request 1994-2006 events

Table 5
COMPLETE LISTING OF ICD9 CODES MAPPED TO ALLHAT EVENTS
Revised March 28, 2002

INFECTIOUS / PARASITIC DISEASES 001-139

Nonfatal – Not applicable
Fatal – Other noncardiovascular

NEOPLASMS 140-239

Codes	Description	Nonfatal	Fatal
140.x through 208.x	Malignant neoplasms	Cancer	Cancer
Except:<br>173.x – Skin neoplasm NEC, primary<br>196.x – Secondary cancers<br>197.x – Secondary cancers<br>198.x – Secondary cancers<br>199.x – Unspecified primary and secondary cancers		(Not applicable)	Cancer
209.x-229.x	Benign neoplasms	(Not applicable)	Other noncardiovascular
230.x through 234.x	In-situ cancers	Cancer	Cancer
Except:<br>232.x – In situ skin cancer		(Not applicable)	Cancer
235.x through 238.x (was 239.x)	Cancers of uncertain behavior	(Not applicable)	Cancer
239.x	Unspecified cancer	(Not applicable)	Cancer

ENDOCRINE, NUTRITIONAL, METABOLIC, IMMUNITY – 240-279

Nonfatal – Not applicable except as noted below
Fatal – All codes are other noncardiovascular death

Codes	Description	Nonfatal	Fatal
250.7	Diabetes with peripheral circulatory disorders	Hospitalized lower extremity arterial disease	

BLOOD AND BLOOD-FORMING ORGANS – 280-289

Nonfatal: Not applicable
Fatal: All codes are other noncardiovascular death

MENTAL DISORDERS – 290-319

Nonfatal: Not applicable
Fatal: All codes are other noncardiovascular death

DISEASES OF THE NERVOUS SYSTEM AND SENSE ORGANS – 320-389

Nonfatal: Not applicable
Fatal: All codes are other noncardiovascular death

DISEASE OF THE CIRCULATORY SYSTEM – 390-459

Codes	Description	Nonfatal	Fatal
402.01, 402.11, 402.91	HF	HF	HF
410.x	MI	MI	MI
411.1, 413.x	Angina	Hospitalized angina	Probable CHD
411.x, 412.x, 414.x			Probable CHD
415.0	HF	HF	HF
416.1			Other noncardiovascular
427.41, 427.42, 427.5			Probable CHD
428.x	HF	HF	HF
430.x-434.x, 436.x Please note that 435.9 was previously coded as stroke, but this is incorrect	Stroke	Stroke	Stroke
440.2x, 440.3x, 442.2, 442.3, 443.9, 444.22, 444.81	Peripheral vascular disease	Hospitalized lower extremity arterial disease?	Other cardiovascular
454.x, 455.0-8, 456.0-2, 458.x			Other cardiovascular
457.x, 455.9, 456.3-.8			Other noncardiovascular
459.0 – Intra-abdominal hemorrhage	GI bleed	GI bleed	Other cardiovascular
All other 390-459	Other cardiovascular		Other cardiovascular

DISEASES OF THE RESPIRATORY SYSTEM – 460-519
DISEASES OF THE DIGESTIVE SYSTEM – 529-579
DISEASES OF THE GENITOURINARY SYSTEM – 580-629
COMPLICATIONS OF PREGNANCY, CHILDBIRTH AND THE PUERPERIUM – 630-676
DISEASES OF THE SKIN AND SUBCUTANEOUS TISSUES – 680-709
DISEASES OF THE MUSCULOSKELETAL SYSTEM & CONNECTIVE TISSUES – 710-739
CONGENITAL ABNORMALITY – 740-759
PERINATAL CONDITIONS – 760-779
SYMPTOMS, SIGNS, AND ILL-DEFINED CONDITIONS – 780-799

Nonfatal: Not applicable except as noted below
Fatal: Other noncardiovascular except as noted below

Codes	Description	Nonfatal	Fatal
578.x – (originally only 578.9)	GI hemorrhage	GI bleed	
531,0, 531.2, 531.4	Gastric ulcer<br>- Acute with hemorrhage<br>- Acute with hemorrhage and perforation<br>- chronic or unspecified with hemorrhage	GI bleed	
531.6	Gastric ulcer, chronic or unspecified with hemorrhage and perforation	GI bleed	
532.0/2/4/6	Duodenal ulcer, acute or chronic or unspecified, with hemorrage	GI bleed	
533.0/2/4/6	Peptic ulcer, site unspecified, acute or chronic or unspecified, with hemorrage	GI bleed	
534.0/2/4/6	Gastrojejunal ulcer, acute or chronic or unspecified, with hemorrage	GI bleed	
535.01, .11, .21, .31, .41, .51, .61	Gastritis/duodenitis with hemorrhage	GI bleed	
580-593			Kidney disease
745.2, 745.4, 745.9, 747.22, 746.x, 746.xx			Other cardiovascular
753.1, 753.3			Kidney disease
785.4	Gangrene of lower or upper extremity	Hospitalized lower extremity arterial disease?	Other cardiovascular
780.2 785.0/1/2/3, 785.51,786.50/51/59, 796.2/3			Other cardiovascular
798.1, 798.2, 798.9, 799.1			Probable CHD

INJURY AND POISONING – 800-999

Nonfatal: Accident or attempted suicide, except as noted
Fatal: Accident, suicide, homicide, except as noted

Codes	Description	Nonfatal	Fatal
996.0x, 996.1, 996.61, 996.62, 996.72, 996.83, 997.1			Other cardiovascular
996.73, 996.81			Kidney disease
997.02		Stroke, GI bleed	Stroke, GI bleed
998.1, 998.11, 998.12		GI bleed	GI bleed
997.2, 997.6, 999.2		Hospitalized lower extremity arterial disease?	Other cardiovascular

SUPPLEMENTARY CLASSIFICATION OF FACTORS INFLUENCING HEALTH STATUS AND CONTACT WITH HEALTH SERVICES – V01-V82

Nonfatal: Not applicable
Fatal: Unknown cause of death

SUPPLEMENTARY CLASSIFICATION OF EXTERNAL CAUSES OF INJURY AND POISONING – E800-E999

Nonfatal: Accident or attempted suicide
Fatal: Accident, suicide or homicide

PROCEDURES

Codes	Description	Procedure
36.0x		PTCA
Other 36.xx	Operations on vessels of heart, coronary stent, atherectomy	CABG
38.08, .18, .38, .48, .68	Incision, excision, and occlusion of lower limb arteries	Lower extremity revascularization /bypass/ angioplasty
39.25	Aorta-femoral bypass	Lower extremity arterial revascularization/bypass/ angioplasty
39.29	Other peripheral vascular shunt or bypass	
39.95	Kidney dialysis	Kidney dialysis
55.6x	Kidney transplant	Kidney transplant
84.1x	Leg amputation	Hospitalized lower extremity arterial disease
99.03, 99.04, 99.05	Transfusion – general, packed red blood cells, platelets	GI bleed
99.10	Streptokinase	MI
99.20	Thrombolytic procedure	MI

Figure 1
ALLHAT Timeline

	Jan	Feb	Mar	Apr	May	Jun	Jul	Aug	Sep	Oct	Nov	Dec
**1994**		1st AHT rz		1st LLT rz								
**1998**	Last AHT rz				Last LLT rz							
**2000**	Decision to end dox	Dox c/o begins		1st dox paper	Dox c/o ends							
**2001**										Study c/o begins		
**2002**			Study c/o ends									AHT/LLT final papers
**2003**									Final dox paper			
**2004**												
**2005**												
**2006**												
**2007**						Request 2003-2005 NDI & 2004-2006 SSA files; request CMS 2001-6 & VA 2001-6; Identify conveners; Charge to writing groups	Request USRDS 1994-2006; start drafting final extension papers; Identify writing groups	Request 2003-2005 NDI & 2004-2006 SSA DC's through states; 1st conference call of writing groups			Run 1st post-trial analyses	
**2008**		Expect ½ of last round of requested NDI & SSA DC's; 2nd draft of papers			NDI 2006		Request 2006 NDI DC's through states; run 2nd round of post-trial analyses		3rd draft of papers			EXTENSION DATA LOCK – 12/31/2008
**2009**	Final post-trial analyses	Final papers	ACC presentation. Submission of papers.									

Antihypertensive and Lipid Lowering Treatment to Prevent Heart Attack Trial (ALLHAT)

Protocol

<u>Revised:</u>
March 1995
May 1995
April 1998
April 2000

Antihypertensive and Lipid Lowering Treatment to Prevent Heart Attack Trial (ALLHAT)

Protocol

Table of Contents

Page

I. Overview 2
II. Background 4
III. Hypotheses and Study Power 10
IV. Eligibility and Exclusions 13
V. Recruitment 17
VI. Antihypertensive Intervention 22
VII. Cholesterol-Lowering Intervention 26
VIII. Laboratory Measurements 28
IX. Outcome Measurements 30
X. Study Organization 33
XI. Data Management and Analysis 38
XII. Vanguard Phase 43
XIII. References 44
Appendix I - Sample Size Calculations 49
Appendix II - Detailed Definitions of Coronary Death, Nonfatal Acute Myocardial Infarction and Stroke 59
Appendix III – Electrocardiographic Criteria for Silent Myocardial Infarction, Ischemia, Left Ventricular Hypertrophy, and Bundle Branch Block 61
→ Appendix IV – Quality Control Evaluation of Hospitalized Myocardial Infarctions (MI) 68
Addendum 1 – Revised Power for Lipid-Lowering Portion of ALLHAT 74
Addendum 2 – Monitoring Boundaries 77

I. Overview

This protocol describes a practice-based, randomized, clinical trial of antihypertensive pharmacologic treatment and, in a specific subset, cholesterol-lowering, in 40,000 high-risk hypertensive patients, including at least 55% African-Americans (self-described "black"). The purpose of the antihypertensive trial component is to determine whether the combined incidence of fatal coronary heart disease (CHD) and non-fatal myocardial infarction differs between diuretic (chlorthalidone) treatment and three alternative antihypertensive pharmacologic treatments -- a calcium antagonist (amlodipine), an ACE inhibitor (lisinopril), and an alpha adrenergic blocker (doxazosin)*. Because of the established benefit of antihypertensive treatment in reduction of stroke, total morbidity and mortality from cardiovascular diseases, and all-causes mortality, the antihypertensive trial component will not include a placebo or no-treatment control group. The purpose of the cholesterol-lowering trial component is to determine whether lowering serum cholesterol in moderately hypercholesterolemic men and women aged 55 years and older with the 3-hydroxymethylglutaryl coenzyme A (HMG CoA) reductase inhibitor pravastatin will reduce all-cause mortality as compared to a control group receiving "usual care".

Secondary objectives of both trial components are to compare the effects of their respective treatment regimens on cardiovascular mortality, major morbidity, health costs, and health-related quality of life. Additional secondary objectives of the antihypertensive trial are to compare the effects of alternative treatments on all-cause mortality and on major hypertension-related morbidity such as incidence and regression of left ventricular hypertrophy and progressive renal dysfunction. Also the effect of the antihypertensive regimens on the aforementioned primary and secondary outcomes will be assess in key subgroups [over age 65, women, African-Americans, type II diabetics]. Also additional secondary objectives of the lipid-lowering trial are to assess the long-term safety of HMG CoA reductase inhibitors in men and women aged 55 years and above (particularly with regard to mortality from non-cardiovascular causes), the effect of lipid-lowering on cancer incidence and mortality, and the effect of lipid lowering on the combined incidence of fatal CHD and non-fatal myocardial infarction, especially in key subgroups [over age 65, women, African-Americans, type II diabetics]. Also, because this component of the trial will not be blinded, the incidence of myocardial infarction based on centrally coded changes in the biennial study ECG will be looked at as an end point. The mean duration of the trial is expected to be 6.0 years, ranging from 4.2 years (for the last patient entered) to 8 years (for the first patient entered).

To maximize statistical power for the primary hypotheses of the antihypertensive trial, i.e., the comparison of each alternative drug regimen to diuretic, 1.7 times as many patients will be assigned to its diuretic arm as to each of its other three arms (Table I.1). It is anticipated that half of ALLHAT participants will be randomized to both trial components and that half will be randomized only to the antihypertensive trial component.

---

* On January 24, 2000, a recommendation to discontinue the doxazosin arm of the antihypertensive trial was accepted by NHLBI. Please refer to JAMA.2000;283:1967-1975.

Table I.1: Design of ALLHAT

Cholesterol-Lowering Trial (2 Arms)	Antihypertensive Trial (4 Arms)*				Total
	Chlorthalidone	Amlodipine	Lisinopril	Doxazosin*	
Pravastatin	3,655	2,115	2,115	2,115	10,000
Usual Care	3,655	2,115	2,115	2,115	10,000
Not Eligible	7,310	4,230	4,230	4,230	20,000
Total	14,620	8,460	8,460	8,460	40,000

Because of the prohibitive cost of incorporating so many participants in a traditional clinical trial structure employing independently funded clinics, this trial will adopt an organizational model using patients recruited through approximately 600 primary care and hypertension practices, clinics, and health centers, each contributing an average of 67 patients. Department of Veterans Affairs (VA) hypertension clinics are expected to comprise approximately 70 of these clinical sites and to contribute about 20% of the study patients. Forms will be kept to a minimum, and few clinical procedures not performed for routine patient care will be required.

* On January 24, 2000, a recommendation to discontinue the doxazosin arm of the antihypertensive trial was accepted by NHLBI. Please refer to JAMA.2000;283:1967-1975.

II. Background and Rationale

A. Antihypertensive Trial

1) Hypertension and Coronary Heart Disease (CHD)

An estimated 50 million people in the U.S. have elevated blood pressure (systolic blood pressure, SBP, ∃ 140 mmHg and/or diastolic blood pressure, DBP, ∃ 90 mmHg) or are taking antihypertensive medication. In 1983, 23 million people were taking antihypertensive drugs [1]. The ambulatory care cost of treating hypertension in the U.S. was estimated at $7.5 billion for 1986 [2]. These costs in large part are determined by the costs of the agents used, which, given the number of patients treated, have substantial economic implications. All other factors remaining constant, the incremental cost of treating 25 million patients with a drug costing $100 per patient-year of therapy compared to one costing $500 per patient-year is $10 billion.

Despite the known etiologic relationship of hypertension to CHD, large-scale randomized clinical trials in mild to moderate hypertension (DBP 90-114 mmHg) in largely middle-aged subjects failed to demonstrate conclusively that antihypertensive drug treatment reduces the occurrence of CHD death or non-fatal myocardial infarction. The pooled results of nine such trials, employing primarily thiazide-like diuretics and involving over 43,000 subjects, suggest a 9% benefit, with 95% confidence limits consistent with a 19% benefit or a 1% adverse outcome [3]. This observed treatment effect compares with a maximum predicted effect on CHD of approximately 23% for an equivalent BP difference, as derived from epidemiologic data. In contrast, the observed beneficial effect on stroke in these trials, 36%, is almost exactly that which would be predicted from epidemiologic data [4]. A more recent overview [5] of 14 trials in participants with all levels of hypertension estimated a somewhat larger effect (14% benefit, 95% confidence interval, 4-22%). While there is reason to suspect that this may be an over-estimate of the benefit, these overviews do not include the strongly positive results of recent trials in the elderly, especially the Systolic Hypertension in the Elderly Program (SHEP), in which diuretic-based treatment reduced stroke incidence by 36% and major CHD events by 27% (95% confidence interval, 4-43%)[6].

One explanation for the failure of previous trials to demonstrate the expected degree of CHD reduction is that adverse effects of study drugs, particularly diuretics, may have offset the potential benefit of blood pressure reduction. These adverse effects include diuretic-induced hypokalemia, hypomagnesemia, hyperuricemia, hyperlipidemia, hyperglycemia, impaired insulin sensitivity, and probably increased ventricular ectopic activity [6,7]. The diuretic-induced increase in total cholesterol has been estimated to be approximately 4%, and in LDL cholesterol as much as 10% [7], though these effects may be attenuated with long-term treatment [5]. Such increases in blood lipids would be sufficient, if sustained, to offset a substantial portion of the CHD benefit from blood pressure reduction.

2) New Classes of Antihypertensive Agents

In the early 1980's two new classes of antihypertensive agents, the calcium antagonists and angiotensin-converting enzyme (ACE) inhibitors, were developed and licensed for use in chronic antihypertensive therapy. These agents tend to cost more than older agents such as diuretics and beta-blockers, and evidence that might justify their use despite the increased cost (such as greater efficacy or fewer side effects) is limited [2]. The fourth Joint National Committee on Detection, Evaluation, and Treatment of High Blood Pressure (JNC) recommended beta-blockers, calcium antagonists, ACE-inhibitors and diuretics as equally acceptable first-line therapy [9]. All four classes of drugs have been found to control diastolic blood pressure as single agents in 50% or more of patients with mild hypertension. The fifth JNC report [10] reconsidered the choice of initial therapy and recommended diuretics and beta-blockers as preferred agents. In addition, JNC-V added the alpha adrenergic blockers, as well as drugs with combined alpha and beta blocking actions, as alternative first-line treatments.

Of these drug classes, only beta-blockers have been compared directly to diuretics in large-scale, long-term clinical trials in hypertension. Three such trials completed in Europe in 1985-86 showed approximate equivalence of effects on morbidity and mortality in diuretic- and beta-blocker-based regimens. Pooled analysis of these trials yields a 6% (95% confidence interval, -10% to +22%) lower CHD mortality from beta-blockers [11]. These data are in contrast to the recent Medical Research Council (MRC) Trial in the Elderly, in which patients treated with a thiazide diuretic had significantly lower rates of CHD compared to beta-blocker (atenolol) treatment or placebo, both by about 45%.

Other data have shown that calcium-blockers may inhibit development of atherosclerotic lesions in rabbit models, but trial data on morbidity and mortality are conflicting. One trial of diltiazem in post-MI patients was interpreted as showing benefit in patients without low ejection fraction, but an overview pooling all post-MI trials with calcium-blockers reported a 6% (-4% to +18%) increase in mortality [12]. An update of this overview including three additional trials in patients with angina pectoris or myocardial infarction suggests more favorable results with agents that slow the heart rate compared to dihydropyridine calcium blockers [13]. Other trials in hypertensives have shown a decrease in left ventricular mass with calcium-blocker treatment [14].

Among ACE-inhibitors, at least three of the seven licensed drugs in this class have been reported to reduce left ventricular hypertrophy [15]. ACE inhibitors reduce mortality in both severe and less severe heart failure [16], and reduce morbidity, including CHD, in asymptomatic left ventricular dysfunction [17]. With regard to effects on atherosclerosis, Chobanian and colleagues have reported prevention of coronary lesions in the Watanabe rabbit model [18], perhaps due to effects on cellular proliferation in the vessel wall.

Several alpha-blockers have been shown to have moderate favorable effects on the lipid profile, particularly on LDL cholesterol [19,20]. A few studies have also found improvements in insulin resistance, an observation that may be especially relevant to patients with type II diabetes mellitus [21,22]. There is also some evidence that these

agents may reduce left ventricular hypertrophy and platelet aggregability and stimulate tissue plasminogen activator [23-27].

Only two long-term randomized trials have compared representatives of all of these drug classes: the one-year trial conducted by the VA Cooperative Study Group on Antihypertensive Agents [28], and the 4.4-year Treatment of Mild Hypertension Study (TOMHS) [19]. While these trials have reported some differences in BP control, side effects, quality of life, biochemical effects, and target-organ changes, these differences did not present a pattern that consistently favored some drugs and not others.

Data from a large variety of studies in humans and animal models thus suggest that newer drugs may be superior, equivalent or inferior to standard drugs in the treatment of hypertension. A report from the British Hypertension Society stated: "We thus conclude that beta-blockers or diuretics are equally acceptable first-line treatments.... Unfortunately... large-scale trials have not used newer antihypertensives such as calcium antagonists and angiotensin-converting enzyme inhibitors. There are therefore no comparable data for these widely used drugs, and we urgently need large-scale comparative trials to assess the role of these agents [29]." U.S. investigators have arrived at similar conclusions [30-32].

3) Importance of Comparing Antihypertensive Agents in African-Americans

Hypertension is considerably more common among African-Americans than Caucasians, and its sequelae are more frequent and severe. Prevalence of hypertension in the second National Health and Nutrition Examination Survey (NHANES II) was 51% in African-Americans aged 25-74 years compared to 40% in Caucasians [33]. Incidence of end-stage renal disease secondary to hypertension is nearly eight-fold higher in African-American than Caucasian hypertensives [34]. Risks of left ventricular hypertrophy, stroke and stroke death have all been reported to be greater among African-American hypertensives. Suggested explanations for these differences have included higher prevalence of co-existing illnesses such as diabetes among African-Americans, and decreased access to medical care.

Given that treatment effectively reduces hypertension-related cardiovascular morbidity and mortality, populations with decreased access to care might be expected to suffer disproportionately high rates of these complications. In such groups, particularly those of lower socioeconomic status (SES), cost of drug therapy may become the overriding consideration in selection and maintenance of treatment. The current trend toward use of more expensive agents is thus more likely to become a barrier to treatment in lower SES persons, who are disproportionately represented by African-Americans and who also bear a greater burden of hypertension-related diseases. If cheaper drugs such as diuretics are equally effective in preventing the complications of hypertension as are other available agents, low SES African-Americans are those most likely to benefit from this information. If cheaper drugs are less effective, low SES African-Americans are those most likely to suffer the consequences, since they will tend to receive cheaper drugs or none at all. In either situation, and given the relative lack of clinical trials information in African-Americans, they should be heavily represented in the current trial

so that the results will be directly applicable to them. For these reasons, the population for this trial will be at least 55% African-American.

B. Cholesterol-Lowering Trial

1) Cholesterol and Coronary Heart Disease

Circulating levels of cholesterol, specifically cholesterol associated with the low-density lipoprotein (LDL) fraction, have been established by observational epidemiologic studies, by metabolic, pathologic, and genetic studies in humans and selected animal models, and by randomized clinical trials, to be a major etiologic factor in coronary heart disease (CHD) [35]. The clinical trials that have demonstrated a reduction in CHD incidence from lowering LDL-cholesterol levels have been conducted primarily in middle-aged men with hypercholesterolemia or established CHD [36-41]. Experimental evidence for the efficacy of cholesterol lowering in older men is confined to the analysis of small subgroups of clinical trials, and is lacking for women of any age. This paucity of clinical trial data led the National Cholesterol Education Program's (NCEP) expert panel on detection, evaluation, and treatment of high blood cholesterol in adults to allow considerable room for physician judgment regarding the elderly in their 1987 guidelines [42].

The uncertainty in application of the NCEP guidelines to older men and to women is a matter of considerable consequence to the public health. While myocardial infarctions in middle-age are responsible for tragic losses of life and productivity, more than 80% of all CHD deaths in the U.S. occur after age 65 [43]. Women comprise more than 60% of the population above age 65, and the majority of CHD events in this age group are in women. In 1989, CHD was responsible for nearly 400,000 deaths and $14.4 billion in direct health care costs annually in this age group. This annual toll can be expected to increase over the next four decades as the aging of the "baby boom" generation and gains in longevity shift the age distribution of the U.S. population upward.

While the evidence relating elevated cholesterol levels to CHD is strong and consistent, clinical trials [44,45] have not demonstrated that cholesterol-lowering reduces total mortality. Moreover, observational studies [46] show a U-shaped relationship of cholesterol and mortality, with higher mortality rates for persons with cholesterol levels less than 160 mg/dl and greater than 240 mg/dl compared to those in the range 160-240 mg/dl. Deaths from hemorrhagic stroke, some cancers, and respiratory and digestive diseases contribute to the excess mortality at the lowest cholesterol levels [46]. In addition, reductions in CHD mortality from moderate (drug-induced) cholesterol lowering in randomized primary prevention trials in hypercholesterolemic patients have been offset by increases in trauma-related mortality [44]. These trials were not designed with adequate statistical power to address total mortality within the planned treatment period, though some have observed favorable post-trial trends [45,47]. Larger and more powerful clinical trials are needed to address this issue.

2) Cholesterol in Older Persons and African-Americans

While observational studies suggest that clinical trial results may be applicable to men with lower cholesterol levels and to women, the prognostic power of cholesterol levels in these studies diminishes with advancing age [48-51]. Pooled analyses of 21 male and 13 female cohorts suggest that the decrease in CHD risk associated with lower blood cholesterol levels above age 65, though statistically significant at least in men, is perhaps 60% of that below age 65, with considerable variation among studies [50]. However, this apparent diminution in relative risk associated with aging may be more than offset by the concomitant increase in absolute incidence of CHD deaths and myocardial infarctions [51]. Thus, the potential public health value of cholesterol lowering in seniors cannot be ignored. The unanswered question is the extent to which these "attributable" CHD events are actually <u>preventable</u> when cholesterol-lowering treatment is initiated at a relatively advanced age.

Although LDL-cholesterol levels are slightly lower and HDL-cholesterol levels are slightly higher in African-Americans than non-minority populations, hypercholesterolemia is still a substantial problem among African-Americans, particularly in women with obesity and diabetes. Approximately 23% of African-American men and women aged 25-74 years had high-risk serum cholesterol levels in 1976-80 [33], in whom reduction of cholesterol levels would be expected to reduce CHD mortality and morbidity. Prior cholesterol-lowering trials have included only a handful of African-American subjects. The assumption that cholesterol lowering will produce similar reductions in CHD in African-Americans and Caucasians is unproven.

3) Use of HMG CoA Reductase Inhibitors in Older Men and Women

Many previous clinical trials of cholesterol-lowering in middle-aged men have been weakened by the limited efficacy and acceptability of the drugs employed. However, lovastatin, the first of the HMG CoA reductase inhibitors, has been used increasingly widely since its approval by the FDA in September, 1987, and has been efficacious and well-tolerated by patients of all ages [52-55]. Two small angiographic trials have demonstrated a beneficial effect of lovastatin on coronary atherosclerosis [56,57]. Two additional HMG CoA reductase inhibitors, pravastatin and simvastatin, were approved by the FDA in late 1991; both have been used for several years in other countries. The FDA is currently considering approval of a fourth HMG CoA reductase inhibitor, fluvastatin.

Few serious side effects of these drugs have been observed to date. The absence of adverse lenticular changes in the 1990 report of the EXCEL study of lovastatin [55] prompted the FDA to remove its requirement for annual slit lamp exams from the product label. Significant but reversible asymptomatic elevations in serum transaminase levels have been seen in 1-2% of patients who have received these drugs. Myositis, in rare cases progressing to rhabdomyolysis and renal failure, is the most serious reported side effect of the HMG CoA reductase inhibitors and is potentiated by concomitant use of other potentially myotoxic drugs (immunosuppressive drugs, fibrates, etc.) and by impaired renal function. However, the incidence of myositis is quite low in the absence of these factors and does not significantly exceed placebo rates at low doses. Although

HMG CoA reductase inhibitors may potentiate the effects of anticoagulants, clinically significant interactions with the antihypertensive agents to be used in ALLHAT have not been reported.

Overall, because of their efficacy, ease of administration, low toxicity, and compatibility with most other drugs, the HMG CoA reductase inhibitors appear to be well-suited for use in older men and women. They also offer the opportunity to extend our knowledge of the benefits and safety of cholesterol lowering to cholesterol levels and degrees of reduction not previously addressed by large clinical trials.

A two-year pilot study for a trial of cholesterol lowering in seniors (Cholesterol Reduction in Seniors Program, CRISP) was initiated in July, 1990, at five clinical centers. Although it used a conventional trials model, with funded clinical sites instead of the office-based recruitment model planned for the current trial, the pilot study demonstrated the feasibility of recruiting older persons into a trial of cholesterol lowering [58]. A total of 431 men and women aged 65 and above were recruited into the pilot, surpassing the goal of 400 subjects within ten months. The pilot study cohort, which is 72% female with 25% minorities, was followed through June, 1992, to compare the compliance, safety, and efficacy of two alternative dosage regimens (20 mg and 40 mg, daily) of lovastatin versus placebo. Both dosages were well-tolerated, with 85-90% compliance after one year of treatment. The mean LDL reduction (28%) obtained with the 40 mg dosage only slightly exceeded that obtained with the 20 mg dosage (24%). Small increases in HDL cholesterol (7% and 9%) and decreases in triglycerides (4% and 10%) were also observed (respectively) for the 20 mg and 40 mg dosages.

III. Hypotheses and Study Power

A. Antihypertensive Trial Component

The primary hypotheses of this trial component are that the combined incidence of fatal CHD and nonfatal myocardial infarction will be lower in hypertensive patients receiving (1) a calcium antagonist (amlodipine), (2) an ACE inhibitor (lisinopril), or (3) an alpha adrenergic blocker (doxazosin)* as first-line therapy than in those in whom a similar degree of blood pressure control is achieved using a thiazide-like diuretic (chlorthalidone) as first-line therapy. These hypotheses will be tested in a population of men and women aged 55 years and older, all with at least one additional CHD risk factor besides hypertension, of whom at least 55% will be African-American. The statistical power to test these hypotheses is approximately 82.5%, based on the following assumptions:

1) Sample size of 40,000 (approximately 22,000 men and 18,000 women), allocated among four treatment groups as shown in Table I.1.
2) Six-year incidence of CHD events of 7.8% in the diuretic group. This rate, 1.35% per year, is based on the experience of Framingham and HDFP (adjusted downward by 33-50% for temporal trends and by 25% for the healthy volunteer effect) and is similar to the rate observed more recently in SHEP.
3) A 20% reduction in CHD event rate (before adjustment for non-compliance and losses to follow-up, which combine to produce a 16.3% effective reduction) in each of the three non-diuretic treatment arms compared to the diuretic arm.
4) Using a time-dependent Markov model, rates of crossover between each of the other study drugs and chlorthalidone and/or non-study medication are assumed to be 2.75% in each of the first three years and 6% over the last three years of follow-up (based on TOMHS). The probability of crossing over at least once during the study is assumed to be approximately 24%. It is assumed (conservatively) that the CHD risk associated with the non-study drugs is the same as for chlorthalidone.
5) CHD status will be undeterminable at the end of the study for 16.8% of patients (8.6% of person-years) due to competing risks (non-CHD death) or loss to follow-up, based on data from Framingham and HDFP (see Appendix I).
6) A 25% reduction in CHD event rates (before adjustment for non-compliance and losses to follow-up) among the 10,000 patients randomized to the active treatment arm of the cholesterol-lowering trial component.
7) A type I error $\alpha = 0.05$ (two-sided). This corresponds to a critical Z-score of 2.37 after adjustment for multiple comparisons.

Power estimates ranged from 77 to 86% for more pessimistic or optimistic assumptions of crossover (#4) and loss (#5) rates. Additional details regarding these calculations may be found in Appendix I.

* On January 24, 2000, a recommendation to discontinue the doxazosin arm of the antihypertensive trial was accepted by NHLBI. Please refer to JAMA.2000;283:1967-1975.

→ Secondary hypotheses pertaining to the effect of the following end points in patients randomized to receive amlodipine, lisinopril, or doxazosin* (relative to those receiving chlorthalidone) will also be assessed: (1) all-cause mortality, (2) combined coronary heart disease (CHD + revascularization procedures + hospitalized angina), (3) stroke, (4) combined cardiovascular disease (CHD + stroke + revascularization procedures + angina [hospitalized or treated] + CHF [hospitalized or treated] + peripheral arterial disease [hospitalized or outpatient revascularization procedure]), (5) left ventricular hypertrophy by ECG, (6) renal disease including the reciprocal slope of serum creatinine and end-stage renal disease (dialysis or transplant), (7) health-related quality of life, (8) fatal and non-fatal cancer by type, and (9) gastrointestinal bleeding.

B. Cholesterol-Lowering Trial Component: (See Protocol Addendum 1)

The primary hypothesis of this trial component is that mortality from all causes will be lower in the subset of hypertensive patients described above with LDL cholesterol levels between 120 and 189 mg/dl (between 100 and 129 mg/dl for those with known CHD) who are randomized to receive pravastatin plus a cholesterol-lowering diet than in those randomized to receive usual care. The statistical power to test this hypothesis is approximately 80%, based on the following assumptions:

1) Sample size of 20,000 (approximately 11,000 men and 9,000 women) allocated equally between pravastatin and usual care groups.

2) Six-year total mortality of 13.2% (2.35% per year) in the usual care group (based on data from Framingham, HDFP and SHEP (see Appendix I). Based on mortality data in a high-risk subgroup of SHEP participants selected for comparability to ALLHAT patients, it is estimated that 40% of deaths will be due to CHD, 16% due to other cardiovascular causes, and 44% due to noncardiovascular causes.

3) A 12.5% reduction in mortality in the pravastatin treatment arm. This estimate is based on the assumption that CHD mortality is reduced by 25%, that mortality from other cardiovascular diseases is reduced by 15%, and that other causes of mortality are unaffected. If it is assumed that full compliance to the drug regimen would reduce LDL cholesterol levels by 30% and that the mean LDL cholesterol level is 155 mg/dl at entry (Range: 120-189 mg/dl), a 25% reduction in CHD corresponds to a logistic regression coefficient of 0.0062. For comparison, the logistic coefficient relating total cholesterol to CHD mortality in 356,222 MRFIT (male) screenees was 0.0118 for the full age range (35-57 years) and 0.0086 for the 36,704 men in the oldest age group (55-57 years). Extrapolation from the MRFIT data suggests that the coefficient relating total cholesterol to CHD in ALLHAT might fall between 0.005 and 0.006.

→

* On January 24, 2000, a recommendation to discontinue the doxazosin arm of the antihypertensive trial was accepted by NHLBI. Please refer to JAMA.2000;283:1967-1975.

4) A "dropout" rate (from pravastatin treatment to no treatment) of 5% in Year 1, and 2.5% in all subsequent years, and a "drop-in" rate (from no treatment to pravastatin or a similar drug) of 2% per year. These rates are projected to yield 15.3% of pravastatin patients off treatment and 10.6% of usual care patients on treatment at the end of 6 years. Note that the power of the cholesterol-lowering trial, which (unlike the antihypertensive trial) focuses on the generic effects of cholesterol-lowering, rather than the effects of a specific drug or drug class, is not diminished by "crossovers" from pravastatin to other regimens that produce equivalent lipid changes.

5) No losses to follow-up.

6) A 10% reduction in mortality rate in each of the three non-diuretic treatment arms of the anti-hypertensive trial component.

7) A type I error $\alpha = 0.05$ (two-sided), corresponding to a critical Z-score of 1.96.

Power was also calculated for a range of annual mortality rates (2.2 to 2.5%) and for a somewhat more pessimistic set of assumptions regarding compliance (leading to 17.8% and 12.9% prevalence of drop-outs and drop-ins, respectively, at the end of six years). Statistical power estimates ranged from 75 to 82% under these assumptions (see Appendix I).

Secondary hypotheses pertaining to reduction of the following end points in patients randomized to receive pravastatin (relative to those receiving usual care) will also be assessed: (1) the combined incidence of CHD death and nonfatal myocardial infarction, especially in certain subgroups like African-Americans, patients over age 65 (the original CRISP hypothesis), type II diabetics, and women, (2) changes in the biennial study ECG indicative of myocardial infarction, (3) cause-specific mortality, (4) total and site-specific cancer incidence, and (5) health-related quality of life.

The power of this study to address the effect of pravastatin on the combined incidence of CHD death and nonfatal myocardial infarction is estimated to be 97% overall and close to 80% in any subgroup containing 10,000 patients with risk characteristics similar to the overall cohort (see Appendix 1). However, the objectivity of the clinical diagnosis of nonfatal myocardial infarction is potentially compromised by the fact that the treating physicians will not be blinded as to whether their patients have received pravastatin or usual care. To guard against bias, the incidence of nonfatal myocardial infarction will also be assessed by changes in the biennial study ECG, the evaluation of which will be performed by coders who are unaware of the patient's treatment assignment. A pravastatin-usual care difference in clinical events will be given credence only if confirmed by a qualitatively similar difference in the ECG end point.

IV. Eligibility and Exclusions

A. Antihypertensive Trial

1. Age/sex: Men and women aged 55 years and older.
2. Medical history: History of documented hypertension.
3. Seated blood pressure:

Eligibility is based on the patient's current treatment status and on the average of two seated blood pressure measurements at each of two visits (Table IV.1):

(a) Patients whose blood pressure has been controlled (the majority of blood pressure measurements # 160/100 mmHg) with one or two antihypertensive drugs for at least two months are eligible. Patients who are taking three or more antihypertensive drugs at subtherapeutic doses or in ineffective combinations, and who are felt likely to be controllable on the ALLHAT protocol, can enter the trial at the discretion of the principal investigator or his/her designee.

(b) For untreated patients, a diagnosis of hypertension must first be established. (The JNC V criteria for diagnosing hypertension are included in the Manual of Operations.) Patients who are untreated or who have been treated for less than two months must meet the minimal as well as the maximal blood pressure criteria shown in Table IV.1. To qualify for entry, the lower SBP *or* DBP limit and *both* upper limits must be met on two occasions at least one day apart. Patients who do not meet the blood pressure entry criteria at Visit 1 or Visit 2 may be re-evaluated for blood pressure eligibility at a later time.

Table IV.1. Blood Pressure Eligibility Criteria

Status at Visit 1 and Visit 2		Lower Limit[1] (mmHg)		Upper Limit[2] (mmHg)	
		SBP	DBP	SBP	DBP
On 1-2 Drugs Used for Hypertension ∃ 2 Months	Visit 1	---	---	160	100
	Visit 2	---	---	180	110
On Drugs For < 2 Months or Currently Untreated	Visit 1 & Visit 2	140	90	180	110

[1] SBP *or* DBP lower limit must be met at Visit 1 and Visit 2
[2] SBP *and* DBP upper limit must be met at Visit 1 and Visit 2

4. At least one of the following:

   a. One or more of the following manifestations of atherosclerotic cardiovascular disease:
      (i) Old or age-indeterminate myocardial infarction or stroke (>6 months),
      (ii) History of revascularization procedure (ever),
      (iii) Documented atherosclerotic cardiovascular disease. This includes, but is not limited to: coronary, peripheral vascular, aortic, or carotid stenosis as documented by angiography, Doppler studies, or diminished ankle-arm index; ischemic heart disease as documented by electrocardiography (e.g., ST-T wave changes), echocardiography, or radionuclide imaging; history of intermittent claudication; or history of transient ischemic attack. (A more complete list of manifestations of atherosclerotic cardiovascular disease is provided in the Manual of Operations.)

   b. Type II diabetes mellitus [plasma glucose >140 mg/dl (fasting) or 200 mg/dl (non-fasting) and/or on insulin or oral hypoglycemic agent] (in the past 2 years),

   c. HDL-cholesterol <35 mg/dl (on any 2 determinations *within past 5 years*)

   d. Left ventricular hypertrophy (one of the following) on any ECG within the past 2 years:

! R amplitude in V_5 or V_6 > 26 mm.

! R amplitude in V_5 or V_6 plus S amplitude in V_1 > 35 mm.

! R amplitude in aVL > 12 mm.

! R amplitude in Lead I > 15 mm.

! R amplitude in Leads II or III, or aVF > 20 mm.

! R amplitude in Lead I plus S amplitude in Lead III > 25 mm.

! R amplitude in aVL plus S amplitude in V_3 > 28 mm for men or > 22 mm for women

! Computerized ECG machine documented LVH

For visual LVH reading, QRS amplitudes are measured in the ***second to last complete normal beat*** of the lead.

   e. Left ventricular hypertrophy on any echocardiogram (within the past 2 years) based on 25 mm or more combined wall (ventricular septum plus posterior wall) thickness.

   f. Current cigarette smoking (any cigarettes smoked in past 30 days).

5. Exclusions:

   a. Symptomatic MI or stroke within past six months.

   b. Hospitalized or treated symptomatic congestive heart failure and/or ejection fraction < 35%, if known.

c. Angina pectoris within past 6 months. (This implies actual chest pain. If patient has history of angina and no chest pain, even if he/she is on antianginal drugs, then this exclusion ***does not*** apply. See exclusion 5e.)

d. Known renal insufficiency (serum creatinine ∃ 2 mg/dl).

e. Patients requiring diuretics, calcium antagonists, ACE inhibitors, or alpha adrenergic blockers for reasons other than hypertension. (If a patient is on a calcium-channel blocker for angina, he/she may be switched to a beta-blocker for this indication if it is deemed safe to do so. This exclusion criterion would not then be applicable.)

f. Patients requiring more than two antihypertensive drugs to achieve satisfactory blood pressure control (SBP # 160 mmHg and DBP # 100 mmHg). Patients who are taking three or more antihypertensive drugs at subtherapeutic doses or in ineffective combinations, and who are felt likely to be controllable on the ALLHAT protocol, can enter the trial at the discretion of the principal investigator or his/her designee. See IV A 3(a).

g. Sensitivity or contraindications to any of first-line study medications.

h. Factors suggesting a low likelihood of compliance with protocol, such as dementia, history of alcohol or drug abuse within past six months, plans to move or travel extensively, or history of unreliability in keeping appointments or taking prescribed drugs.

i. Diseases, such as non-curable malignancy, likely to lead to non-cardiovascular death over the course of the study.

j. Blood pressure over 180 mmHg systolic or over 110 mmHg diastolic on two separate readings during step-down of antihypertensive medications.

k. Current participation in another clinical trial

B. Cholesterol-Lowering Trial

1. Eligible and enrolled in antihypertensive trial.

2. Fasting LDL Cholesterol: 120 to 189 mg/dl (100 to 129 mg/dl for patients with known CHD). These cutpoints, which correspond roughly to the 30th and 90th percentiles in men and the 25th and 85th percentiles in women in the ALLHAT age range, are projected to include approximately 60% (24,000) of ALLHAT participants from whom 20,000 would remain after refusals and other exclusions.

3. Fasting triglyceride level below 350 mg/dl.

4. Additional Exclusions:

a. Current use of prescribed lipid-lowering agents or large doses (∃ 500 mg/day) of non-prescription niacin . Eligible patients must be off lipid-lowering drugs at least two months and off probucol for more than one year at the time of Visit 2.

b. Contraindications to HMG CoA reductase inhibitors (e.g., significant liver disease, ongoing immunosuppressive therapy, known allergy or intolerance to the study drug).

c. Known untreated secondary cause of hyperlipidemia (e.g., hypothyroidism, nephrotic syndrome).

d. ALT > 2.0 x upper limit of normal.

V. Recruitment

Recruitment for ALLHAT will rely primarily on chart review to identify patients who are potentially eligible for the antihypertensive or both trial components. Data needed to make the definitive determination of eligibility for the antihypertensive trial component will be obtained in a series of pre-randomization visits, which will take place over a period generally not exceeding two months. The number and frequency of those visits will depend on the complexity of the patient's pre-study regimen, the blood pressure response to step-down from that regimen, and the patient's suitability for and interest in the cholesterol-lowering trial component. Because only patients who have been randomized to the antihypertensive trial component will be considered for randomization to the cholesterol-lowering trial component, randomization to the latter will not take place until the first post-randomization (4 week) visit for the antihypertensive trial. The steps leading from identification of these various categories of potential ALLHAT candidates to randomization in one or both study components are described below.

Chart Review:

At each clinical site, patients who might potentially be suitable for the antihypertensive component of ALLHAT and the subset of such patients who might also be eligible for the cholesterol-lowering component of ALLHAT will be identified by chart review. Information on blood pressure and antihypertensive treatment, LDL (or total if LDL is unavailable) cholesterol levels and cholesterol-lowering diet and/or drugs, other relevant medical history, and a sense of the patient's reliability and compliance with previously prescribed treatments should be reviewed for conformity with the study eligibility requirements (Chapter IV) before his/her initial study visit.

A. Antihypertensive Trial Component

Visit 1: Preliminary Determination of Eligibility and Interest

The objective of Visit 1 is to assess eligibility for and interest in ALLHAT and to begin withdrawing the patient from any existing antihypertensive medications. It is anticipated that the majority of treated hypertensives will have been identified by chart review, and that much of the pertinent information (age, risk factor status, number of antihypertensive drugs, etc.) will already be known. The investigator will be required to complete a one-page questionnaire to document that all the preliminary inclusion and exclusion criteria (Chapter IV) have been met. Any documentation not attainable by chart review or not available within the past two years (ECG to assess the presence of left ventricular hypertrophy, fasting glucose level for diabetes, total cholesterol (TC) level to assess lipid eligibility), or within the last five years for the two determinations of HDL, will be considered part of the patient's routine medical management and will not be specifically reimbursed by the study.

Visit 1 will consist primarily of obtaining the first entry blood pressure, answering the patient's questions about the study, and obtaining the patient's informed consent to begin the step-down if necessary from pre-study antihypertensive drugs. Recommendations for antihypertensive drug withdrawal are included below. If a patient's antihypertensive medications

can be safely switched without tapering, the participant may move directly to Visit 2. Visits 1 and 2 should be separated by at least one day, but need not be consecutive visits.

For untreated patients, a diagnosis of hypertension according to JNC V criteria needs to be established. Once this is done, those patients whose SBP ∃ 140 mmHg and/or DBP ∃ 90 mmHg and whose SBP and DBP do not exceed 180 and 110 mmHg, respectively (see Table IV.1), may also be considered for entry into ALLHAT. For new patients found to have elevated blood pressure at their initial visit to the clinical site, this initial visit may serve as ALLHAT Visit 1 provided that any additional evaluations needed to determine study eligibility are performed (at no cost to the study). The subsequent course of such patients is simplified by elimination of the need for a step-down from a pre-study drug regimen.

Step-down visits:

Not all patients on antihypertensive medications will require step-down visits. In general, the following categories of antihypertensive drugs can usually be stopped without tapering the dose:

1. Diuretics: The patient should be informed to contact the physician if he/she develops marked edema and/or significant increase in dyspnea (shortness of breath, either at night or on exertion).
2. Reserpine.
3. Angiotensin converting enzyme (ACE) inhibitors.
4. Calcium channel blockers.
5. Vasodilators (e.g., hydralazine).
6. Alpha$_1$-blockers (e.g., prazosin).

The following categories need to be tapered if the patient is taking more than the usual starting doses:

1. Central agonists (e.g., oral clonidine or methyldopa) - taper over 1-2 weeks
2. Beta-adrenergic blockers (e.g., propranolol or atenolol) - taper over 2 weeks

Post-myocardial infarction patients receiving beta-blockers for prophylaxis need not discontinue therapy. There may be occasional circumstances where, in the physician's judgment, closer monitoring or a longer period of withdrawal is preferred. Extra care should be taken in tapering antihypertensive drugs in those patients with cardiovascular disease.

Patients whose blood pressure exceeds 180 mmHg systolic or 110 mmHg diastolic should return within a few days for a repeat blood pressure measurement. If the blood pressure is still above 180/110, the patient should not be randomized into the antihypertensive trial.

Visit 2: Randomization:

Patients who have met all ALLHAT eligibility criteria and in the judgment of the investigator can safely discontinue all prior antihypertensive drugs and be randomized to one of the four ALLHAT treatment arms shall, after giving their informed consent, be entered into the study at Visit 2. This visit will generally take place between 1 day and 12 weeks after Visit 1, depending on the length of time required to step down from pre-study medications. Patients initially taking no drugs or well-controlled on one drug may be randomized soon after Visit 1, while other patients may require a longer step-down process (generally less than three months) before they can complete Visit 2. More prolonged step-downs are discouraged (though not prohibited), since many patients who cannot quickly be withdrawn from their pre-study regimens may also be more difficult to maintain on a simple regimen during the trial.

The investigator will telephone the Clinical Trials Center regarding each patient who meets all eligibility requirements at Visit 2, including a signed consent form. The Clinical Trials Center will review the eligibility and exclusion criteria and will assign that patient a study identification number and a bottle number corresponding to (1) chlorthalidone, (2) amlodipine, → (3) lisinopril, or (4) doxazosin*. The treatment assignment will be masked from both the practitioner and patient. A resting ECG, serum glucose, serum potassium and creatinine, fasting lipid profile and ALT should be obtained at this visit for all patients who are randomized. Each randomized patient will be issued an appropriate supply of his/her starting dose of the assigned study drug and will be instructed to return for the first dosage titration (Visit 3) four weeks later (see Section VI).

All randomized patients will be given appropriate hygienic advice (sodium and alcohol restriction, smoking cessation, exercise, caloric restriction if overweight) with reinforcement as needed during the trial.

B. Cholesterol-Lowering Trial Component

Visit 1: Preliminary Determination of Eligibility and Interest

Patients who have satisfied all Visit 1 eligibility requirements for the antihypertensive trial component (see above) and/or have consented to begin step-down from pre-study antihypertensive drugs should also be informed of the cholesterol-lowering trial component of ALLHAT. Those who indicate their possible interest in this component and have not been treated with lipid lowering drugs (including 500 mg or more per day of over-the counter niacin) during the two months preceding Visit 1 shall be considered as potential candidates for this trial. Patients who have taken probucol within one year preceding Visit 1 are also ineligible for this component of ALLHAT.

→ * On January 24, 2000, a recommendation to discontinue the doxazosin arm of the antihypertensive trial was accepted by NHLBI. Please refer to JAMA.2000;283:1967-1975.

Visit 2: Fasting LDL Determination (Randomization to Antihypertensive Trial Component)

A fasting lipid battery (total cholesterol, triglycerides, HDL-cholesterol, calculated LDL-cholesterol) and serum ALT will be obtained for patients who: are eligible and randomized into the antihypertensive trial component of ALLHAT. Patients who have not fasted at least 9 hours (12 hours optimum) should have their blood draw rescheduled within a week of Visit 2. If rescheduling for fasting lipids is required, this will be considered part of Visit 2.

Visit 3 or 4: Randomization

Patients with fasting LDL-C between 120 and 189 mg/dl (between 100 and 129 mg/dl for patients with known CHD) and fasting TG # 350 mg/dl at Visit 2 will be informed by telephone of their eligibility for the cholesterol-lowering trial component and told to come in fasting for Visit 3. If they sign the Informed Consent to participate in this ALLHAT component at Visit 3, the investigator will phone the Clinical Trials Center, review the eligibility and exclusion criteria for the lipid-lowering component, and receive a random assignment for the patient to either pravastatin or usual care. Each patient randomized to receive pravastatin will be issued an appropriate supply of 20 mg tablets and instructed to take two each evening. Patients assigned to usual care will not be prescribed any lipid-lowering medication by ALLHAT. Patients assigned to usual care as well as those assigned to pravastatin will be advised to follow the NCEP Step I diet (<30% of calories from fat, <10% of calories from saturated fat, <300 mg cholesterol per day). A fasting lipoprotein profile will be obtained at this visit as a baseline for each randomized participant in this trial component.

Maintenance of Racial Composition of ALLHAT:

Before their practices are selected as clinical sites for ALLHAT, potential study investigators will be asked to indicate the approximate proportion of African-American patients they expect to recruit into the study. Clinical sites will be selected to produce an overall study population of at least 55% African-Americans and will be monitored by the Clinical Trials Center throughout the study to assure that their performance matches their expectations. If the overall proportion of African-Americans appears to be falling significantly short of 55%, the Steering Committee may implement remedial measures such as temporarily freezing recruitment of non-African-American patients at some or all existing clinical sites or adding new clinical sites to correct the shortfall.

Table V.1: Schematic Summary of Entry of Patients into Two ALLHAT Components:

Visit #	Months from Visit 2	Purpose: Antihypertensive Trial	Purpose: Cholesterol-Lowering Trial
-	-6.0 to -0.2	Chart Review	
1*	-2.0 to -0.2	Assess Eligibility and Interest	
1a,b,c*	-1.5,-1.0,-0.5 (as needed)	Step down from pre-study antihypertensive drugs	
2	0	Randomization, diet/lifestyle counselling	Fasting LP profile**, ALT
3*	1	Routine data collection Dosage titration if needed	Randomization, Fasting LP Profile**, NCEP Step 1 diet
4	3	Routine data collection Dosage titration if needed	Dosage titration if needed ALT, TC
5,6,7	6,9,12 (more often if needed*)	Routine data collection Dosage titration if needed	Routine data collection Dosage titration if needed
8,9,10,..	Every 4 months	Routine data collection	Routine data collection

*** Separate reimbursements are not provided for these visits. At Visit 3 (1 month), reimbursement is provided if the patient is randomized to the lipid-lowering component. For visits past 1 month, reimbursement is not provided for visits other than those at 3, 6, 9 and 12 months during the first year, and every 4 months thereafter.**

****Total cholesterol, triglyceride, and HDL cholesterol levels. LDL calculated by Friedewald formula.**

Post-randomization visits are shaded.

VI. Antihypertensive Intervention

The blood pressure goal in all four arms* will be <90 mmHg diastolic and <140 mmHg systolic.[1] The number and dose of study drugs prescribed in pursuit of these goals will be influenced by patient tolerance and clinical judgment, particularly in use of greater than two-drug regimens. With rare exceptions, treatment should be intensified for patients with BP levels ∃ 160 mm Hg systolic and/or ∃ 100 mm Hg diastolic, even if low doses of drugs from the same classes as the blinded Step 1 drugs must be added.

The therapeutic goal is to achieve blood pressure control on the lowest possible dosage of the first-line drug. The addition of second-line (open label) drugs should be reserved for those in whom the maximal dosage level of the first-line drug is insufficient.

Each of the four* first-line drugs will be administered once daily in the morning. The following dosage levels will be available for each drug:

Table VI.1. First-Line (Blinded) Antihypertensive Drugs

Step 1 Agent	Initial Dose	Dose 1	Dose 2	Dose 3
Chlorthalidone	12.5	12.5	12.5	25
Amlodipine	2.5	2.5	5	10
Lisinopril	10	10	20	40
Doxazosin*	1	2	4	8

Sources of the four Step 1 agents are: chlorthalidone: Ogden Bioservices, Inc., Rockville, Maryland; amlodipine: Pfizer, Inc., New York, New York; and lisinopril: Zeneca Pharmaceuticals Group, Wilmington, Delaware; and doxazosin*: Pfizer, Inc., New York, New York.

The identity of the drug will be masked at each dosage level , but the identity of the dosage level will not be masked. The initial dosage level will be used only during the first week after randomization to minimize the potential side effects of doxazosin*. (For the other three drugs, the initial dose and Step 1 dosages are identical.) The Step 1 dosage level should be initiated at the end of the week. A clinic visit is not required.

All patients will be re-evaluated at least at 1 month for dose titration if needed (Visit 3) and at 3 months (Visit 4). Study medication will be initiated at the initial dose and patients should typically return at one-month intervals for any necessary increase in dosage until both the

---

* On January 24, 2000, a recommendation to discontinue the doxazosin arm of the antihypertensive trial was accepted by NHLBI. Please refer to JAMA.2000;283:1967-1975.

[1] These goals follow the Fifth Joint National Committee recommendations [10]. The JNC set the systolic goal lower than that used in SHEP, which was between 140 and 159 mm Hg with a mean attained value of 142 mm Hg, because of the known strong epidemiologic relationship of systolic pressure with CVD mortality [59].

systolic and diastolic goal pressures are reached. If the initial dose of the blinded drug is not tolerated, it should be discontinued. When the symptom clears, or after a month, if the symptom is not a known serious adverse effect of one of the blinded study drugs, another trial of the study drug should be attempted. If the symptom recurs and is believed to be related to the blinded drug, the drug should be discontinued and one of the open-label second-line antihypertensive drug drugs should be initiated. Consideration should be given to subsequently adding the blinded study drug, as side effects from one drug are often ameliorated by another drug (e.g., a beta-blocker may prevent side effects of an alpha blocker).

If the investigator or the patient is concerned about the blood pressure level, titration visits may occur more often than monthly, but this is discouraged for titration of the blinded drug. If a dose level of blinded drug is not tolerated, the dosage level should be reduced. An attempt should be made to keep the patient on some dose of blinded drug. If the blood pressure goals are not achieved on the maximal tolerated dose of blinded drug, one of the open-label antihypertensive drugs should be added and titrated as needed and tolerated (see below).

→ For patients in any of the four* treatment arms who are unable to attain satisfactory blood pressure control on the maximum available dosage of their first-line drug that they can tolerate, a choice of the following second- and third-line drugs and dosages will be provided in open-label form for addition to (not substitution for) the first-line drug:

Table VI.2. Second- and Third-Line (Open Label) Antihypertensive Drugs

Step 2 and Step 3 Agents	Dose 1	Dose 2	Dose 3
Reserpine	0.05 qd or 0.1qod	0.1 qd	0.2 qd
Clonidine (oral)	0.1 bid	0.2 bid	0.3 bid
Atenolol	25 qd	50 qd	100 qd
Hydralazine (Third-line)	25 bid	50 bid	100 bid

The choice of second-line drug(s) will be at the discretion of the treating study investigator/ physician. Since the study investigators will be blinded to the identity of the first-line drug to which each patient is assigned, it is assumed that the frequency of use of each of the second-line drugs will be similar among the four treatment arms. Although in special cases, investigators may choose to prescribe second-line antihypertensive drugs other than those provided by the study, thiazide diuretics, calcium antagonists, ACE inhibitors, and adrenergic
→ blockers* should be avoided for this purpose unless maximum tolerated doses of a 3-step (3 study drugs) regimen have been tried. Only in those circumstances, or when another clinical indication besides uncontrollable hypertension (e.g., severe angina or congestive heart failure)

→

* On January 24, 2000, a recommendation to discontinue the doxazosin arm of the antihypertensive trial was accepted by NHLBI. Please refer to JAMA.2000;283:1967-1975.

mandates a drug of one of these classes, may such a drug be used as a last resort. If one of the specific classes of Step 1 drugs (diuretic, calcium antagonist, ACE inhibitor, alpha-blocker*) must be used, the doses of added open-label drugs from one of the Step 1 classes should not exceed 2 the maximal recommended doses in JNC V. (These dosage recommendations will be listed in the ALLHAT Manual of Operations.) In most such cases, it will not be necessary to unmask the patient or the investigator as to the identity of the treatment arm to which that patient was assigned.

ALLHAT patients will typically be seen at 4-week intervals until a reasonably stable regimen with satisfactory blood pressure control has been achieved. After this is accomplished, routine clinic visits shall take place at three-month intervals during the first year and at four-month intervals thereafter. However, after the blood pressure has been effectively controlled for at least one year and at least 3 visits, it may be possible to attempt to reduce any second-line and/or third-line therapy (but not the blinded study drug) in a deliberate, slow and progressive manner.

The following data will be collected:

1. Blood pressure at each visit.

2. Electrocardiogram at randomization (Visit 2) and every two years thereafter.

3. Serum potassium at randomization, one month, at one year, two years, four years and six years

4. Fasting lipid profile**,** serum glucose, creatinine and storage of blood for future analyses of genetic factors for CHD at randomization.

5. Serum creatinine at one month and at one year.

6. Fasting total cholesterol**,** serum glucose and creatinine every other year after randomization.

7. Compliance with study medications at every visit, estimated by self-report and graduated cylinder measurements of unused pills.

8. Interim study end points, hospitalizations and doctors' visits at each visit.

9. Quality of life at entry, and every other year thereafter.

Investigators may wish to locally monitor serum creatinine more frequently in patients with serum creatinine 1.4 mg/dl or higher.

---

* On January 24, 2000, a recommendation to discontinue the doxazosin arm of the antihypertensive trial was accepted by NHLBI. Please refer to JAMA.2000;283:1967-1975.

Physician-investigators will routinely receive reports of all central laboratory determinations except serum potassium level, which will be masked after Visit 2. However, the Clinical Trials Center will notify the clinical site promptly of any serum potassium values below 3.5 meq/l or above 5.5 meq/l . If the level is between 3.2 and 3.5 meq/l, it will be rechecked locally at the next scheduled visit. If the level is below 3.2 meq/l or above 5.5 meq/l, the participant should be recalled immediately to have his/her potassium level rechecked locally. Oral potassium supplementation will be prescribed for any patient whose potassium level is below 3.5 meq/l on recheck.

The oral potassium supplementation provided by ALLHAT will be a slow-release formulation in 8 meq tablets. The suggested starting dosage is 5 tablets (40 meq) once per day, which may be raised as needed to 12 tablets (96 meq) per day. Adequacy of potassium supplementation will be monitored locally at the clinical site at patient expense. A 20 meq tablet (KDur) will be available for selected participants with serum potassium persistently below 3.2 meq/l.

Practitioners will be reimbursed a fixed fee for each patient randomized to each component of the trial and for each subsequent study visit completed. This fee is expected to cover the costs of all the data collection (step-down and titration visits, questionnaires, blood drawing, ECG recording) specified above. The fee does not include the cost of laboratory work and ECG coding, which will be performed by central facilities and paid for directly by the Clinical Trials Center, or the costs for documenting study end points, for which there will be separate reimbursement.

VII. Cholesterol-Lowering Intervention

The cholesterol lowering component of ALLHAT will employ a simple, randomized comparison of the HMG CoA reductase inhibitor pravastatin versus usual care in a subset of patients participating in the antihypertensive component of the study. The starting dosage of pravastatin will be 40 mg, taken in the evening. All participants in this ALLHAT component will receive instruction in the Step I diet recommended by the NCEP upon randomization into the study. Randomization to this trial component will take place four weeks after randomization into the antihypertensive component of ALLHAT, at the first titration visit (Visit 3).

Follow-up visits for the cholesterol-lowering component will correspond to routine follow-up visits of the antihypertensive trial. In addition to the data collected for the antihypertensive component as described in Chapter VI, the following post-randomization data will be obtained for patients in the cholesterol-lowering component:

1. Serum ALT at the visit after randomization to the lipid component. The central laboratory will not perform ALT determinations on patients assigned to usual care or those who have discontinued taking pravastatin for reasons other than ALT elevation.

2. Fasting lipid profile (total, HDL, and estimated LDL cholesterol and triglyceride) at annual visits for a fixed 10% cohort of the patients assigned to receive pravastatin. Fasting lipid profiles will also be done at the second, fourth, and sixth annual ALLHAT visits for a fixed 5% cohort of patients assigned to usual care. Ultracentrifugal determinations of LDL cholesterol will be performed at Visit 3 (but not subsequently) when triglyceride levels exceed 400 mg/dl.

3. On the remaining 90% of the patients assigned to receive pravastatin, total cholesterol will be measured annually. (The remaining 95% of usual care patients, like the ALLHAT patients who participate only in the antihypertensive trial, will have only biennial determinations of total cholesterol.) In these patients, LDL cholesterol will be estimated for monitoring purposes by subtracting the fall (from baseline level) in total cholesterol (Visit 3 - Visit X) from the baseline (Average of Visits 2 and 3) level of LDL cholesterol. For example, a patient with TC=225 mg/dl and LDL=150 mg/dl at baseline, whose follow-up TC=280 mg/dl, will be estimated to have LDL=205 mg/dl (150 + 280 - 225). This estimate (which will be calculated centrally) assumes that any change in total cholesterol is due entirely to a change in the LDL component.

4. Compliance with pravastatin, estimated by self-report and graduated cylinder measurements of unused pills.

The Central Laboratory will routinely provide ALT and lipid values to study investigators in a timely manner. ALT levels exceeding three times the upper limit of normal will be flagged as "alert" values. When ALT is in the alert range, blood should be redrawn and sent to the central laboratory for repeat measurement. If the alert value is confirmed and is not explained by other factors, the investigator should consider stopping the study medication. (See "Stopping Study Medications", Section XI.) The patient may subsequently be rechallenged with a 10 mg dose after the ALT returns to pre-treatment levels. If no further difficulties are

encountered after four months at reduced dosage, the dosage may be increased to 20 mg, and then after another four months to 40 mg, with repeat ALT testing after each dosage increase. ALLHAT investigators are also expected to reduce or, if necessary, to discontinue the study medication in the presence of other medically significant side effects that are judged to be attributable to pravastatin and to rechallenge (if judged appropriate) with a reduced dosage when the side effects subside.

A more restrictive diet and/or low-dose resin (e.g., 8 g/day of cholestyramine) may be prescribed at the physician's discretion for patients who fail to attain a satisfactory cholesterol response despite good compliance with the maximal dosage of pravastatin and the study diet; however, the study will not provide cholesterol-lowering drugs other than pravastatin. If a low-dose resin is prescribed along with pravastatin, the resin dose should be separated from the pravastatin dose by at least two hours.

Practitioners will be reimbursed a fixed additional fee for each patient randomized to this component of ALLHAT. This fee is expected to cover the costs of all additional data collection (questionnaires, blood drawing) specified above for this component of the trial. The fee does not include the cost of the additional laboratory work, which will performed by central facilities and paid for directly by the Clinical Trials Center.

VIII. Laboratory Measurements

Routine laboratory measurements will be performed according to the schedule in Table VIII.1. A CDC-standardized Central Laboratory will perform the lipid and clinical chemistry determinations, and an ECG coding center will review and assign Minnesota codes to ECGs. Other data needed to assess the eligibility of patients for ALLHAT are expected to have been collected locally in the course of routine medical management and will not be reimbursed by the study. The study will provide for a blood chemistry battery, including serum lipids, for a resting ECG at baseline, and for storage of blood for future analyses of genetic factors for CHD .

The Central Laboratory will set up a system and provide materials to the clinical sites for the packaging and mailing of serum samples to them. The laboratory results will be sent promptly to the Clinical Trials Center, and (with the exception of potassium data, which will generally be blinded) to the clinical sites. Any results requiring action on the part of the practitioners will be flagged. The Central Laboratory will promptly notify the clinical sites concerning patients whose serum potassium levels are below 3.5 or above 5.5 meq/dl, or if the serum potassium level increases at least 1 meq/dl to a level above 5.0 meq/dl, so that the treating physician can take appropriate corrective action (see Chapter VI).

The ECG coding center will receive copies of all baseline (Visit 2) ECGs and will review all routine post-randomization study ECGs (comparing with Visit 2) for the presence of new myocardial infarction and for the appearance or disappearance of left ventricular hypertrophy (LVH). They will also review ECGs obtained from a 10% sample of hospitalizations in which the diagnosis of myocardial infarction was made or considered, using prior study ECGs for comparison. The ECG coding center will promptly notify the clinical sites concerning patients whose ECGs indicate acute myocardial infarction or third degree AV block.

Table VIII.1: Laboratory Measurements at Each Visit

Visit #	Follow-Up, months	All ALLHAT Participants	Cholesterol-Lowering Component	
			Pravastatin	Usual Care
**2**	**0**	**K, glucose, creatinine, LP, ALT, ECG, storage of blood for future genetic analyses of CHD risk**		
**3**	**1**	K, creatinine		
4	3		ALT, TC	
5	6			
6	9			
7	12	K, creatinine	TC, LP (10% subset)	
8	16			
9	20			
10	24	K, glucose, creatinine, TC, ECG	LP (10% subset)	LP (5% subset)
11	28			
12	32			
13	36		TC, LP (10% subset)	
14	40			
15	44			
16	48	K, glucose, creatinine, TC, ECG	LP (10% subset)	LP (5% subset)
17	52			
18	56			
19	60		TC, LP (10% subset)	
20	64			
21	68			
22	72	K, glucose, creatinine, TC, ECG	LP (10% subset)	LP (5% subset)
23	76			
24	80			
25	84		TC, LP (10% subset)	
26	88			
27	92			
28	96	K, glucose, creatinine, TC, ECG	LP (10% subset)	LP (5% subset)

***Monthly dosage titration Visits will be scheduled as needed between visits 3&4, 4&5, 5&6, etc. until effective stable antihypertensive regimen is attained.**

Abbreviations: K=Serum potassium, ALT=Serum alanine aminotransferase, ECG=Resting 12-lead electrocardiogram, LP =Fasting lipid profile, TC=Serum total cholesterol, HDL-C=Serum high-density lipoprotein cholesterol, LDL-C=Serum low-density lipoprotein cholesterol, TG=Serum triglyceride. Fasting lipid profile includes TC, HDL-C, and TG determinations. LDL-C is estimated by the Friedewald formula unless triglyceride levels exceed 400 mg/dl; in such cases ultracentrifugal determinations of LDL cholesterol will be performed.

ALT values will also be determined in patients restarted at a lower dose of pravastatin after earlier discontinuation due to abnormal liver function tests.

IX. Outcome Measurements

Occurrences of study endpoints will be documented by a checklist completed by the study physician at each follow-up visit and supplemented by interim reporting as needed. These diagnoses will be supported by copies of death certificates, discharge summaries and face sheets as described below. The following outcome measures will be obtained and tabulated over the course of the study:

A. Death (documented by death certificate).

The underlying cause of death will be classified by the physician-investigator at the clinical site as due to (1) Coronary Heart Disease, (2) Other Cardiovascular Disease, (3) Neoplastic Disease, (4) Other Medical Causes, or (5) Non-Medical Causes. A National Death Index (NDI) Search will be performed near the end of the study to identify and document deaths that may have occurred among patients who are lost to follow-up. Because of the time lag inherent in the NDI, a private tracing service will also be utilized for selected participants. Physicians will also be asked to report cause of death on the study endpoint form.

B. Cardiovascular End Points

1) Myocardial infarction (documented by hospital discharge summary or face sheet or by biennial study ECG), including suspected myocardial infarction with thrombolytic therapy.

2) Stroke (documented by hospital discharge summary or face sheet).

3) Congestive heart failure

   a) Hospitalized or procedure (documented by hospital discharge summary or face sheet)

   b) Not hospitalized but treated (documented by check box on end point questionnaire)

4) Angina pectoris

   a) Hospitalized or procedure (i) with or (ii) without a revascularization procedure (documented by hospital discharge summary or face sheet)

   b) Not hospitalized but treated (documented by check box on end point questionnaire)

5) Peripheral arterial disease

a) Hospitalized or procedure (i) with or (ii) without a revascularization procedure (documented by hospital discharge summary or face sheet) or outpatient revascularization procedure (documented by procedure sheet)

b) Treated medically as outpatient (documented by check box on end point questionnaire)

6) Left ventricular hypertrophy (documented by biennial study ECG)

The LVH inclusion criteria are based on specific ECG critera as listed in IV.A.4.d and will be interpreted at the clinical site. ECGs will be re-read centrally to assign Minnesota Codes. The outcome criteria for LVH are based on the Minnesota Code. The Minnesota Coding Center will use Codes 3-1 or 3-3 to identify prevalent LVH. These amplitude criteria sets are generally considered "probable ECG-LVH", but when combined with any 4-3 or more severe 4-code, or 5-3 or more severe 5-code, it is considered "definite ECG-LVH".

Minnesota Code 3-1: R amplitude > 26 mm in either V_5 or V_6 or R amplitude > 20 in any of leads I, II, III, aVF, or R amplitude > 12 mm in lead aVL.

Minnesota Code 3-3: R amplitude in V_5 or V_6 plus S amplitude in V_1 > 35 mm or R amplitude > 15 mm but # 20 mm in Lead I.

The Coding Center will also document incident ECG-LVH and progression/regression of ECG-LVH using serial ECG comparison.

C. Other End Points

1) Decreased renal function (documented by reciprocal slope of serum creatinine level versus time -- continuous measure)
2) End stage renal disease (initiation of chronic dialysis, kidney transplant)

a) Hospitalized or procedure (documented by hospital discharge summary or face sheet)

b) Treated as outpatient (documented by check box on end point questionnaire)

3) Cancer -- Site and Type

a) Hospitalized or procedure (documented by hospital discharge summary or face sheet)

b) Treated as outpatient (documented by check box on end point questionnaire)

4) Nonfatal accidents and attempted suicides

a) Hospitalized or procedure (documented by hospital discharge summary or face sheet)

b) Treated as outpatient (documented by check box on end point questionnaire)

5) Gastrointestinal bleeding

a) Assessed through data from the Health Care Finance Administration and the Department of Veterans Affairs

6) Quality of life -- A generic categorical measure of health status will be used to assess health related quality of life.

7) Medical care utilization-- Utilization data will be collected by interview. Costs will be assigned to each unit of utilization (hospitalization, office visits, procedures, etc.) based on its DRG. In addition, one question will be asked to ascertain quality of life on a continuous scale in order to determine quality-adjusted life years. For a 10% sample of patients over age 65, these interview data will be cross-checked versus Medicare records.

The study investigators will be required to complete and submit to the Clinical Trials Center a short end points questionnaire for each occurrence of a study endpoint identified at or between regular visits. For each end point involving a death or hospitalization, the investigator will also obtain and submit a copy of the death certificate or hospital discharge summary or face sheet upon which the diagnosis is based. For a random (10%) subset of hospitalized (fatal and nonfatal) myocardial infarctions and strokes, the Clinical Trials Center will request the more detailed information as described in Appendix I so that the in-hospital ECGs and enzyme levels (for myocardial infarctions), and neurologists' reports and CT and/or MRI reports (for strokes) can be evaluated by the study end points committee and the accuracy of the discharge diagnoses (versus the definitions in Appendix I) can be assessed.

X. Study Organization

Overview:

ALLHAT will employ an organizational structure that differs markedly from the usual NHLBI-supported clinical trial. The trial will be performed by a large number (600) of practicing physician-investigators who will be compensated on a per capita basis for each patient seen according to a fixed payment schedule. Approximately 20% of study patients are expected to be recruited by Department of Veterans Affairs (VA) hypertension clinics. The Clinical Trials Center, in addition to its conventional data handling and monitoring responsibilities, will be responsible for identifying and paying these physician-investigators, hiring regional coordinators to monitor recruitment and compliance, and for awarding and supervising subcontracts for a central laboratory and an ECG coding center. A Steering Committee will be selected for their expertise in the relevant subject areas. A detailed description of the nature and role of the study components is given below.

Program Office

The Program Office, located in the NHLBI, Division of Epidemiology and Clinical Applications (DECA) and Division of Heart and Vascular Diseases (DHVD), will award and monitor the contract that provides funding for the study, set up the agreements to fund the VA clinics, and hold the IND for the study. The Director, NHLBI, will appoint the Data and Safety Monitoring Board (DSMB) and the Chair and Vice-chair of the Steering Committee. With the concurrence of the Director, NHLBI, the Program Office will appoint the Steering Committee and any other committees deemed necessary to advise the NHLBI on issues pertaining to the progress or results of the study.

Clinical Trials Center:

The Clinical Trials Center will have primary responsibility for identifying suitable medical practices to participate in ALLHAT, paying them according to a fixed fee schedule for each patient randomized and study form completed, and for editing, storing, and analyzing data generated by the study. Its investigators and staff will have a central role in designing the data collection system and in monitoring data quality. Specific Clinical Trials Center responsibilities include:

1) Developing, preparing, and distributing the study protocol, data forms, and Manual of Operations and Procedures.

2) Appointing and paying practicing physicians to provide clinical sites for conduct of the study.

3) Appointing and paying regional coordinators (see below).

4) Obtaining Institutional Review Board (OPRR) approval for uncovered practices.

5) Maintaining files of annual financial disclosure statements for the Steering Committee members to identify potential conflicts of interest.

6) Subcontracting for a central laboratory and an ECG coding center to provide timely and standardized measurements needed by the study (see Chapter VIII).

7) Subcontracting for a Drug Distribution Center to receive, bottle, label, and distribute study medications to the clinic sites.

8) Monitoring the performance of study components and providing timely summary reports to the Program Office and to the Steering Committee.

9) During recruitment, monitoring the proportion of African-Americans at each clinical site and recommending appropriate corrective action if the overall proportion for the study as a whole appears to be falling significantly short of the 55% target.

10) Providing detailed and up-to-date statistical reports of study progress to the Data and Safety Monitoring Board (DSMB) at their semi-annual meetings (see below).

11) Maintaining a referral network for study participants who move to a new geographic region and are unable to continue to see their original study physician.

12) Providing logistical support (as needed) and minutes for study meetings.

13) Coordinating and supervising end point verification activities.

14) Initiating searches through the National Death Index to establish the vital status of patients who are lost to follow-up at intervals recommended by the DSMB and Steering Committee (see below).

15) Preparing study manuscripts in collaboration with the Steering Committee.

Clinical Sites:

These will consist of 600 separate medical practices, designated by the Clinical Trials Center to conduct the study. It is expected that some practices (particularly the VA clinics, HMOs, and large group practices) will provide larger numbers of patients and that some practices may contribute fewer than 100 patients. The proportion of African-Americans is also expected to vary among clinics, but will be monitored closely to ensure that the target of 55% overall is met (see Chapter V).

Each clinical site is expected to be under the supervision of a physician identified as responsible for the conduct of ALLHAT. However, study forms may be completed by a physician's assistant or nurse practitioner or other designated qualified personnel, consistent with the internal organization of that medical practice. At each site, one support staff member must be designated as chiefly responsible for protocol implementation; this person will participate in central training and annual meetings. Payment for each patient randomized to each ALLHAT component and for each study visit completed will be made by the Clinical Trials Center upon receipt of the relevant completed, correct and signed study form.

Regional Coordinators:

Regional coordinators will be physicians with expertise in hypertension and cholesterol lowering treatment, who will handle routine protocol questions for approximately 50 clinical sites apiece. Under direction of the Clinical Trials Center, they will assist in solving problems related to quality control, protocol adherence, recruitment and retention for the sites assigned to them. Physician coordinators will be supported by a nursing coordinator and may opt to participate as clinical sites as well. All participating VA hypertension clinics will be supervised by a single coordinator.

Drug Distribution Center

A Drug Distribution Center will be established by the Clinical Trials Center to (1) receive, package and distribute all pharmaceuticals required for the two ALLHAT components, (2) implement a system of masking so that the four first-line antihypertensive agents cannot be distringuished from each other by the study investigators or their patients (the second-line antihypertensive drugs will not be masked), and (3) provide appropriate supplies of all study medications to the clinical sites on a timely basis.

Steering Committee:

The Steering Committee will be appointed by the NHLBI to provide expert advice on the study protocol and on all subsequent decisions pertaining to the design and conduct of the study that do not require access to blinded data, and the eventual analysis and publication of the study results. Its voting members will be the NHLBI Project Officer, the principal investigator of the Clinical Trials Center, the Regional Coordinators, and 7-9 experts selected for their expertise and experience in the treatment of hypertension and/or hypercholesterolemia and in key clinical trials issues such as recruitment and adherence. Each Steering Committee member will be required to submit an annual financial disclosure statement to the Clinical Trials Center and to divest themselves of any stock holdings or retainer-type consultant positions in pharmaceutical and other companies that have a direct financial interest in the outcome of the study. The Steering Committee will meet once per year (more frequently during protocol development).

An Executive Committee will be instituted to oversee trial operations between Steering Committee meetings. Composition of the Executive Committee will include the Chair and Vice-Chair of the Steering Committee and representatives of the Program Office, the Clinical Trials Center, and the Department of Veterans Affairs. Reporting to the Executive Committee will be the following subcommittees: Eligibility and Medical Care, Operations, Publications and Ancillary Studies, Scientific and Educational Program, and Endpoints. Each of the subcommittees will have representation from the Program Office, Clinical Trials Center, and Steering Committee to oversee aspects of the trial that require frequent attention and/or special expertise, such as recruitment, adherence, quality control, blood pressure and lipid intervention, laboratory methods, endpoint verification, ancillary studies, publications, and the annual program for the investigators' meetings.

Protocol Review Committee:

The Protocol Review Committee will be responsible for advising the NHLBI regarding the initial approval of the study protocol. Its members and chair will be appointed by the Director, NHLBI, and will consist of at least seven experts who are not otherwise affiliated with the study. It will meet in Bethesda when the study protocol has been completed. The meeting will be attended by the principal investigator (and designated staff) of the Clinical Trials Center and the Chair and Vice-Chair of the Steering Committee who will make presentations and answer questions regarding the protocol, and by Program Office staff.

Data and Safety Monitoring Board (DSMB):

The DSMB will be responsible for monitoring all aspects of the study, including those that require access to blinded data. The DSMB and its chair will be appointed by the Director, NHLBI, and will consist of at least seven experts who are not otherwise affiliated with the study. It is likely that the roster of the DSMB members may be largely or even entirely derived from the Protocol Review Committee, which will complete its mission as the DSMB is formed.

The DSMB will meet at least semi-annually. The principal investigator of the Clinical Trials Center and designated Clinical Trials Center staff will attend these meetings (but will not have a vote) and will be responsible for preparing and presenting up-to-date statistical reports on the progress of the study. These reports will include data on recruitment, randomization, adherence, blood pressure levels, plasma lipoproteins, adverse drug responses, and study end points, as well as statistical tests and special analyses requested by the DSMB. The Project Director (who will serve as the DSMB's Executive Secretary), Project Officer and designated NHLBI staff and the Chair and Vice-chair of the Steering Committee will also participate in these meetings in ex officio capacities.

During the active recruitment phase, the DSMB will monitor the progress of recruitment (particularly of African-American patients) and the random allocation of participants to the various treatment arms and may recommend modifications in (or termination of) one or both study components if the study design goals are not being met. The DSMB will recommend when to end the active recruitment phase of the study. The approval of the DSMB will also be required for any significant changes in the protocol recommended by the Steering Committee during the course of the study. All votes will be decided by a simple majority.

At any time during the study, the DSMB may recommend discontinuation of any of the treatment arms of either study component on any of the following grounds:

1) compelling evidence from this or another study of an adverse effect of the study treatment(s) that is sufficient to override any potential benefit on CHD and preclude its further use in the target population;

2) compelling evidence from this or another study of a significant beneficial effect of the study treatment(s), such that its continued denial to the other study groups is ethically untenable;

3) a very low probability of successfully addressing the study hypotheses within a feasible time frame, because of inadequate recruitment, compliance, drug response, event rate, etc.

The DSMB may convene an Executive Session at any time. DSMB members, the Project Director and the Project Officer will attend these sessions.

The Director, NHLBI will make the final decision on whether or not to accept the DSMB's recommendation to discontinue any component of the study.

XI. Data Management

Report distribution

At least five types of reports will be generated:

1) Recruitment reports: These are expected to be generated at least weekly, by clinic and region and for the antihypertensive trial and the lipid-lowering trial. These will be distributed to the Project Office, Steering Committee and Regional Coordinators.

2) Other routine monitoring reports include data on visit and medication adherence, quality control and study endpoint documentation. These will be generated at least monthly by clinic and region, and will be distributed to the Project Office, Steering Committee and Regional Coordinators.

3) Reports for clinic use include randomization verification reports, visit schedules and reminders, endpoint documentation reports, and limited cross-forms edits. These will be generated no more often than monthly. Visit schedules are generated as participants are randomized and include all visit windows and expected special procedures for the duration of the study. Reports and appropriate listings will be sent to the clinics, and summary reports will be sent to the Project Office, Steering Committee and Regional Coordinators.

4) Steering Committee reports will be generated for annual meetings and will be similar to routine recruitment and monitoring reports.

5) Data and Safety Monitoring Board reports will include recruitment and monitoring data by treatment group for both the antihypertensive trial and the lipid-lowering trial. They will also include summary reports of data from the central laboratory and biennial and event ECG data, as well as study endpoints by treatment group.

Quality Control

All clinical sites will be required to attend one of three regional training sessions. These training sessions will include orientation to the study protocol, blood pressure measurement training and certification, orientation to the ECG procedures, and training in completion and transfer of study forms.

Periodic refresher training will be held in conjunction with regularly scheduled Steering Committee meetings. These refresher sessions may include a review of correct blood pressure measurement procedures or any problem that may be identified through review of routine monitoring activities.

All forms will be reviewed for completeness and accuracy at the Clinical Trials Center prior to data entry. Any problems identified will be resolved by telephone or facsimile transmission with the clinical site. Study forms will then be double data entered. Limited cross-forms edits will be performed to identify missing forms and procedures.

Unblinding

In some special circumstances (e.g., a medical emergency), a patient's assigned treatment group may be revealed. The Regional Coordinators will be the first line of advice in the decision of whether to break the blind or not. If the Regional Coordinator cannot be reached, the investigator should try to contact any of the other Regional Coordinators, or Dr. Davis or Dr. Goff at the Clinical Trials Center, or Dr. Payne or Dr. Cutler at NHLBI to discuss the relevant medical issues. If medically appropriate, an effort will be made to maintain the blinding of the patient and the clinical center investigator. If the regional coordinator agrees, or if the investigator is insistent, the investigator should contact the Clinical Trials Center to determine the unblinded treatment assignment. If there is an emergency and the Clinical Trials Center cannot be contacted, the investigator will reveal the unblinded treatment assignment by contacting a central unblinding facility.

When breaking the blind is determined to be necessary, the circumstances will be documented on a 1-page form by the clinic investigator. The form will be forwarded to the Clinical Trials Center and data entered onto the masterfile as a permanent part of the patient's study record.

Stopping Study Medications

If an investigator believes it is necessary to withdraw a patient from study treatment because of an adverse effect, other symptoms, of physician's judgment, it may not be necessary to break the blind on the patient. The Regional Coordinators will be the first line of advice in the decision of whether to withdraw the patient from study treatment. If the Regional Coordinator agrees, or if the investigator is insistent, the investigator should contact the Clinical Trials Center to inform them that this action is being taken and the reasons for it.

Reimbursements

The clinical sites will be reimbursed on a capitation basis for each randomization to the antihypertensive trial, randomization to the lipid-lowering trial, each protocol-required follow-up visit, and each completed study endpoint. These payments will be made monthly from the Clinical Trials Center. In order for a reimbursement to be authorized, a study form must be received at the Clinical Trials Center, all questions regarding that form must be resolved, and the form must be entered onto the study database. In the case of study endpoints, reimbursements will be in several parts made separately for the study form itself, the death certificate or discharge summary, and for additional documentation for the 10% sample for verification.

Data Analysis

The primary endpoint of the antihypertensive component of ALLHAT is fatal plus nonfatal CHD. The primary response variable is time from randomization to development of this event. The log rank test [60] will be used to compare each of the non-diuretic treament groups to the diuretic one. For the secondary endpoints of all-cause mortality, stroke, combined coronary (CHD + revascularization procedures + hospitalized angina) and cardiovascular (CHD + stroke + revascularization procedures + angina [hospitalized or treated] + CHF [hospitalized or treated] + peripheral arterial disease [hospitalized or outpatient revascularization procedure]) outcomes, and end-stage renal disease, the log rank test will also be used. The log rank test will also be used to test if there are treatment differences in the following subgroups for the outcome of fatal and non-fatal CHD - 1) men and women, 2) ∃ 65 years and < 65 years, 3) African-Americans, and 4) diabetics and non-diabetics. For the outcomes of LVH by ECG, and health-related quality of life, comparison of proportions will be used to see if there are differences in the treatment groups. For the outcome of renal disease, the inverse of the slope of creatinine will be calculated for each participant. The weighted average of the participants' inverses in each treatment group will be calculated and these averages will be compared across groups using the longitudinal models of Laird and Ware [61].

The primary endpoint of the lipid-lowering component of ALLHAT is all-cause mortality. The primary response variable is time from randomization to death. The log rank test will be used to compare the group assigned to lipid-lowering therapy to the group assigned to no treatment. For the secondary endpoints of fatal and nonfatal CHD, fatal and nonfatal cancer and cause-specific mortality, the log rank test will also be used. The log rank test will also be used to test if there are treatment differences in the following subgroups for the outcome of fatal and non-fatal CHD - 1) men and women, 2) ∃ 65 years and < 65 years, 3) African-Americans, and 4) diabetics and non-diabetics. For the outcomes of MI by ECG, and health-related quality of life, comparison of proportions will be used to see if there are differences in the treatment groups.

Interim Monitoring and Analysis (See Protocol Addendum 2)

Interim monitoring will focus on patient intake - overall and within clinical center, center adherence to protocol, baseline comparability of treatment groups, sample size assumptions with regard to event rates, crossover rates, competing risk and lost to follow-up, adverse effects data, and effect of treatment on the primary and secondary study outcomes. Interim analyses will coincide with the meetings of the Data and Safety Monitoring Board (DSMB).

We recommend the DSMB use stochastic curtailment for monitoring treatment differences in both the hypertension and the lipid-lowering studies [62,63]. By this method, termination in favor of the alternative hypothesis (H_a) may be considered if the conditional probability of rejecting the null hypothesis (H_o) at the scheduled end of the study given the current data and assuming H_o is true, is greater than or equal to some pre-specified value γ_o. Alternatively, termination in favor of the null hypothesis would be considered if the conditional probability of rejecting H_o under the design specified alternative hypothesis is less than some pre-specified value γ_a. With this procedure, the type I error is inflated slightly above α (depending on the number of looks at the data, the timing of the looks, and the value of γ_o) and the type II error is slightly inflated above β (again, depending on the number of looks at the data,

the timing of the looks, and the value of γ_a). The choice of the γ's will be determined by the DSMB.

These monitoring procedures are suggested as guides for the complex and subjective decisions the DSMB must make when considering to continue or terminate randomization and/or follow-up at each of its meetings.

Hypertension Trial

In this trial, we have three* comparisons of interest - diuretic compared to angiotensin converting enzyme inhibitor, diuretic compared to calcium channel blocker, and diuretic compared to alpha blocker*. Each comparison would have its own monitoring guidleine under the Dunnet procedure with α=0.019. Figure 1 shows the 80% stochastic curtailment boundaries for each of the comparisons. The looks will depend on the information time (the number of recorded CHD events divided by the expected number of CHD events) at the calendar time of the Data and Safety Monitoring Board meetings.

Hypertension trial

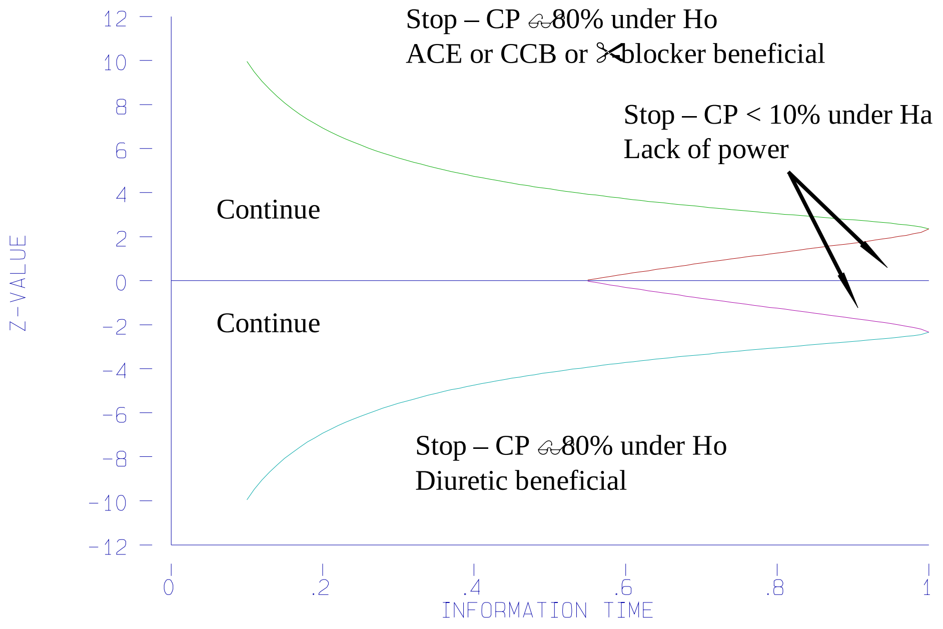

CP = Conditional power

Figure 1. Monitoring boundaries for the hypertension trial - ACE (or CCB or alpha-blocker*) vs. diuretic

* On January 24, 2000, a recommendation to discontinue the doxazosin arm of the antihypertensive trial was accepted by NHLBI. Please refer to JAMA.2000;283:1967-1975.

We would also wish to use a rule for stopping because of lack of power to show an effect. Here we also propose to use stochastic curtailment or conditional power. We would consider stopping if the conditional power under the proposed alternative hypothesis is less than 10%. Figure 1 also displays this conditional power boundary.

Total mortality will also be monitored in the hypertension trial.

Lipid Lowering Trial

For this trial we would propose similar procedures as to that of the hypertension trial with the exception that there is only one comparison (all-cause mortality) with an α=0.05. Figure 2 depicts the monitoring boundaries.

Lipid-lowering trial

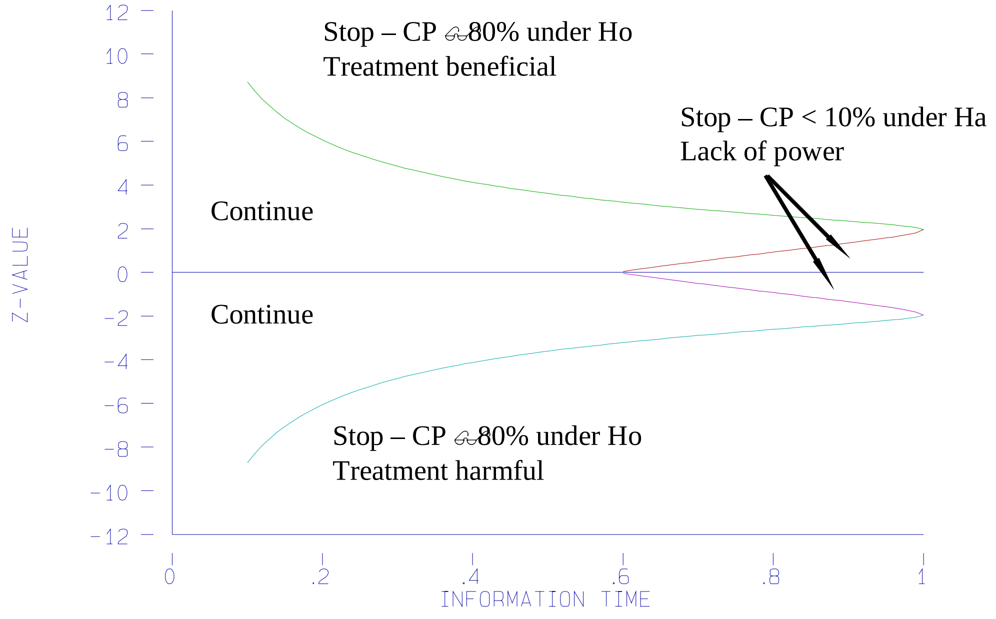

CP = Conditional power

Figure 2. Monitoring boundaries for the lipid-lowering trial.

XII. Vanguard Phase

The initial six months of the study will comprise a vanguard phase for the full-scale trial. Twenty practices will be selected to carry out this vanguard phase with the goal of randomizing six hundred (of the full complement of 40,000) patients. Objectives of the vanguard phase include:

1. To determine the feasibility of recruitment and follow-up of out-patient hypertensive subjects in office-based practices and hypertension clinics;

2. To determine the proportion of antihypertensive trial subjects eligible and willing to participate in the cholesterol-lowering trial;

3. To optimize strategies for recruitment of at least 55% African-American participants;

4. To develop methods for maximizing adherence to antihypertensive and cholesterol-lowering medication regimens in out-patient hypertensive subjects;

5. To optimize strategies for retention of out-patient hypertensive subjects in office-based practices;

6. To develop methods for standardized endpoint ascertainment in office-based practices and hypertension clinics; and

7. To assess and optimize the effectiveness of various operational strategies, such as drug distribution, Institutional Review Board approval, training, and activities involving the Regional Coordinators.

If the vanguard phase establishes the basic feasibility of this protocol, any necessary modifications will be made and additional practices will be recruited as needed to meet the recruitment goals of the study.

XIII. References

1. Hypertension prevalence and the status of awareness, treatment, and control in the United States: Final report of the subcommittee on definition and prevalence of the 1984 Joint National Committee. Hypertension 1985; 7:457-468.
2. Stason WB. Cost and quality trade-offs in the treatment of hypertension. Hypertension 1989; 13 (Suppl I): I-145--I-148.
3. MacMahon SW, Cutler JA, Furberg CD, et al. The effects of drug treatment for hypertension on morbidity and mortality from cardiovascular disease: A review of randomized controlled trials. Prog Cardiovasc Dis 1986; XXIX (3, Suppl 1):99-119.
4. MacMahon S, Cutler JA, Stamler J. Antihypertensive drug treatment: Potential, expected, and observed effects on vascular disease. Hypertension 1990; 335:827-838.
5. Collins R, Peto R, MacMahon S, et al. Blood pressure, stroke, and coronary heart disease. Part 2: Short-term reductions in blood pressure: Overview of randomized drug trials in their epidemiological context. Lancet 1990; 335:827-838.
6. SHEP Cooperative Research Group. Prevention of stroke by antihypertensive drug treatment in older persons with isolated systolic hypertension: Final results of the Systolic Hypertension in the Elderly Program (SHEP). JAMA 1991;265:3255-3264.
7. Cohen JD, Neaton JD, Prineas RJ, Daniels KA for the MRFIT Research Group. Diuretics, serum potassium, and ventricular arrhythmias in the Multiple Risk Factor Intervention Trial. Am J Cardiol 1987; 60:548-554.
8. Ferrari P, Rosman J, Weidman P. Antihypertensive agents, serum lipoproteins, and glucose metabolism. Am J Cardiol 1991; 67:26B-35B.
9. 1988 Report of the Joint National Committee on Detection, Evaluation and Treatment of High Blood Pressure. Arch Int Med 1988; 148:1023-1038.
10. National High Blood Pressure Education Program. The Fifth Report of the Joint National Committee on Detection, Evaluation and Treatment of High Blood Pressure. Arch Int Med 1993; 153:154-183.
11. Furberg CD, Cutler JA. Diuretic agents versus beta-blockers: Comparisons of effects on mortality, stroke, and coronary events. Hypertension 1989; 13(Suppl I): I-57--I-61.
12. Held PH, Yusuf S, Furberg CD. Calcium channel blockers in acute myocardial infarction and unstable angina: An overview. Brit Med J 1989; 299:1187-1192.
13. Yusuf S, Held P, Furberg C. Update of effects of calcium antagonists in myocardial infarction or angina in light of the second Danish Verapamil Infarction Trial (DAVIT-II) and other recent studies. Am J Cardiol 1991; 67:1295-1297.
14. Messerli FH, Oren S, Grossman E. Left ventricular hypertrophy and antihypertensive therapy. Drugs 1988; 35(Suppl 5): 27-33.
15. Yusuf S, Wittes J, Friedman L. Overview of results of randomized clinical trials in heart disease. I. Treatments following myocardial infarction. JAMA 1988; 260:2088-2093.

16. The SOLVD Investigators. Effect of enalapril on survival in patients with reduced left ventricular ejection fractions and congestive heart failure. N Engl J Med 1991; 325:293-302.

17. The SOLVD Investigators. Effect of enalapril on mortality and the development of heart failure in asymptomatic patients with reduced left ventricular ejection fractions. N Engl J Med 1992; 327:685-691.

18. Chobanian AV, Haudenschild CC, Nickerson C, et al. Anti-atherogenic effect of captopril in the Watanabe heritable hyperlipidemic rabbit. Hypertension 1990; 15:327-331.

19. Neaton JD, Grimm RH Jr, Prineas RJ, et al. for the Treatment of Mild Hypertension Study Research Group. Treatment of Mild Hypertension Study: final results. JAMA 1993;270:713-724.

20. Pool JL. Effects of doxazosin on serum lipids: A review of the clinical data and molecular basis for altered lipid metabolism. Am Heart J 1991;121:251-259.

21. Lithell H. Effects of antihypertensive drugs on insulin, glucose and lipid metabolism. Diabetes Care 1991;14:203-209.

22. Sowers J et al. The effects of doxazosin on blood pressure, glucose tolerance and insulin metabolism in patients with mild-to-moderate hypertension and type II diabetes mellitus. Presented at the Inter-American Society of Hypertension, April 1993.

23. Grimm et al. Alpha blockade and diuretic treatment of hypertension: A double-blind randomized comparison of doxazosin and hydrochlorothiazide. Presented at the American Society of Hypertension, May 1992.

24. DeQuattro et al. Neurogenic factors in primary hypertension: An alpha-receptor blocker - doxazosin - lowers stress and resting blood pressure. Presented at the American Heart Association 65th Scientific Sessions, November 1992.

25. Hernandez RH et al. Evidence of an antiplatelet aggregation in normotensive subjects and patients with hypertension: An in-vitro study. Am Heart J 1991;121:389-394.

26. Hernandez RH et al. Evidence of an antiplatelet aggregation action of doxazosin in patients with hypertension: An ex-vivo study. Am Heart J 1991;121:395-401.

27. Jansson JH. Effects of doxazosin and atentolol on the fibrinolytic system in patients with hypertension and elevated serum cholesterol. Eur J Clin Pharmacol 1991;40:321-326.

28. Materson BJ, Reda DJ, Cushman WC, et al. for the VA Cooperative Study Group on Antihypertensive Agents. Single-drug therapy for hypertension in men: a comparison of six antihypertensive agents with placebo. N Engl J Med 1993;328:914-921.

29. Report of the British Hypertension Society Working Party. Treating mild hypertension: Agreement from the large trials. Br Med J 1989; 298:694-698.

30. Zusman RM. Alternatives to traditional antihypertensive therapy. Hypertension 1986; 8:837-842.

31. O'Kelly BF, Massie BM, Tubau JF, Szlachcic J. Coronary morbidity and mortality, pre-existing silent coronary artery disease, and mild hypertension. Annals Intern Med 1989; 110:1017-1026.

32. National Heart, Lung, and Blood Institute Report of the Task Force on Research in Hypertension. U.S. Department of Health and Human Services, Public Health Service, National Institutes of Health, May, 1991.

33. National Center for Health Statistics. Health, United States, 1988. DHHS Publication No. (PHS) 89-1232. Public Health Service. Washington, D.C. U.S. Government Printing Office, March, 1989, p.99.

34. Whittle JC, Whelton PK, Seidler AJ, Klag MJ. Does racial variation in risk factors explain black-white differences in the incidence of hypertensive end-stage renal disease? Arch Intern Med 1991; 151:1359-1364.

35. NIH Consensus Conference. Lowering blood cholesterol to prevent heart disease. JAMA 1985; 253:2080-2086.

36. Lipid Research Clinics Investigators. The Lipid Research Clinics Coronary Primary Prevention Trial Results. I. Reduction in incidence of coronary heart disease. JAMA 1984; 251:351-364.

37. Frick MH, Elo O, Heinonen O, et al. Helsinki Heart Study: Primary-prevention trial with gemfibrozil in middle-aged men with dyslipidemia. Safety of treatment, changes in risk factors, and incidence of coronary heart disease. N Engl J Med 1987; 317:1237-1245.

38. Holme I. An analysis of randomized trials evaluating the effect of cholesterol reduction on total mortality and coronary heart disease incidence. Circulation 1990; 82:1916-1924.

39. Ravnskov U. Cholesterol lowering trials in coronary heart disease: frequency of citation and outcome. Br Med J 1992; 305:15-19.

40. Rossouw JE, Lewis B, Rifkind BR. The value of lowering cholesterol after myocardial infarction. N Engl J Med 1990; 323:1112-1119.

41. Buchwald H, Varco RL, Mills JP, et al. Effect of partial ileal bypass surgery on morbidity and mortality from coronary heart disease in patients with hypercholesterolemia. Report of the Program on the Surgical Control of Hypercholesterolemia (POSCH). N Engl J Med 1990; 323:946-955.

42. Expert Panel on Detection, Evaluation, and Treatment of High Blood Cholesterol in Adults. Summary of the second report of the Nation Cholesterol Education Program (NCEP) Expert Panel on Detection, Evaluation, and Treatment of High Blood Cholesterol in Adults (Adult Treatment Panel II). JAMA 1993;269:3015-3023.

43. Feinlieb M, Gillum RF. Coronary heart disease in the elderly: The magnitude of the problem in the United States. In Wenger NK, Furberg CD, Pitt E (eds): Coronary Heart Disease in the Elderly. New York, Elsevier, 1986, pp. 29-59.

44. Muldoon MF, Manuck SB, Mathews KA. Lowering cholesterol concentrations and mortality: A quantitative review of primary prevention trials. Br Med J 1990; 301:309-314.

45. Canner PL, Berge KG, Wenger NK, et al for the Coronary Drug Project Research Group. Fifteen year mortality in Coronary Drug Project patients: Long term benefit with niacin. J Am Coll Cardiol 1986; 8:1245-1255.

46. Jacobs D, Blackburn H, Higgins M, et al. Report of the conference on low blood cholesterol:mortality associations. Circulation 1992; 86:1046-1060.

47. The Lipid Research Clinics Investigators. The Lipid Research Clinics Coronary Primary Prevention Trial: Results of Six Years of Post-Trial Follow-Up. Arch Int Med 1992; 152:1399-1410.

48. Anderson KM, Castelli WP, Levy D. Cholesterol and mortality: 30 years of follow-up from the Framingham Study. J Am Med Assoc 1987; 257:2176-2180.

49. Stamler J, Wentworth D, Neaton JD, for the MRFIT Research Group. Is relationship between serum cholesterol and risk of premature death from coronary heart disease continuous and graded? Findings in 356,222 primary screenees of the multiple risk factor intervention trial (MRFIT). JAMA 1987; 256:2823-2828.

50. Manolio TA, Pearson TA, Wenger NK, Barrett-Connor E, Payne GH, Harlan, WH. Cholesterol and heart disease in older persons and women: review of an NHLBI workshop. Ann Epidemiol 1992; 2:161-176

51. Gordon DJ, Rifkind BM. Cholesterol-lowering and the older patient. Am J Cardiol 1989; 63:48H-52H.

52. Hoeg JM, Brewer HB. 3-hydroxy-3-methyglutaryl coenzyme A (HMG CoA) reductase inhibitors in the treatment of hypercholesterolemia. J Am Med Assoc 1987; 258:3532-3536.

53. Gordon DJ, Rifkind BM. 3-hydroxy-3-methyglutaryl coenzyme A (HMG CoA) reductase inhibitors: a new class of cholesterol-lowering agents. Ann Intern Med 1987; 107:759-761.

54. Grundy SM. HMG-CoA reductase inhibitors for treatment of hypercholesterolemia. N Engl J Med 1988; 319:24-33.

55. Bradford RH, Shear CL, Chremos AN, Dujovne C, Downton M, Franklin FA, Gould AL, Hesney M, Higgins J, Hurley DP, Langendorfer A, Nash DT, Pool JL, Schnaper H. Expanded Clinical Evaluation of Lovastatin (EXCEL) results. I. Efficacy in modifying plasma lipoproteins and adverse event profile in 8245 patients with moderate hypercholesterolemia. Arch Intern Med 1991; 151:43-49.

56. Brown G, Albers JJ, Fisher LD, Schaefer SM, Lin JT, Kaplan C, Zhao XQ, Bisson BD, Fitzpatrick VF, Dodge HT. Regression of coronary artery disease as a result of intensive lipid lowering therapy in men with high levels of apolipoprotein B. N Engl J Med 1990; 323:1289-1298.

57. Kane JP, Malloy MJ, Ports TA, Phillips NR, Diehl JC, Havel RJ. Regression of coronary artery disease during treatment of familial hypercholesterolemia with combined drug regimens. JAMA 1990; 284:3007-3012.

58. LaRosa J, Applegate W, Crouse J, Hunninghake D, Grimm R, Knopp R, Eckfeldt J, Davis C, Gordon D. Cholesterol lowering in the elderly - Results of the Cholesterol Reduction in Seniors Program (CRISP) Pilot Study. Arch Intern Med 1994;154:529-539.

59. Stamler J, Stamler R, Neaton J. Blood pressure, systolic and diastolic, and cardiovascular risks. U.S. population data. Arch Intern Med 1993;153:598-615.

60. Mantel N. Evaluation of survival data and two new rank order statistics arising in its consideration. Cancer Chemother Rep. 1966;50:163-170.

61. Laird NM, Ware JH. Random effects models for longitudinal data. Biometrics 1982; 38:963-974.

62. Lan KKG and Wittes J. The B-value: A tool for monitoring data. Biometrics 1988; 44:579-585.

63. Davis BR and Hardy RJ. Upper bounds for type I and type II error rates in conditional power calculations. Communications in Statistics 1990; A19(10):3571-3584.

Appendix I: Sample Size Calculations

Power calculations for ALLHAT were done separately for the antihypertensive (AH) and lipid lowering (LL) components. For the AH component power was based on two-tailed comparisons of the diuretic arm to each of the other arms using an overall Type I error rate of .05. This was accomplished using a Dunnett type adjustment for multiple comparisons. For the LL component there was only one comparison, hence no adjustment was needed.

To estimate power for ALLHAT, we had to specify expected event rates, treatment effects, and rates of crossovers and losses to followup from competing risks or other reasons.

Antihypertensive Component

We conservatively estimated the CHD rate in the diuretic arm to be about 1.35% a year (a 6 year rate of approximately 7.8%). This was based on exponential regression models applied to data from Framingham 12, Framingham 16, and HDFP. We included from the Framingham data all hypertensives aged 45-75, excluding those with recent MI (within 2 years). The variables included in the model were age, sex, and whether or not the patient was at high risk (defined as meeting ALLHAT entry criteria). Rates were adjusted to a mean age of *67* and a 55% prevalence of males. A similar analysis was done on the HDFP data, which included those 50 years old and older. The stepped and referred care cohorts of HDFP were analyzed separately. The following reductions were applied for secular trends and the healthy volunteer effect.

Table 1: Adjustment Factors

Trial	Secular Trend Reduction	Volunteer Reduction
Framingham 12	1/2	1/4
Framingham 16	1/3	1/4
HDFP	1/2	1/4

For example, the estimated yearly event rate from the exponential regression of Framingham 12 was multiplied by (1-1/2)(1-1/4)=3/8. The estimated event rates based on the exponential regressions adjusted for secular trends and the healthy volunteer effect were as follows:

Table 2: Estimated Yearly CHD Rates From Framingham and HDFP

Trial	Estimated Event Rates Per Year
Framingham 12	1.32%
Framingham 16	1.42%
HDFP Referred Care	1.76%
HDFP Stepped Care	0.92%

We felt that a 1.35% yearly CHD rate was reasonable. SHEP rates were somewhat higher. SHEP rates automatically incorporate a healthy volunteer effect, and since it was a recent trial, its rates would need less of a secular trend adjustment. We decided to consider a range of event rates from 1.05% a year to 1.65% a year.

Crossover rates were estimated from TOMHS. We fit a time-dependent Markov model to the data. A model assuming a 2.75% chance of crossing over to another medication during each of the first three years, and 6% for each of the last three years appears to fit well (see Figure 1). Under this model, about 24% of all patients will cross over to another medication at least once in 6 years, and about 21% of all patients will be crossed over to another medication at the end of 6 years. A patient in the diuretic arm who crosses over to another active antihypertensive medication is assumed to have a reduced event rate even though some of the other antihypertensives may confer no benefit. A patient in an active antihypertensive arm who crosses over to another antihypertensive medication is assumed to have an increased event rate consistent with the diuretic arm even though that patient may have crossed over to another antihypertensive medication that is as beneficial as that to which he/she was assigned. A patient may cross over and then cross back. We assume that because the physician will have leeway to select among a wide variety of second line antihypertensive medication, there will be a negligible percentage of patients who are taking no medication whatsoever. This assumption is somewhat anti-conservative, but we feel that it is offset by the conservative assumptions alluded to above. We also considered two other rates with 22% and 26% probability of crossing over at least once, respectively. These correspond to approximately 20% and 22.5% of patients on another medication at the end of 6 years, respectively (see Figures 2 and 3). The latter rate appears to be quite conservative. The three rates of 22%, 24%, and 26% for at least one crossover will henceforth be referred to as crossover rates 1, 2, and 3, respectively.

Loss from competing risks was estimated to be approximately 8% over 6 years. This was composed of other cardiovascular mortality (2.6% over 6 years) and non-cardiovascular mortality (5.4% over 6 years). These rates were calculated in a manner similar to the way we computed event rates for the primary endpoint. The same healthy volunteer and secular trend adjustments were applied. We added about 1.5% per year for losses to followup, yielding a total loss rate of approximately 16.8% over 6 years. We considered two other loss rates of

16.8%∀5%. The three loss rates of 11.8%, 16.8% , and 21.8% will henceforth be referred to as loss rates 1, 2, and 3, respectively.

To compute power for the AH component we also had to consider the LL component. It appears from HDFP and Framingham that the patients who qualify for the LL component are at approximately the same risk as those who do not. We therefore made this assumption. Before considering the benefit of antihypertensive medication, we reduced the event rate in the LL active arm by 25% to account for beneficial effects of cholesterol lowering. Note that this is conservative in that it assumes that all of the LL active patients will stay on the drug and receive its full benefit. We assumed that 20,000 of the 40,000 ALLHAT patients would be in the LL component.

We assumed a 20% reduction in event rate in an active antihypertensive arm. We then computed power based on:

1) The optimal allocation of patients to the diuretic and treatment arms, namely the ratio of the number of patients in the diuretic arm to the number in each other AH arm should be (total # arms-1)$^{1/2}$=%3 (see Table 3).

2) An adjustment for comparisons of each treatment to the diuretic (adjusted critical value of approximately c=2.37).

Table 3: Approximate Allocation of Patients

	Antihypertensive Trial			
Cholesterol-lowering trial	Diuretic	Calcium Channel Blocker	ACE Inhibitor	Alpha Blocker*
LL Placebo	3.7k	2.1k	2.1k	2.1k
LL Active	3.7k	2.1k	2.1k	2.1k
Not in LL Component	7.3k	4.2k	4.2k	4.2k

We used a computer program which estimates trial event rates based on yearly rates of events, crossovers, and losses. We ran this program separately for patients in the LL active and LL placebo arms. Based on these values and the allocation of patients specified in Table 3, we obtained trial event rates for the diuretic arm and the other AH arms. For example, using a

* On January 24, 2000, a recommendation to discontinue the doxazosin arm of the antihypertensive trial was accepted by NHLBI. Please refer to JAMA.2000;283:1967-1975.

yearly event rate of approximately .0135, crossover rate 2, and loss 2, we estimate that the trial event rates would be as follows:

Table 4: Estimated 6 Year CHD Rates

LL category	Diuretic Arm	Other AH Arm
LL Placebo	.0710	.0595
LL Active	.0537	.0449
Non LL	.0710	.0595

We estimate the overall event rate in the diuretic arm and another AH arm to be:

$$p_0=(.0710)(.75)+(.0537)(.25)=.0667,$$
$$p_1=(.0595)(.75)+(.0449)(.25)=.0559.$$

An arcsin transformation was used for the test statistic.

$$Z=\frac{\arcsin\left(\sqrt{\hat{p}_0}\right)\arcsin\left(\sqrt{\hat{p}_1}\right)}{\sqrt{(1/4)(1/n_0+1/n_1)}}. \qquad (1)$$

In Formula (1), n_0 and n_1 are the sample sizes in the diuretic and another AH arm, respectively, and p_0 and p_1 are the observed proportion of events in those arms. For the optimal allocation of patients, n_0=40,000[%3/(3+%3)]=14,641 and n_1=40,000[1/(3+%3)]=8,453. We will reject if the Z statistic exceeds the adjusted critical value c=2.37. If we denote the cumulative normal distribution function by Φ(x), power can be shown to be:

$$\Phi\left(\frac{\arcsin\left(\sqrt{p_0}\right)\arcsin\left(\sqrt{p_1}\right)}{\sqrt{(1/4)(1/n_0+1/n_1)}}c\right). \qquad (2)$$

Recall that for our example using a yearly event rate of .0135, crossover rate 2, and loss 2, we found that p_0=.0667 and p_1=.0559. Substituting these values and n_0=14,641 and n_1=8,453 into Formula (2), we get power =.824.

Lipid Lowering Component

The primary endpoint for the lipid lowering component is total mortality. We assume that the vital status of all participants can be ascertained from the National Death Index, hence there will be no loss to followup.

Based on previous experience with HMG CoA reductase inhibitors, we feel that compliance will be quite good, with the bulk of noncompliance occurring early in the trial. We also estimate that given the cost of LL agents and the relatively modest lipid levels of the patients, there will not be many LL placebo patients taking active LL medication. We assume that each year about 2% of all LL placebo patients will take active LL medication with a benefit similar to that of Pravastatin (2% *dropin* per year). This means that about 11.4% of all LL placebo patients will take active medication at least once in 6 years. We further assume that 5% of the LL active patients will stop taking their medication at some point in the first year, and that in each of the remaining years about 2.5% of LL active patients will stop taking their medication (*dropout* rate of 5% in the first year and 2.5% each year thereafter). This corresponds to approximately 16.3% of LL active patients stopping their medication at least once in 6 years. Under the above assumptions, approximately 10.6% of all LL placebo patients will be taking active medication at the end of 6 years, and about 15.3% of the LL active patients will be off their medication at the end of 6 years. We also considered a more pessimistic set of assumptions, namely a yearly dropin rate of 2.5% and a dropout rate of 6% during the first year and 3% a year thereafter. The first set of assumptions will be referred to as dropin/dropout 1, and the more pessimistic set of assumptions will be referred to as dropin/dropout 2.

We estimated the mortality rate to be approximately 2.35% per year. This estimate was based on separate incidence rates for CHD mortality (between 4.5% and 5% over 6 years), other cardiovascular mortality (about 2.6% over 6 years), and non-cardiovascular mortality (about 5.4% over 6 years). These separate rates were estimated using the parametric regression methods we used for the primary endpoint.. The same secular trend and healthy volunteer adjustments were made as were made for the AH component. This gave an estimated mortality rate between 2.25% and 2.30% per year. We increased this to 2.35%/year based on a somewhat higher mortality rate observed in SHEP. We considered a range of yearly mortality rates between 2.20% and 2.50%.

It is difficult to estimate a reasonable percent reduction in mortality from LL medication. A 14% reduction would occur if there were a 30% reduction in CHD death, a 15% reduction in other cardiovascular mortality, and no reduction in non-cardiovascular mortality. This seems a little optimistic. We felt that a 12.5% reduction was reasonable, so we considered three different percent reductions, 11%, 12.5%, and 14%.

We reduced the mortality rate in the three AH arms other than the diuretic by 10%.

Six year mortality rates were computed for the LL placebo and LL active arms in a manner similar to the calculations for the AH component. Separate rates were computed for the diuretic arm and other AH arms, and a combined rate was obtained using the allocations shown in Table 3. The 6 year rates in the LL component of ALLHAT are shown in Table 5 below.

Table 5: Estimated 6 Year Mortality Rates

LL category	Diuretic Arm	Each Other AH Arm
LL Placebo	.13253	.11998
LL Active	.11903	.10769

The 6 year rates in the LL placebo and active arms are therefore:

p_0=[%3/(3+%3)](.13253)+[3/(3+%3)](.11998)=.12457
p_1=[%3/(3+%3)](.11903)+[3/(3+%3)](.10769)=.11184

Power was again computed using the arcsin transformation. The only differences between this calculation and the AH calculation are that the sample sizes n_0 and n_1 in the LL placebo and active arms are equal, and there is no adjustment for multiple comparisons. Thus we can use Formula 2 with $n_0=n_1=10,000$ and c=1.96.

Results

Power for the AH component under different assumptions is depicted in Figures 4-6. There is not a great difference in power under the different assumptions. Looking at crossover rate 2 and loss 2, we see that the power under the anticipated diuretic rate of 1.35% per year is 82.4%, as we calculated above.

Power for the LL total mortality component is depicted in Figure 7. We see that the power is almost exactly 80% under dropin/dropout rate 1 and a 12.5% reduction in mortality from LL treatment. It drops to 68.6% if there is only an 11% reduction in mortality (other assumptions as before). On the other hand, the power will be 88.1% if there is a 14% reduction in mortality from LL treatment (other assumptions as before). Under the more pessimistic dropin/dropout rate 2, the power is 76.9% under a 2.35%/yr mortality rate and a 12.5% reduction in mortality from LL treatment (not shown in the graph).

Figure 1
Crossover Rates From TOMHS

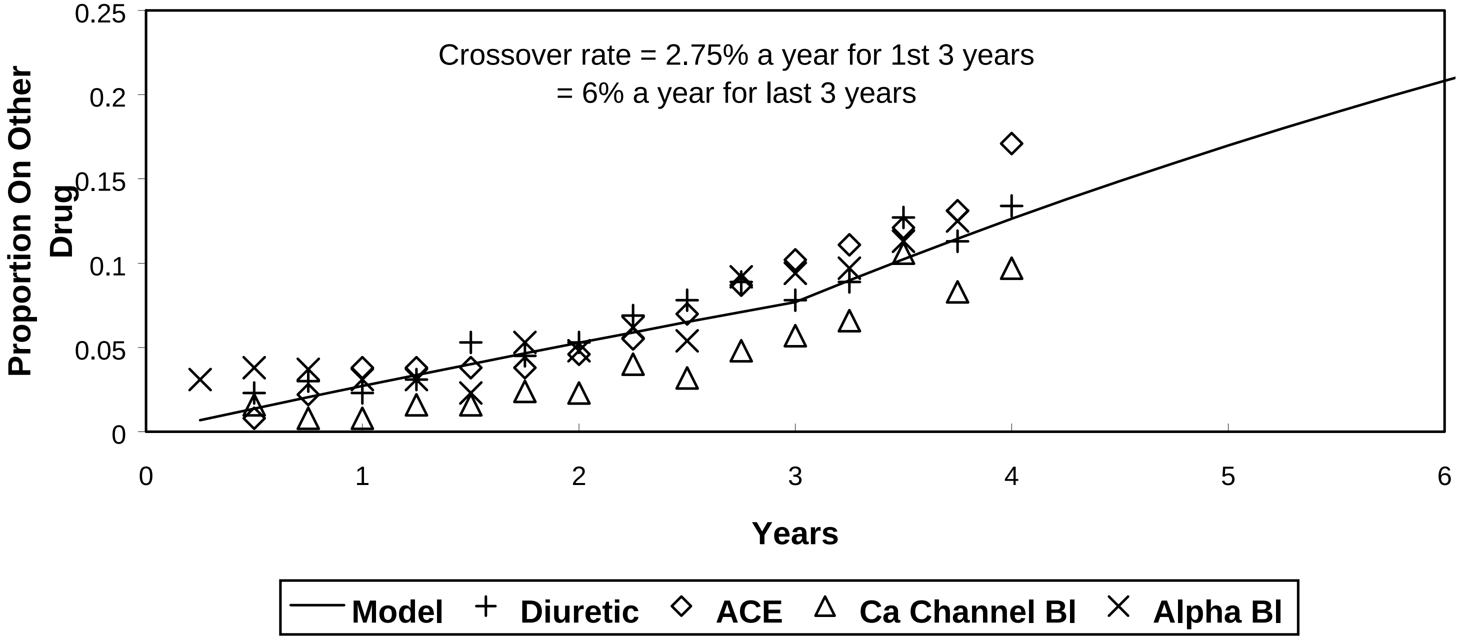

Figure 2
Crossover Rates From TOMHS

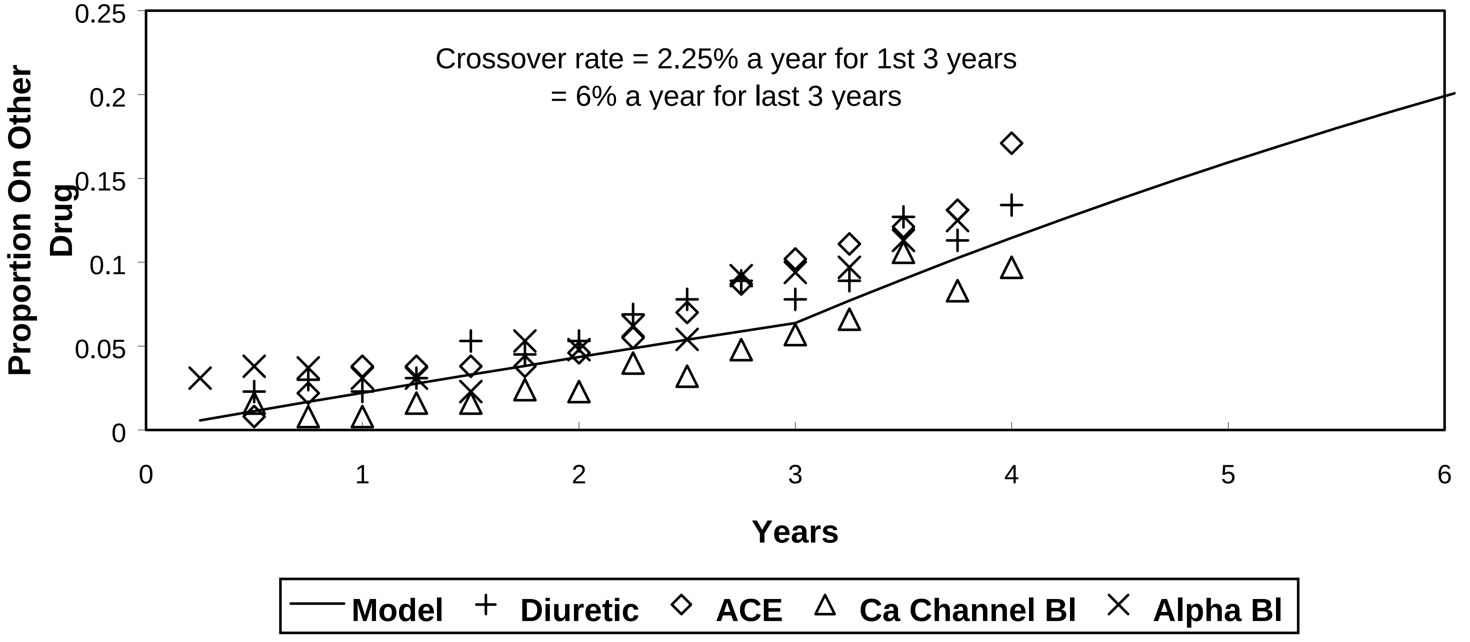

Figure 3
Crossover Rates From TOMHS

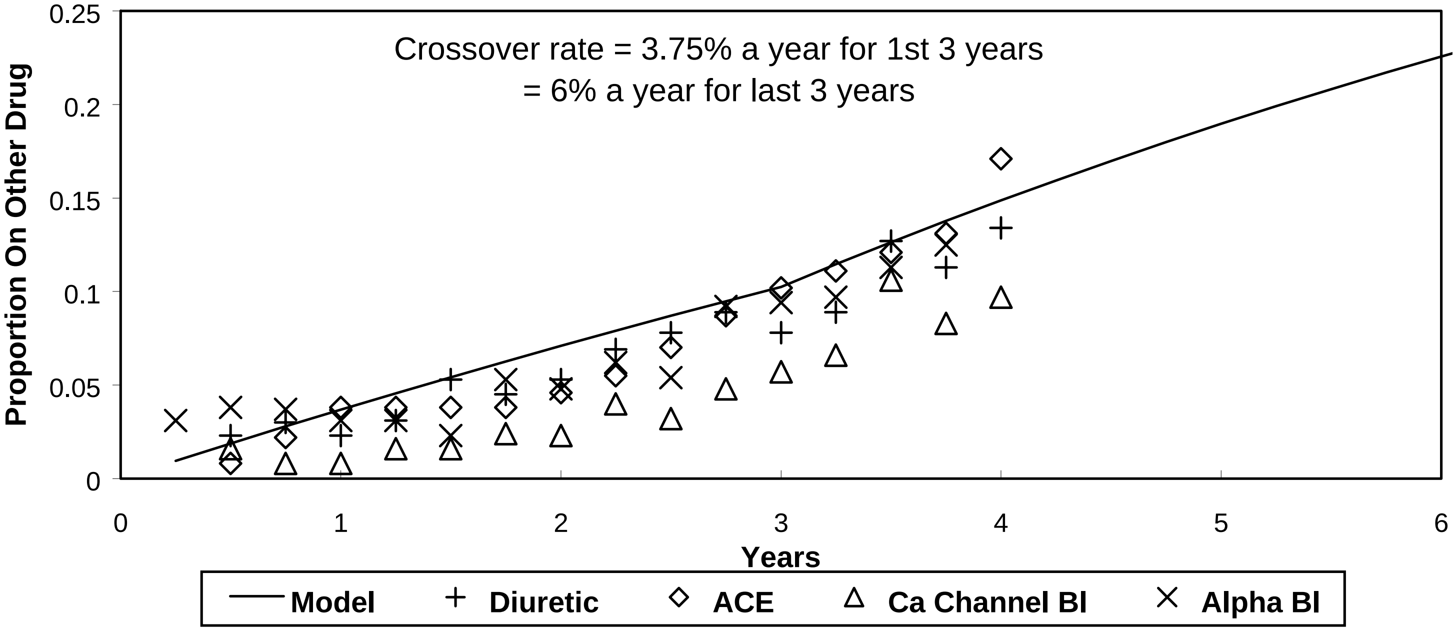

Figure 4 - Power for Hypertension Component - Crossover Rate 1

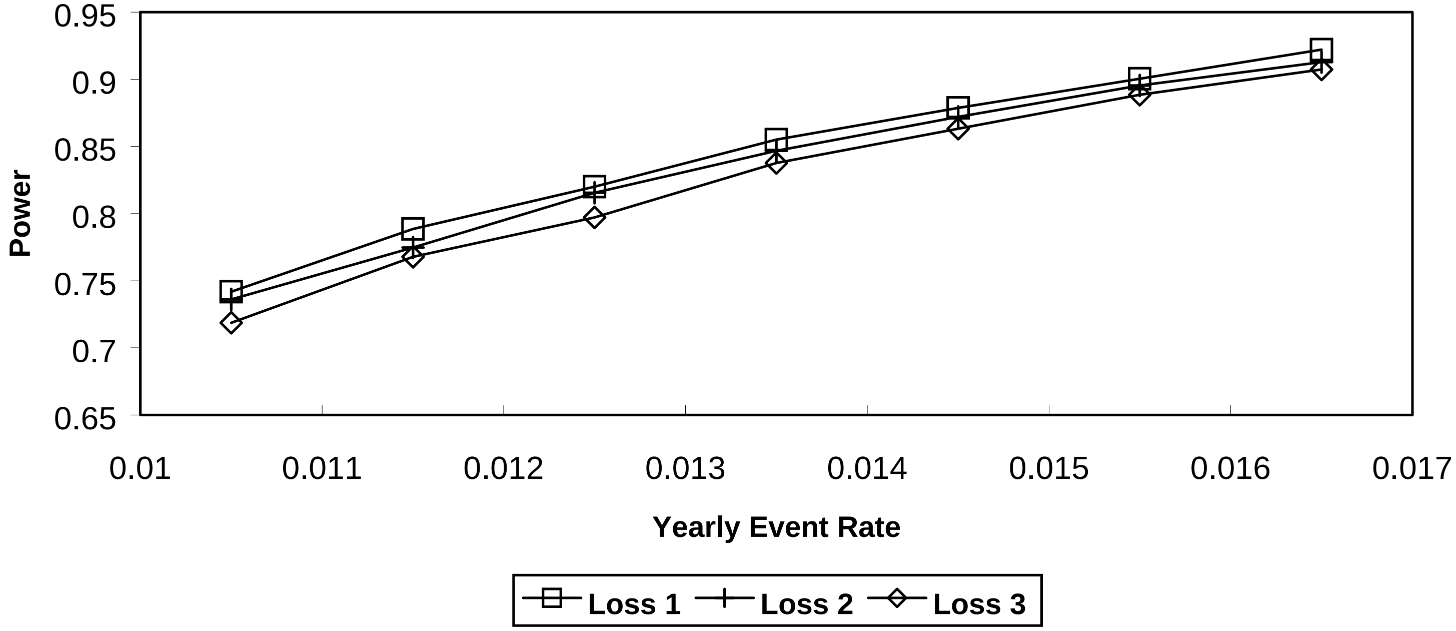

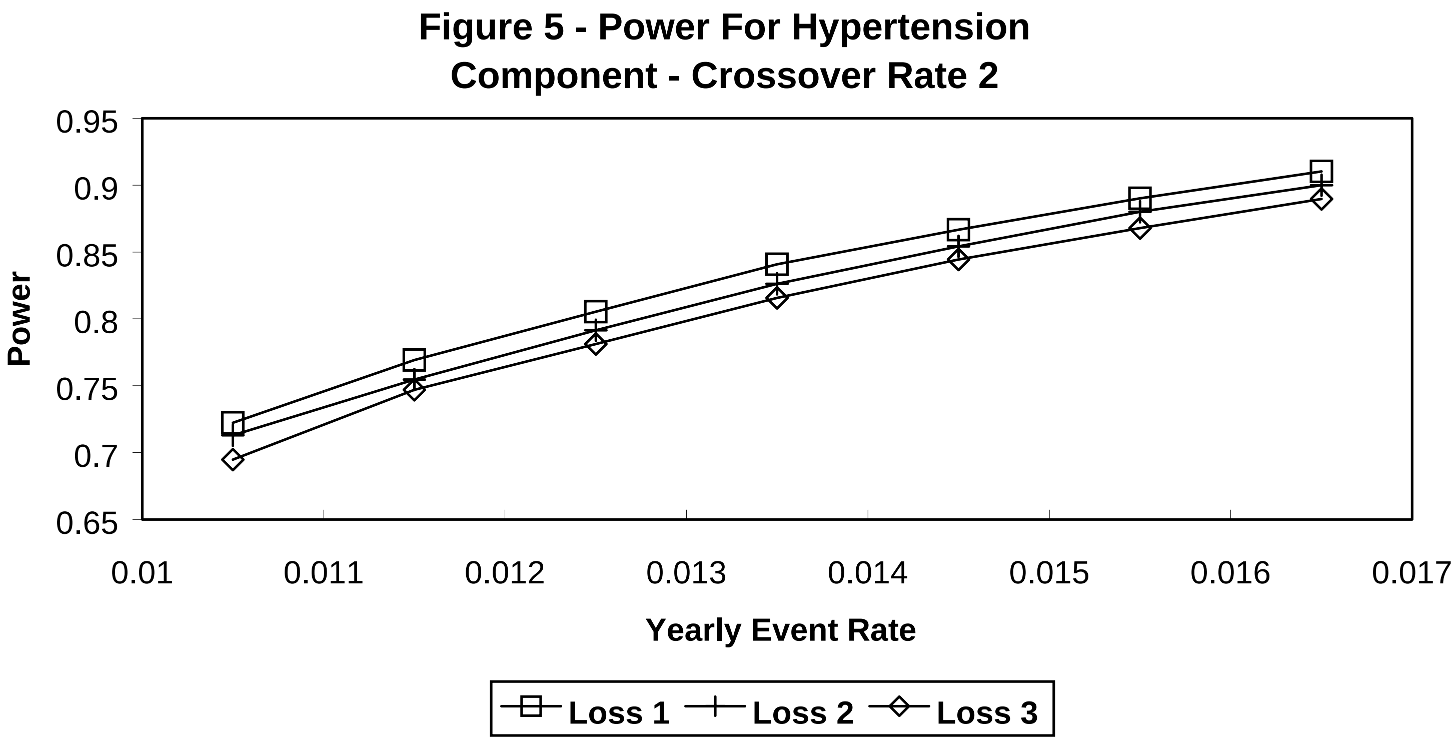
Figure 5 - Power For Hypertension
Component - Crossover Rate 2
0.95
0.9
0.85
0.8
0.75
0.7
0.65
Power
0.01
0.011
0.012
0.013
0.014
0.015
0.016
0.017
Yearly Event Rate
Loss 1
Loss 2
Loss 3

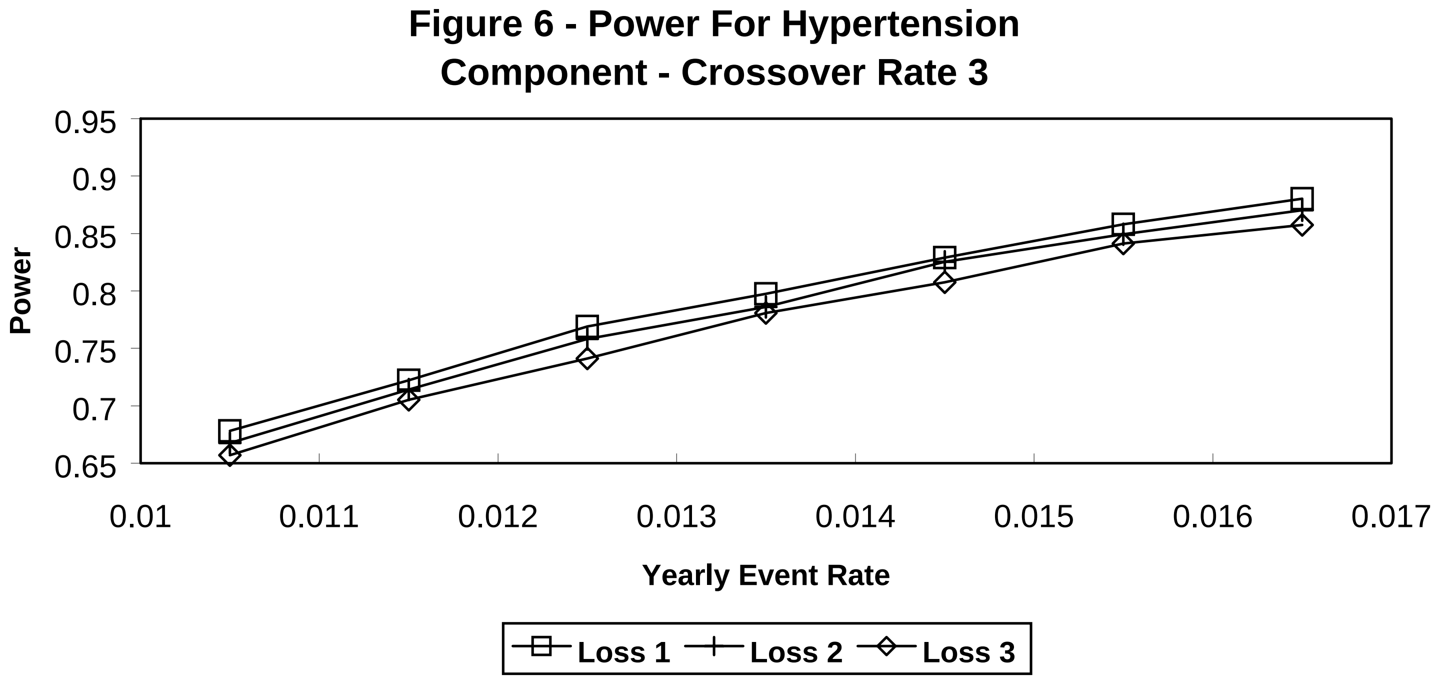
Figure 6 - Power For Hypertension
Component - Crossover Rate 3
0.95
0.9
0.85
0.8
0.75
0.7
0.65
Power
0.01
0.011
0.012
0.013
0.014
0.015
0.016
0.017
Yearly Event Rate
Loss 1
Loss 2
Loss 3

Figure 7 - Power For Lipid-Lowering Component - Total Mortality

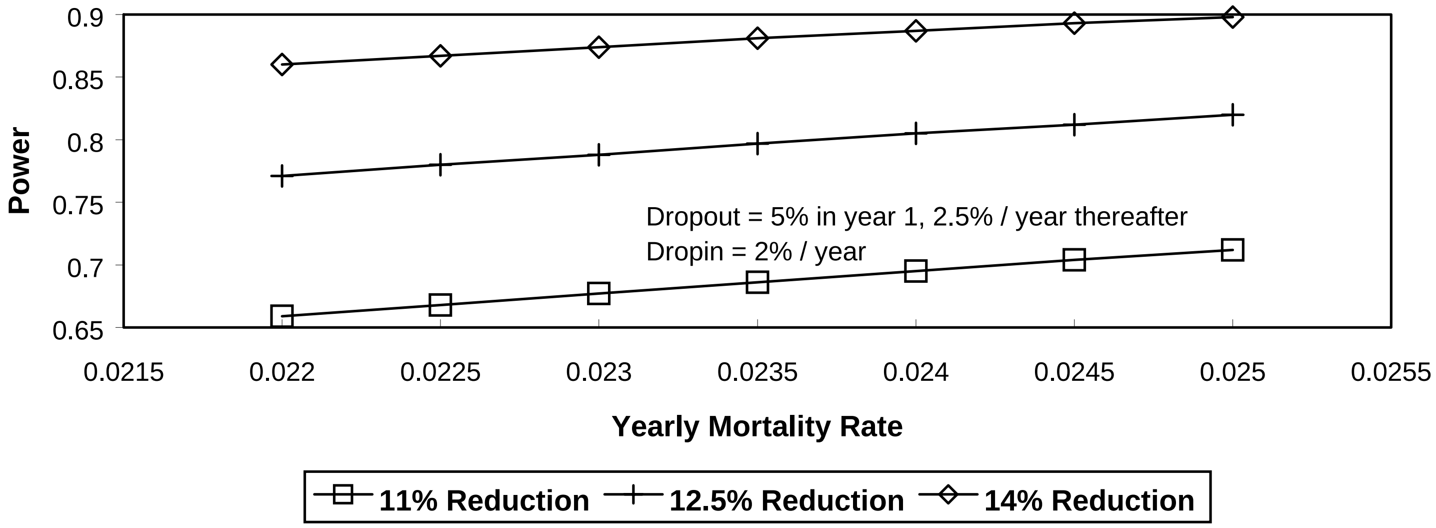

Appendix II: Detailed Definitions of Coronary Death, Nonfatal Acute Myocardial Infarction and Stroke

Coronary Death:

Death consistent with coronary heart disease (CHD) as underlying cause on death certificate, plus any one of the following:

1) Pre-terminal hospitalization with myocardial infarction,
2) Previous angina or myocardial infarction and no known potentially lethal non-coronary disease process
3) Death within 24 hours of symptoms of CHD or death within 24 hours without symptoms but with no known potentially lethal non-coronary disease process (includes instantaneous death and unwitnessed death)
4) Death resulting from a procedure related to coronary artery disease such as coronary bypass grafting (CABG) or percutaneous transluminal coronary angioplasty (PTCA).

Note: Deaths due to a non-coronary underlying cause in which the terminal event was an MI shall be ascribed to the underlying cause -- not to CHD.

Coronary death will be subclassified as:

1) Definite fatal MI: no known non-atherosclerotic cause and definite MI within 4 weeks of death,
2) Definite fatal CHD: no known non-atherosclerotic cause and one or both of the following: chest pain within 72 hours of death or a history of chronic ischemic heart disease in the absence of valvular heart disease or non-ischemic cardiomyopathy, or
3) Possible fatal CHD: no known non-atherosclerotic cause, and death certificate consistent with underlying cause.

   In patients without a known potentially lethal non-coronary disease process, coronary death will also be classified as rapid or non-rapid, based on whether death did or did not occur within 24 hours after the onset of symptoms (or the time at which the patient was last seen without symptoms).

→ **Nonfatal Acute Myocardial Infarction**

The identification and coding of definite clinical acute myocardial infarction (MI) should be based on meeting at least two of the following three generally accepted criteria, consistent with the World Health Organization criteria:

1. Symptoms (such as chest pain) compatible with an acute MI of at least 20 minutes duration.

2. ECG changes compatible with an acute MI, such as new persistent ST segment elevation of

∃0.1 mV or new pathologic Q waves (QRS>0.04 sec), each in two contiguous leads.

→ 3. A serum biochemical marker compatible with an acute MI, such as:

- ✓ Total CK at least twice the upper limits of normal with an MB fraction of >5% of total CK, or
- ✓ Troponin >2 times the upper limit of normal, or
- ✓ LDH1/LDH2 ratio ∃1.

<u>Stroke</u>

Rapid onset of persistent neurologic deficit attributable to an obstruction or rupture of the arterial system, including stroke occurring during surgery, that is not known to be secondary to brain trauma, tumor, infection, or other non-ischemic cause. The deficit must last more than 24 hours unless death supervenes or there is a demonstrable lesion compatible with acute stroke on CT or MRI scan.

A. Non-fatal stroke (either of the following):

1) Unequivocal objective findings of a localizing neurologic deficit (with or without recent onset of severe headache or loss of consciousness), AND duration longer than 24 hours AND absence of other disease process causing neurologic deficit such as neoplasm, subdural hematoma, cerebral angiography, or metabolic disorder, and/or

2) Diagnosis of stroke based on abnormality demonstrated by CT or MRI consistent with current neurologic symptoms or signs, or positive lumbar puncture (for subarachnoid hemorrhage).

B. Fatal stroke: Death certificate listing stroke as consistent with, underlying, or immediate cause of death, plus any one or more of the following:

1) Preterminal hospitalization with stroke as defined above,

2) Previous stroke and no known potentially lethal non-cerebrovascular disease process, and/or

3) Stroke diagnosed as cause of death at post-mortem examination.

Appendix III: Electrocardiographic Criteria for Silent Myocardial Infarction, Ischemia, Left Ventricular Hypertrophy, and Bundle Branch Block

The ALLHAT study records electrocardiographic information from baseline and biennial clinic visits and from a sample of acute hospitalizations that are designated as quality control events. Clinic ECGs are evaluated for prevalent and interim events including "silent" myocardial infarction.

Prevalent and Interim ECG Findings at Clinic Visits

A determination that an ALLHAT participant has prevalent MI, ischemia, LVH or bundle branch block can be made using Minnesota Code criteria. Interim MI, ischemia, LVH or bundle branch block can be made using the criteria shown for simultaneous comparison of ECGs.

Prevalent Baseline ECG Findings

1. Prevalent MI
   Baseline ECG coded:
   a) any 1 -1 -x code
   OR
   b) any 1-2-x PLUS 4-1-x, or 4-2, or 5-1, or 5-2

2. Prevalent Ischemia
   Baseline ECG coded:
   a) any 4-2 through 4-1-x
   b) any 5-2 through 5-1

3 Prevalent LVH
   Baseline ECG coded:
   a) 3-1 or 3-3 (soft LVH)
   b) 3-1 or 3-3 PLUS any 4-3 through 4-1-x, or 5-3 through 5-1 (hard LVH)

4. Prevalent bundle branch block
   Baseline ECG coded:
   a) 7-l-l
   b) 7-2-1
   c) 7-4

Interim ECG Events

An evolving ECG pattern between the baseline visit and an ECG from a later visit confirmed by simultaneous ECG comparison documents the interim event.

a. Interim Ml

Any ED1 through ED7

b. Interim ischemic event
Any EV1 -EV9 pattern

c. Interim LVH or progression / regression of LVH
E-LVH 1 through E-LVH 4

d. Interim bundle branch block
EBBB 1 through EBBB 3

Attachment 1
Simultaneous Comparison of ECGs

Simultaneous ECG Comparison Explanations:

- A code 1-2-6 is considered no Q-code for the purposes of serial comparison.
- An Equivocal Q-code is a 1-2-8 or any 1-3-x code.
- A Diagnostic Q-code is any 1-1-x or any 1-2-x except 1-2-6 or 1-2-8.
- The designation of "ED" means evolving diagnostic Q-code pattern.
- All ED patterns are confirmed as significant increase by serial comparison.
- An EDl through ED7 cannot be assigned if a 7-1-1 code is present.
- An ED2 through ED7 cannot be assigned if a 7-2-1 or 7-4 code is present.
- The designation "EV" means evolving ST-T wave pattern.
- All EV patterns are confirmed as significant increases or decreases by serial comparison.
- An EVl through EV9 cannot be assigned if a 7-1-1, 7-2-1, or 7-4 code is present.

Significant Serial Change Patterns

Definite Q-wave MI (evolving diagnostic pattern)

EDl. No Q-code (or a 1-2-6) in baseline ECG followed by a record with a Diagnostic Q-code (Minnesota Code 1-1-1 through 1-2-5 or 1-2-7), confirmed as a significant increase.

OR

A 1-2-8 or any 1-3-X code in baseline ECG followed by a record with any l-l-X code, confirmed as a significant increase.

ED2a. An Equivocal Q-code (l-2-8 or any 1-3-x code) and no major ST depression in baseline ECG followed by a record with a Diagnostic Q-code (l-1 -1 to 1 -2-5 or 1-2-7) PLUS a major ST depression (4-1-X or 4-2), confirmed as a significant increase.

ED2b. An Equivocal Q-code (l-2-8 or any 1-3-x code) with pre-existing major ST depression (4-1-X or 4-2) in baseline ECG followed by a record with a Diagnostic Q-code (l-1 -1 to 1-2-5 or 1-2-7) PLUS more severe ST depression (4-1-X), confirmed by a significant increase.

ED3a. An Equivocal Q-code (l-2-8 or any 1-3-x code) and no major T-wave inversion in baseline ECG followed by a record with a Diagnostic Q-code (l-1-1 to 1-2-5 or 1-2-7) PLUS a major T-wave inversion (5-1 or 5-2), confirmed as a significant increase.

ED3b. An Equivocal Q-code (l-2-8 or any 1-3-x code) with pre-existing major T-wave inversion (5-1 or 5-2) in baseline ECG followed by a record with a Diagnostic Q-code (l-1 -1 to 1 -2-5 or 1 -2-7) PLUS more severe T-wave inversion (5-1 or 5-2), confirmed as a significant increase.

ED4a. An Equivocal Q-code (1-2-8 or any 1-3-x code) and no ST elevation in baseline ECG followed by a record with a Diagnostic Q-code (l-1 -1 to 1 -2-5 or 1 -2-7) PLUS an ST segment elevation (9-2), confirmed as a significant increase.

ED4b. An Equivocal Q-code (l-2-8 or any 1-3-x code) with pre-existing ST elevation (9-2) in the baseline ECG followed by a record with a Diagnostic Q-code (l-1 -1 to 1 -2-5 or 1 -2-7) PLUS more severe ST elevation (9-2), confirmed as a significant increase.

ED5. No Q-code (or a 1-2-6) and neither 4-1-X nor 4-2 in baseline ECG followed by a record with an Equivocal Q-code (l-2-8 or any 1-3-x code) PLUS 4-1-X or 4-2, confirmed as a significant increase.

ED5b. No Q-code (or a 1 -2-6) with pre-existing major ST depression (4-1 -x to 4-2) in baseline ECG followed by a record with an Equivocal Q-code (l-2-8 or any 1-3-x code) PLUS more severe ST depression (4- 1-X), confirmed as a significant increase.

ED6a. No Q-code (or a 1 -2-6) and neither 5-1 nor 5-2 in baseline ECG followed by a record with an Equivocal Q-code (l-2-8 or any 1 -3-x code) PLUS a 5-1 or 5-2, confirmed as a significant increase.

ED6b. No Q-code (or a 1 -2-6) with pre-existing major T-wave inversion (5-1 or 5-2) in baseline ECG followed by a record with an Equivocal Q-code (1-2-8 or any 1-3-x code) PLUS more severe T-wave inversion (5-1 or 5-2), confirmed as a significant increase.

ED7a. No Q-code (or a 1 -2-6) and no 9-2 in baseline ECG followed by a record with an Equivocal Q-code (l-2-8 or any 1-3-x code) PLUS a 9-2, confirmed as a significant increase.

ED7b. No Q-code (or a 1 -2-6) with pre-existing ST elevation (9-2) in the baseline ECG followed by a record with an Equivocal Q-code (1-2-8 or any 1-3-x code) PLUS more severe ST elevation (9-2), confirmed as a significant increase.

Diagnostic ECG:

D1. An ECG record with a Diagnostic Q-code (l-1-1 to 1-2-5 or 1-2-7).

D2. An ECG record with ST segment elevation (9-2) PLUS T wave inversion (5-1 or 5-2).

Evolving ST-T Pattern:

This diagnosis cannot be assigned if a 7-1-1, 7-2-1, or 7-4 is present. For hospitalized participants, the EV patterns can occur from either increases or decreases in the severity of the code.

EV1. Either 4-0 (no 4-code), 4-4 or 4-3 in baseline ECG followed by a record with 4-1-1, 4-1-2, or 4-2, confirmed as a significant increase; OR for hospital ECGs only, 4-1-1, 4-1-2, or 4-2 in the earliest hospital ECG followed by an event record with a 4-0, 4-4, or 4-3, confirmed as a significant decrease.

Plus

Either no Q-code in both the baseline ECG and the follow-up ECG OR Q-code(s) present in baseline ECG or follow-up ECG but no significant increase in Q codes found.

EV2. Either 4-2 or 4-1-2 in baseline ECG followed by a record with 4-1 -1, confirmed as a significant increase, or a 4-2 in baseline followed by a record with 4-1-2, confirmed as a significant increase; OR for hospital ECGs only, 4-1-1 in the earliest hospital ECG followed by an event record with a 4-1-2 or 4-2, confirmed as a significant decrease, OR 4-1-2 in the baseline ECG followed by a 4-2 in the follow-up ECG, confirmed as a significant decrease.

Plus

Either no Q-code in both the baseline ECG and the follow-up ECG OR Q-code(s) present in baseline ECG or follow-up ECG but no ignificant increase in Q codes found.

EV3. Either 5-0, 5-4 or 5-3 in baseline ECG followed by a record with 5-2 or 5-1, confirmed as a significant increase; OR for hospital ECGs only, 5-1 or 5-2 in the earliest hospital ECG followed by an event record with a 5-0, 5-4 or 5-3, confirmed as a significant decrease.

Plus

Either no Q-code in both the baseline ECG and the follow-up ECG OR Q-code(s) present in baseline ECG or follow-up ECG but no significant increase in Q codes found.

EV4. Code 5-2 in baseline ECG followed by a record with 5-1, confirmed as a significant increase; OR for hospital ECGs only, 5-1 in the earliest hospital ECG followed by an event record with a 5-2, confirmed as a significant decrease.

Plus

Either no Q-code in both the baseline ECG and the follow-up ECG OR Q-code(s) present in baseline ECG or follow-up ECG but no significant increase in Q codes found.

EV5. Code 9-0 in baseline ECG followed by a record with 9-2, confirmed as a significant increase; OR for hospital ECGs only, 9-2 in the earliest hospital ECG followed by an event record with a 9-0, confirmed as a significant decrease.

Plus

Either no Q-code in both the baseline ECG and the follow-up ECG OR Q-code(s) present in baseline ECG or follow-up ECG but no significant increase in Q codes found.

EV6. Code 4-1-1 in baseline ECG followed by a record with 4-1-1, confirmed as a significant increase; OR for hospital ECGs only, 4-1-1 in the earliest hospital ECG followed by an event record with a 4-1-1, confirmed as a significant decrease.

Plus

Either no Q-code in both the baseline ECG and the follow-up ECG OR Q-code(s) present in baseline ECG or follow-up ECG but no significant increase in Q codes found.

EV7. Code 5-1 in baseline ECG followed by a record with 5-1, confirmed as a significant increase; OR for hospital ECGs only, 5-1 in the earliest hospital ECG followed by an event record with a 5-1, confirmed as a significant decrease.

Plus

Either no Q-code in both the baseline ECG and the follow-up ECG OR Q-code(s) present in baseline ECG or follow-up ECG but no significant increase in Q codes found.

EV8. Code 5-2 in baseline ECG followed by a record with 5-2, confirmed as a significant increase; OR for hospital ECGs only, 5-2 in the earliest hospital ECG followed by an event record with a 5-2, confirmed as a significant decrease.

Plus

Either no Q-code in both the baseline ECG and the follow-up ECG OR Q-code(s) present in baseline ECG or follow-up ECG but no significant increase in Q codes found.

EV9a. No Q-code (or a 1-2-6) and pre-existing major ST depression, T wave inversion, or ST elevation (4-2 / 4-1-x, 5-2 / 5-1, or 9-2) in baseline ECG followed by an Equivocal Q-code (1-2-8 or any 1-3-x code) and less severe or absent ST depression, T wave inversion, or ST elevation (4-0 /4-4 / 4-3 / 4-2 / 4-1-x, 5-0 / 5-3 / 5-2 / 5-1, or 9-0 / 9-2), with Q-code confirmed as a significant increase, and ST or T wave changes confirmed as a significant decrease.

EV9b. An Equivocal Q-code (1-2-8 or any 1-3-x code) and pre-existing major ST depression, T wave inversion, or ST elevation (4-2 / 4-1-x, 5-2 / 5-1, or 9-2) in baseline ECG followed by a Diagnostic Q-code (1-1-1 to 1-2-5 or 1-2-7) and less severe or absent ST depression, T wave inversion, or ST elevation (4-0 / 4-4 /4-3 / 4-2 / 4-1-x, 5-0 / 5-3 / 5-2 / 5-1, or 9-0 / 9-2), with Q-code confirmed as a significant increase, and ST or T wave changes confirmed as a significant decrease.

Evolving Bundle Branch Block

E-BBB l. No 7-1-1 in reference followed by an ECG with 7-1-1 with the QRS duration increased by ∃ 0.02 sec., confirmed as a significant increase.

E-BBB 2. No 7-2-1 in reference followed by an ECG with 7-2-1 with the QRS duration increased by ∃ 0.02 sec., confirmed as significant increase.

E-BBB 3. No 7-4 in reference followed by an ECG with 7-4 with the QRS duration increased by ∃ 0.02 sec., confirmed as a significant increase.

Evolving LVH

E-LVH 1. No 3-1 in the reference ECG followed by an ECG with a 3-1, confirmed as a sigificant increase.

E-LVH 2. No 3-3 in the reference ECG followed by an ECG with a 3-3, confirmed as a significant increase.

E-LVH 3. 3-1 in the reference ECG followed by an ECG with 3-1 or no 3 code confirmed as a significant decrease.

E-LVH 4. 3-3 in the reference ECG followed by an ECG with 3-3 or no 3 code confirmed as a significant decrease.

Equivocal ECG Pattern:

EI. An ECG record with a 1-2-8; or an ECG record with a 1-3-x in the absence of 7-2-1 or 7-4.

E2. An ECG record with ST-segment depression (4-1-x, 4-2, or 4-3).

E3. An ECG record with T-wave inversion (5-1, 5-2, or 5-3).

E4. An ECG record with ST-segment elevation code 9-2.

Other ECG Pattern:

All other ECG findings, including normal.

Uncodable ECG Pattern:

UI. Technical errors coded 9-8-1 by Minnesota Code.

a. Three or more missing leads.

b. Muscle tremor artifact that produces possible false initial R's.

c. Other technical errors making Q-wave measurement impossible, such as extreme lack of centering, or marked clipping.

d. Other conditions defined as "uncodable" by the Minnesota Code.

Absent ECG:

A. No ECG available for coding.

→ **Appendix IV: Quality Control Evaluation of Hospitalized Myocardial Infarctions (MI)**

The following definitions for acute hospitalized MI are not intended for the classification on the AL04 by site investigators, but rather for quality control assessment review by the Endpoints Subcommittee.

The criteria presented are based on the CCSP Pilot Study, the Minnesota Heart Survey, and other surveillance studies as incorporated into the ARIC study. The combinations of pain, ECG and enzyme categories required for each diagnosis ~~below~~ are approximately the same as those contained in the above-mentioned documents.

Definite Hospitalized MI

Must meet one or more of the following criteria:

1. Evolving diagnostic ECG pattern (ED1 - ED7) (Appendix III, Attachment 1)

OR

2. Diagnostic ECG pattern (D1 or D2) and abnormal enzymes (Appendix III, Attachment 1)

OR

3. Cardiac pain (defined below) and abnormal enzymes

AND

a. Evolving ST-T pattern EV1-EV9 (Appendix III, Attachment 1)

OR

b. Equivocal ECG pattern E1 through E4 (Appendix III, Attachment 1)

Probable in-hospital MI

Must meet one or more of the following criteria in the absence of sufficient evidence for Definite Hospitalized MI:

1. Cardiac pain and abnormal enzymes

or

2. Cardiac pain and equivocal enzymes and

a. Evolving ST-T pattern

or

b. Diagnostic ECG pattern

or

3. a. Abnormal enzymes and

b. Evolving ST-T pattern

Suspect in-hospital MI

Must meet one or more of the following criteria in the absence of sufficient evidence for Definite or Probable in-Hospital MI.

1. Abnormal enzymes

or

2. Cardiac pain and incomplete enzymes and

a. Diagnostic ECG pattern

or

b. Evolving ST-T pattern

or

3. Cardiac pain and equivocal enzymes

or

4. Equivocal enzymes and

a. Diagnostic ECG pattern

or

b. Evolving ST-T pattern

or

c. Equivocal ST-T pattern

The definitions of specific elements of chest pain, enzymes and ECGs which contribute to the final diagnosis of definite, probable, suspect, or no MI are provided below.

Definition of Cardiac Pain

Pain having both of the following characteristics:

1. It occurs anywhere in the anterior chest, left arm, or jaw.

    and

2. Absence of a definite non-cardiac cause of pain.

Abnormal Cardiac Enzymes

Enzymes are classed as abnormal if any enzyme values recorded meet any of the following criteria:

1.a. CK-MB is "present": (if laboratory uses the criterion of "present" or "absent" without reporting a more specific value) or the CK-MB is greater than or equal to 10% of the total CK value,

    and

b. There is no known non-ischemic cause (cardiac surgery, severe muscle trauma, rhabdomyolysis) for the elevated enzyme value.

    or

2.a. The ratio $LDH_1 : LDH_2 \exists 1$.

    and

b. There is no evidence of hemolytic disease.

    or

3.a. Total CK and LDH are both at least twice the upper limits of normal. (These increases do not have to occur on the same day).

    and

b. There is no known non-ischemic cause (cardiac surgery, severe muscle trauma, rhabdomyolysis) for the elevated enzyme value and no evidence of hemolytic disease.

Equivocal Cardiac Enzymes

Enzymes are classed as "equivocal" if the criteria for abnormal enzymes are not met <u>and</u> if:

1. Either total CK <u>or</u> total LDH are at least twice the upper limits of normal.

or

2. Both total CK <u>and</u> total LDH are between the upper limits of normal and twice the upper limits of normal. (These increases do not have to occur on the same day.)

or

3. CK-MB - 5-9% of total CK or is "weakly present."

A summary of the enzyme diagnostic criteria, as related to total CK and LDH is given in the following algorithm, Table 1.

Table 1. Algorithm for Total CK and LDH Enzyme Diagnostic Criteria

	Total CK		
	Upper Limit of Normal	Twice Upper Limit of Normal	
TOTAL LDH	Equivocal	Equivocal	Abnormal
Twice Upper Limit of Normal	Normal	Equivocal	Equivocal
Upper Limit of Normal	Normal	Normal	Equivocal

Table 2
Proposed ALLHAT Diagnostic Criteria for In-Hospital MI

Cardiac Pain	ECG Findings	Enzymes	Diagnosis
**Present**	Evolving Diagnostic Pattern (EDl-ED7)	Abnormal	Definite MI
		Equivocal	Definite MI
		Incomplete	Definite Ml
		Normal	Definite MI
	Diag. ECG Pattern	Abnormal	Definite MI
		Equivocal	Probable MI
		Incomplete	Suspect MI
		Normal	No MI
	Evolving ST-T (EVl - EV9)	Abnormal	Definite MI
		Equivocal	Probable MI
		Incomplete	Suspect MI
		Normal	No MI
	Equivocal ECG	Abnormal	Definite MI
	Pattern Equivocal	Probable MI	
		Incomplete	No MI
		Normal	No MI
	Absent, Uncodable, or other	Abnormal	Probable MI
		Equivocal	Suspect MI
		Incomplete	No MI
		Normal	No MI
**Not Present, Unknown or Missing**	Evolving Diagnostic Pattern (EDl-ED7)	Abnormal	Definite MI
		Equivocal	Definite MI
		Incomplete	Definite Ml
		Normal	Definite MI
	Diag. ECG Pattern	Abnormal	Definite MI
		Equivocal	Suspect MI
		Incomplete	No MI
		Normal	No MI

Evolving		Abnormal	Probable MI
ST-T Pattern	Equivocal	Suspect MI	
(EV1-EV9)		Incomplete	No MI
		Normal	No MI
Equivocal ECG		Abnormal	Suspect MI
Pattern	Equivocal	Suspect MI	
		Incomplete	No MI
		Normal	No MI
Absent, Uncodable		Abnormal	Suspect MI
or other		Equivocal	No MI
		Incomplete	No MI
		Normal	No MI

PROTOCOL ADDENDUM 1
Revised Power for Lipid-Lowering Portion of ALLHAT

Power for the lipid-lowering portion of ALLHAT was revised based on a total sample size of 10,000 and subgroups of size 4,000. The dropout rate was taken as the more pessimistic of the two scenarios in the protocol-6% in the first year and 3% per year thereafter. The dropin was also taken as the more pessimistic of the two protocol scenarios, namely 2.5% per year. The loss for total mortality was taken to be 0, while for the CHD it was as specified in the protocol for the antihypertensive component (slightly over 3% per year). The event rates shown are after accounting for benefits of antihypertensive therapy. The power results are shown in Tables 1 and 2. A more pessimistic loss of 5% per year was also considered. This reduced power by about 2 percentage points.

Table 1: Power for total mortality
Loss assumed to be 0, dropout=6% in first year, 3% per year thereafter, dropin=2.5% per year.

Treatment Reduction	Control Rate/year	N = 4,000	N = 10,000
	.01		
	.015		
10% reduction	.020		
	.025		
	.030	19%	41%
	.01	13%	25%
	.015	17%	35%
12.5% reduction	.02	21%	44%
	.025	24%	52%
	.030	28%	59%
	.01	16%	34%
	.015	22%	47%
15% reduction	.02	28%	58%
	.025	33%	68%
	.030	39%	75%
	.01	26%	55%
	.015	37%	72%
20% reduction	.02	46%	84%
	.025	54%	91%
	.030	62%	95%
	.01	39%	76%
	.015	54%	90%
25% reduction	.02	65%	96%
	.025	75%	99%
	.030	82%	99%+
	.01	53%	90%
	.015	70%	98%
30% reduction	.02	82%	99%
	.025	89%	99%+
	.030	94%	99%+

Table 2: Power for CHD
Same dropout and dropin as above. Power assuming the loss rate in the protocol is given.

Treatment Reduction	Control Rate/year	N = 4,000	N = 10,000
	.008	21%	44%
	.010	25%	52%
20% reduction	.012	29%	60%
	.014	33%	66%
	.016	36%	72%
	.008	31%	63%
	.010	37%	73%
25% reduction	.012	43%	80%
	.014	48%	86%
	.016	53%	90%
	.008	42%	80%
	.010	50%	88%
30% reduction	.012	58%	93%
	.014	64%	96%
	.016	70%	98%
	.008	55%	91%
	.010	65%	96%
35% reduction	.012	72%	98%
	.014	79%	99%
	.016	84%	99%+

PROTOCOL ADDENDUM 2
Monitoring Boundaries

When data are examined repeatedly in a trial, the type 1 error rate can be increased dramatically if no account is taken of these multiple "looks" (Armitage, Mc.Pherson and Rowe, 1969; Proschan, Follmann, and Waclawiw, 1992). Boundaries have been constructed in two-armed trials that either eliminate or greatly ameliorate this type 1 error rate inflation. These are called group-sequential rather than sequential boundaries because interim analyses are conducted after groups of data accrue rather than after every new observation. The most popular of these require extremely strong evidence to declare significance very early in the trial, and roughly the same degree of evidence at the end of the trial that would be required had there been no interim looks. This was not the case for the first group-sequential procedure proposed (Pocock, 1977). For this reason, Pocock himself recommends against using his procedure (personal communication).

The O'Brien-Fleming (1979) boundary has the above properties. The drawback is that it requires the number of looks to be specified in advance, and these looks must occur after equal increments of information. In the context of this trial, *information time* t is estimated by the ratio of the number of events observed thus far to the number expected by the end of the trial. Thus t=0 is the beginning and t=1 is the end of the study. Suppose we planned eight looks at the data after equal increments of information (numbers of events). The boundary is given in Figure 1 for the comparison of a given arm with the diuretic. A multiple comparison adjustment has been made for the three comparisons with the diuretic.

Lan and DeMets (1983) proposed a "spending function" approach which allows the data to be examined after different amounts of information, and does not require the number of looks to be specified in advance. The spending function $\alpha^*(t)$ represents the cumulative amount of type 1 error probability that is spent by information time t, with $\alpha^*(1)=\alpha$. The amount of type 1 error probability spent is inextricably linked to information time. This contrasts with the method of Slud and Wei (1982), which spends a fixed amount of type 1 error probability at each look at the data, regardless of the information time. Serious inflation of the type 1 error rate can occur with the Slud and Wei procedure, but not with the Lan-DeMets procedure (Proschan, Follmann, and Waclawiw, 1992). One of the spending functions suggested by Lan and DeMets (see the equation in the Appendix) has boundaries very similar to the O'Brien-Fleming boundary if the looks happen to occur after equal increments of information (see Figure 1). The advantage of the Lan-DeMets approach is that it can be used even when the looks are not equally spaced. In this case the boundaries would change somewhat.

Another extremely useful monitoring tool is conditional power (Lan, Simon, and Halperin, 1982 or Lan and Wittes, 1988). The conditional probability of obtaining a statistically significant result at the end of the trial is computed under different hypothesized treatment effects. Unlike the O'Brien-Fleming or Lan-DeMets boundaries, conditional power is usually used to justify terminating a trial which has no realistic chance of producing a statistically significant result. The trial is stopped if the conditional power is very low even assuming a large treatment benefit for the remainder of the trial. Stochastic curtailment refers to stopping a trial because the conditional power crosses a pre-specified threshold value. For example, one could agree to stop the trial if the conditional power assuming the pre-specified alternative hypothesis is less than or equal to .10. If one uses such a rule, the chance of a type 2 error (accepting the null hypothesis when it is false) is greater than it would be without stochastic curtailment. This is because one could accept the null hypothesis at the end of the study or at an interim point.

The degree of type 2 error rate inflation is quite small. Lan, Simon, and Halperin (1982) showed that it is fairly small even if one monitors the trial continuously. Davis and Hardy (1990) showed that in the more realistic situation in which a trial is monitored 5 to 10 times, the inflation is much smaller.

An important issue that comes up in group sequential monitoring is that of information time. We mentioned above that information time is estimated using the number of events observed thus far divided by the number expected by the end of the trial. But the number in what arms? In two armed monitoring one could either use the total number of events in both arms or the number of events in the control arm. The advantage of using both arms is that it provides a larger sample size to estimate information time. A disadvantage is that in order to estimate the number of events to expect by the end of the trial, one has to project not only a control group event rate, but a treatment effect as well. We have four antihypertensive arms in ALLHAT, hence three treatment effects to specify. It is recommended that we use the diuretic arm events to determine information time.

References

Armitage, P., Mc.Pherson, C.K., and Rowe, B.C. (1969). Repeated significance tests on accumulating data. *Journal of the Royal Statistical Society A.* **132**, 235-244.

Lan, K.K.G. and DeMets, D.L. (1983). Discrete sequential boundaries for clinical trials. *Biometrika* **70**, 659-663.

Lan, K.K.G., Simon, R., and Halperin, M. (1982). Stochastically curtailed tests in long term clinical trials. *Communications in Statistics-Sequential Analysis* **1(3)**, 207-219.

Lan, K.K.G. and Wittes, J. (1988). The B-value: a tool for monitoring data. *Biometrics* **44**, 579-585.

O'Brien, P.C. and Fleming, T.R. (1979). A multiple testing procedure for clinical trials. *Biometrics* **35**, 549-556.

Pocock, S.J. (1977). Group sequential methods in the design and analysis of clinical trials. *Biometrika* **64**, 191-199.

Proschan, M.A., Follmann, D.A., and Waclawiw, M.A. (1992). Effects of assumption violations on type 1 error rate in group sequential monitoring. *Biometrics* **48**, 1131-1143.

Slud, E. and Wei, L.J. (1982). Two sample repeated significance tests based on the modified Wilcoxon statistic. *Journal of the American Statistical Association* **77**, 862-868.

APPENDIX

Recommended Spending Function

$$\alpha^*(t) = 2[1\Phi(z_{\alpha/4} / \sqrt{t})],$$

The Lan-DeMets spending function we recommend is where α is the two-sided type 1 error rate for a given comparison with diuretic, Φ is the standard normal distribution function, and $z_{\alpha/4}$ is its 100(1-α/4)th percentile. When we adjust for multiple comparisons with the diuretic, the value of $z_{\alpha/4}$ becomes approximately 2.64.

PROPOSED STOPPING RULES

1) Use Lan-DeMets version of O'Brien-Fleming for harm/benefit.
2) The boundaries will be symmetric.
3) Information time will be calculated as proportion of expected events in diuretic arm.
4) Take first look at about 10% of information time and then annually at DSMB meetings.
5) Pay special attention at beginning to results that cross the Haybittle-Peto boundary of Z=4.0 for the antihypertensive component, Z=3.0 for the lipid-lowering component.
6) Use conditional power under the protocol specified alternative hypothesis for futility.
