# Supplementary material for: Mortality and Morbidity Among Individuals With Hypertension Receiving a Diuretic, ACE Inhibitor, or Calcium Channel Blocker: A Secondary Analysis of a Randomized Clinical Trial
Source: JAMA Netw Open. 2023 Dec 4;6(12):e2344998. doi: 10.1001/jamanetworkopen.2023.44998 (PMC10696481; doi:10.1001/jamanetworkopen.2023.44998)
Supplement: Supplement 2. — eTable 1. Baseline Characteristics for the Full Sample and by Randomized Group for Morbidity Study at the Time of Randomization eTable 2. Adjusted Hazard Ratios for All-Cause and CVD Mortality With Follow-Up From 2/23/1994 to 12/31/2017 eTable 3. Adjusted Hazard Ratios for Morbidity With Follow-Up From 3/10/1994 to 12/31/2017 eTable 4. Adjusted Hazard Ratios for Non-CVD Mortality With Follow-Up From 2/23/1994 to 12/31/2017 eTable 5. Hazard Ratios Adjusted by Variables Selected Through Penalized Regression for All-Cause and CVD Mortality With Follow-Up From 2/23/1994 to 12/31/2017 eTable 6. Hazard Ratios Adjusted by Variables Selected Through Penalized Regression for Morbidity With Follow-Up From 3/10/1994 to 12/31/2017 eTable 7. Hazard Ratios Adjusted by Variables Selected Through Penalized Regression for Non-CVD Mortality With Follow-Up From 2/23/1994 to 12/31/2017 eFigure 1A. Kaplan-Meier Plots of Primary and Secondary Outcomes Through the Extended Follow-Up (by 12/31/2017) eFigure 1B. Kaplan-Meier Plots of Primary and Secondary Outcomes Through the Extended Follow-Up (by 12/31/2017) eFigure 2A. Unadjusted Hazard Ratios (95% CI) and Forest Plots of Overall and Subgroup Analyses of Mortality and Morbidity Outcomes Through the Extended Follow-Up (by 12/31/2017) – Amlodipine vs. Chlorthalidone and Lisinopril vs. Chlorthalidone eFigure 2B. Unadjusted Hazard Ratios (95% CI) and Forest Plots of Overall and Subgroup Analyses of Mortality and Morbidity Outcomes Through the Extended Follow-Up (by 12/31/2017) – Amlodipine vs. Chlorthalidone and Lisinopril vs. Chlorthalidone eFigure 2C. Unadjusted Hazard Ratios (95% CI) and Forest Plots of Overall and Subgroup Analyses of Mortality and Morbidity Outcomes Through the Extended Follow-Up (by 12/31/2017) – Amlodipine vs. Chlorthalidone and Lisinopril vs. Chlorthalidone [file jamanetwopen-e2344998-s002.pdf]

Supplementary Online Content

Yamal JM, Martinez J, Osani MC, Du XL, Simpson LM, Davis BR. Mortality and morbidity among individuals with hypertension receiving a diuretic, ACE inhibitor, or calcium channel blocker: a secondary analysis of a randomized clinical trial. *JAMA Netw Open.* 2023;6(12):e2344998. doi:10.1001/jamanetworkopen.2023.44998

eTable 1. Baseline Characteristics for the Full Sample and by Randomized Group for Morbidity Study at the Time of Randomization
eTable 2. Adjusted Hazard Ratios for All-Cause and CVD Mortality With Follow-Up From 2/23/1994 to 12/31/2017
eTable 3. Adjusted Hazard Ratios for Morbidity With Follow-Up From 3/10/1994 to 12/31/2017
eTable 4. Adjusted Hazard Ratios for Non-CVD Mortality With Follow-Up From 2/23/1994 to 12/31/2017
eTable 5. Hazard Ratios Adjusted by Variables Selected Through Penalized Regression for All-Cause and CVD Mortality With Follow-Up From 2/23/1994 to 12/31/2017
eTable 6. Hazard Ratios Adjusted by Variables Selected Through Penalized Regression for Morbidity With Follow-Up From 3/10/1994 to 12/31/2017
eTable 7. Hazard Ratios Adjusted by Variables Selected Through Penalized Regression for Non-CVD Mortality With Follow-Up From 2/23/1994 to 12/31/2017
eFigure 1A. Kaplan-Meier Plots of Primary and Secondary Outcomes Through the Extended Follow-Up (by 12/31/2017)
eFigure 1B. Kaplan-Meier Plots of Primary and Secondary Outcomes Through the Extended Follow-Up (by 12/31/2017)
eFigure 2A. Unadjusted Hazard Ratios (95% CI) and Forest Plots of Overall and Subgroup Analyses of Mortality and Morbidity Outcomes Through the Extended Follow-Up (by 12/31/2017) – Amlodipine vs. Chlorthalidone and Lisinopril vs. Chlorthalidone
eFigure 2B. Unadjusted Hazard Ratios (95% CI) and Forest Plots of Overall and Subgroup Analyses of Mortality and Morbidity Outcomes Through the Extended Follow-Up (by 12/31/2017) – Amlodipine vs. Chlorthalidone and Lisinopril vs. Chlorthalidone
eFigure 2C. Unadjusted Hazard Ratios (95% CI) and Forest Plots of Overall and Subgroup Analyses of Mortality and Morbidity Outcomes Through the Extended Follow-Up (by 12/31/2017) – Amlodipine vs. Chlorthalidone and Lisinopril vs. Chlorthalidone

This supplementary material has been provided by the authors to give readers additional information about their work.

© 2023 Yamal JM et al. *JAMA Network Open.*

eTable 1. Baseline Characteristics for the Full Sample and by Randomized Group for Morbidity Study at the Time of Randomization

	Participants, No. (%) (Unless otherwise indicated)				
Characteristic	**Full Sample**	**Chlorthalidone**	**Amlodipine**	**Lisinopril**	**P value[a]**
**At the recruitment in ALLHAT**					
See previous references [2]					
**Eligible for further follow-up as of 3/31/2002**	22754	10414	6191	6149	
**Age, mean (SD), years**	68.72 (7.2)	68.74 (7.2)	68.69 (7.2)	68.74 (7.3)	0.914
**Age group at trial baseline**					
Age <65	7516 (33.0)	3411 (32.8)	2062 (33.3)	2043 (33.2)	0.712
Age ≥65	15238 (67.0)	7003 (67.2)	4129 (66.7)	4106 (66.8)	
**Gender**					
Male	9982 (43.9)	4570 (43.9)	2649 (42.8)	2763 (44.9)	0.056
Female	12772 (56.1)	5844 (56.1)	3542 (57.2)	3386 (55.1)	
**Race/Ethnicity**					
Black	8199 (36.0)	3748 (36.0)	2248 (36.3)	2203 (35.8)	0.848
Non-Black	14555 (64.0)	6666 (64.0)	3943 (63.7)	3946 (64.2)	
**Hispanic/Latino Ethnicity**					
Hispanic	4872 (21.5)	2228 (21.5)	1294 (21.0)	1350 (22.1)	0.352
Non-Hispanic	17756 (78.5)	8129 (78.5)	4864 (79.0)	4763 (77.9)	
**Education, mean (SD), years**	10.61 (4.2)	10.63 (4.1)	10.61 (4.1)	10.58 (4.3)	0.747
**Education level at trial baseline**					
High school or less	15560 (74.1)	7088 (73.6)	4255 (74.3)	4217 (74.5)	0.408
More than high school	5448 (25.9)	2538 (26.4)	1469 (25.7)	1441 (25.5)	
**Treatment with antihypertensive drugs prior to trial baseline**					
Treated	20586 (90.5)	9403 (90.3)	5618 (90.7)	5565 (90.5)	0.639
Untreated	2167 (9.5)	1010 (9.7)	573 (9.3)	584 (9.5)	
**Aspirin use at trial baseline**	7828 (34.9)	3573 (34.8)	2110 (34.5)	2145 (35.4)	0.575
**Women taking estrogen at trial baseline**	1987 (15.8)	918 (16.0)	557 (16.0)	512 (15.4)	0.739
**Mean Glomerular Filtration Rate (GFR) at trial baseline, mL/min/1.73 m^2**	76.14 (19.8)	76.03 (19.9)	76.36 (19.7)	76.10 (19.8)	0.582
**GFR <60 mL/min/1.73 m^2 at trial baseline**	4377 (20.2)	2019 (20.3)	1185 (20.1)	1173 (20.1)	0.924
**HDL cholesterol at trial baseline, mean (SD), mg/dl**	47.83 (14.9)	47.79 (15.0)	48.20 (15.0)	47.54 (14.6)	0.050
**HDL <35 mg/dl at trial baseline**	2373 (10.4)	1083 (10.4)	646 (10.4)	644 (10.5)	0.989
**Cigarette smoking at trial baseline**					
Never smoker	9701 (42.6)	4421 (42.5)	2682 (43.3)	2598 (42.3)	0.630
Current Smoker	4301 (18.9)	1967 (18.9)	1178 (19.0)	1156 (18.8)	
Former Smoker	8751 (38.5)	4026 (38.7)	2331 (37.7)	2394 (38.9)	
**Diabetes classification at trial baseline**					
Diabetes	9133 (43.4)	4187 (43.5)	2499 (43.7)	2447 (42.9)	0.694
Non-diabetes	11922 (56.6)	5443 (56.5)	3224 (56.3)	3255 (57.1)	
**History of Coronary Heart Disease (CHD) at trial baseline**	5743 (25.4)	2685 (26.0)	1511 (24.6)	1547 (25.3)	0.142

© 2023 Yamal JM et al. *JAMA Network Open.*

**Atherosclerotic Cardiovascular Disease (ASCVD) at trial baseline**	12120 (53.3)	5597 (53.7)	3231 (52.2)	3292 (53.5)	0.133
**History of myocardial infarction (MI) or stroke at trial baseline**	5231 (23.0)	2424 (23.3)	1433 (23.1)	1374 (22.3)	0.366
**History of coronary artery bypass graft (CABG) at trial baseline**	2967 (13.0)	1380 (13.3)	746 (12.0)	841 (13.7)	**0.019**
**Other ASCVD at trial baseline**	5774 (25.4)	2637 (25.3)	1556 (25.1)	1581 (25.7)	0.750
**Major ST segment depression at trial baseline**	2394 (10.6)	1117 (10.8)	621 (10.1)	656 (10.8)	0.335
**Hard Left ventricular hypertrophy (LVH) by Minnesota code at trial baseline**	1016 (5.3)	459 (5.2)	283 (5.4)	274 (5.3)	0.864
**Lipid Lowering Trial (LLT) Participants**	5542 (24.4)	2569 (24.7)	1516 (24.5)	1457 (23.7)	0.355
**BMI, mean (SD), kg/m^2 at trial baseline**	29.64 (6.2)	29.57 (6.2)	29.69 (6.2)	29.69 (6.2)	0.377
**Obesity (BMI ≥ 30 kg/m^2) at trial baseline**	9381 (41.2)	4266 (41.0)	2566 (41.4)	2549 (41.5)	0.759
**Baseline blood pressure (BP) reading, mmHg**					
Systolic BP, mean (SD)	146.08 (13.1)	146.07 (13.0)	145.90 (13.2)	146.30 (13.1)	0.234
Diastolic BP, mean (SD)	83.45 (9.0)	83.50 (9.0)	83.30 (9.1)	83.51 (9.0)	0.320
**Blood pressure change from baseline to the latest BP reading prior to 3/31/2002, mmHg**					
Systolic BP, mean (SD)	-9.90 (19.7)	-10.54 (19.3)	-9.97 (19.3)	-8.73 (20.5)	**<0.001**
Diastolic BP, mean (SD)	-6.49 (11.5)	-6.56 (11.4)	-6.81 (11.4)	-6.06 (11.9)	**<0.001**

N= number of participants; SD= standard deviation

[a] P values represent significance level of the chi-square test of independence between randomized groups for binary and categorical variables, or the one-way analysis of variance (ANOVA) between randomized groups for continuous variables. Statistically significant p values (<0.05) are shown in bold text.

Conversion factors: 1 mL/min/1.73m^2 = 0.0167 mL/s/m^2; 1 mg/dl = 0.0259 mmol/L; 1 mmHg = 0.133 kPa

© 2023 Yamal JM et al. *JAMA Network Open.*

eTable 2. Adjusted[a] Hazard Ratios for All-Cause and CVD Mortality With Follow-up From 2/23/1994[b] to 12/31/2017

	All-Cause Mortality		CVD Mortality		CHD Mortality		Stroke Mortality		CHF Mortality		Other CVD Mortality	
	**Adjusted HR (95% CI)**	**P value**	**Adjusted HR (95% CI)**	**P value**	**Adjusted HR (95% CI)**	**P value**	**Adjusted HR (95% CI)**	**P value**	**Adjusted HR (95% CI)**	**P value**	**Adjusted HR (95% CI)**	**P value**
**Randomized group**												
Amlodipine vs. Chlorthalidone	1.00 (0.96-1.03)	0.928	0.97 (0.89-1.05)	0.434	1.01 (0.94-1.10)	0.751	1.00 (0.86-1.16)	0.995	1.01 (0.85-1.21)	0.904	1.00 (0.91-1.11)	0.950
Lisinopril vs Chlorthalidone	1.01 (0.97-1.04)	0.762	1.06 (0.97-1.15)	0.174	0.97 (0.90-1.06)	0.518	**1.19 (1.03-1.37)**	**0.017**	1.03 (0.86-1.22)	0.776	0.95 (0.85-1.05)	0.299
**Age group at trial baseline**												
Age <65	1.00 (reference)		1.00 (reference)		1.00 (reference)		1.00 (reference)		1.00 (reference)		1.00 (reference)	
Age ≥65	2.09 (2.02-2.16)	<0.001	2.24 (2.06-2.43)	<0.001	1.96 (1.82-2.11)	<0.001	2.41 (2.09-2.78)	<0.001	2.96 (2.47-3.54)	<0.001	2.37 (2.14-2.61)	<0.001
**Gender**												
Male	1.00 (reference)		1.00 (reference)		1.00 (reference)		1.00 (reference)		1.00 (reference)		1.00 (reference)	
Female	0.78 (0.75-0.80)	<0.001	0.82 (0.76-0.89)	<0.001	0.71 (0.65-0.76)	<0.001	0.87 (0.76-1.00)	0.054	0.89 (0.76-1.05)	0.170	0.78 (0.71-0.86)	<0.001
**Race/Ethnicity**												
Non-Black	1.00 (reference)		1.00 (reference)		1.00 (reference)		1.00 (reference)		1.00 (reference)		1.00 (reference)	
Black	0.98 (0.95-1.02)	0.306	0.95 (0.87-1.03)	0.211	0.95 (0.88-1.03)	0.184	0.95 (0.82-1.09)	0.477	0.94 (0.79-1.12)	0.514	1.21 (1.10-1.33)	<0.001
**Hispanic/Latino Ethnicity**												
Non-Hispanic	1.00 (reference)		1.00 (reference)		1.00 (reference)		1.00 (reference)		1.00 (reference)		1.00 (reference)	
Hispanic	0.79 (0.76-0.83)	<0.001	0.86 (0.77-0.95)	0.004	0.78 (0.71-0.87)	<0.001	0.76 (0.63-0.91)	0.003	0.70 (0.55-0.88)	0.003	0.70 (0.61-0.80)	<0.001
**Education level at trial baseline**												
More than high school	1.00 (reference)		1.00 (reference)		1.00 (reference)		1.00 (reference)		1.00 (reference)		1.00 (reference)	
High school or less	1.17 (1.13-1.21)	<0.001	1.19 (1.10-1.30)	<0.001	1.22 (1.13-1.32)	<0.001	1.11 (0.97-1.27)	0.145	1.16 (0.98-1.38)	0.078	1.04 (0.94-1.14)	0.467
**Treatment with antihypertensive drugs prior to trial baseline**												
Untreated	1.00 (reference)		1.00 (reference)		1.00 (reference)		1.00 (reference)		1.00 (reference)		1.00 (reference)	
Treated	1.10 (1.04-1.16)	<0.001	1.13 (0.99-1.30)	0.061	1.09 (0.97-1.23)	0.163	1.34 (1.06-1.70)	0.014	0.98 (0.75-1.30)	0.902	1.05 (0.91-1.23)	0.493
**Aspirin use at trial baseline**												
No	1.00 (reference)		1.00 (reference)		1.00 (reference)		1.00 (reference)		1.00 (reference)		1.00 (reference)	
Yes	0.99 (0.96-1.03)	0.629	1.05 (0.97-1.14)	0.231	0.98 (0.91-1.06)	0.595	1.14 (0.99-1.30)	0.062	0.97 (0.83-1.15)	0.758	1.07 (0.97-1.18)	0.150
**GFR <60 mL/min/1.73 m² at trial baseline**												
No	1.00 (reference)		1.00 (reference)		1.00 (reference)		1.00 (reference)		1.00 (reference)		1.00 (reference)	
Yes	1.50 (1.44-1.55)	<0.001	1.60 (1.46-1.74)	<0.001	1.48 (1.37-1.61)	<0.001	1.60 (1.38-1.85)	<0.001	1.72 (1.44-2.04)	<0.001	1.62 (1.46-1.81)	<0.001
**HDL cholesterol <35 mg/dl at trial baseline**												
No	1.00 (reference)		1.00 (reference)		1.00 (reference)		1.00 (reference)		1.00 (reference)		1.00 (reference)	
Yes	0.90 (0.86-0.95)	<0.001	0.86 (0.77-0.97)	0.012	1.01 (0.91-1.11)	0.920	0.70 (0.57-0.87)	0.001	0.83 (0.65-1.05)	0.126	0.84 (0.73-0.97)	0.018
**Cigarette smoking at trial baseline**												

© 2023 Yamal JM et al. *JAMA Network Open.*

	All-Cause Mortality		CVD Mortality		CHD Mortality		Stroke Mortality		CHF Mortality		Other CVD Mortality	
	Adjusted HR (95% CI)	P value	Adjusted HR (95% CI)	P value	Adjusted HR (95% CI)	P value	Adjusted HR (95% CI)	P value	Adjusted HR (95% CI)	P value	Adjusted HR (95% CI)	P value
Never smoker	1.00 (reference)		1.00 (reference)		1.00 (reference)		1.00 (reference)		1.00 (reference)		1.00 (reference)	
Current smoker	1.69 (1.62-1.77)	<0.001	1.31 (1.18-1.46)	<0.001	1.49 (1.35-1.65)	<0.001	1.11 (0.93-1.34)	0.248	1.19 (0.94-1.50)	0.142	1.48 (1.31-1.68)	<0.001
Former Smoker	1.11 (1.07-1.15)	<0.001	1.04 (0.96-1.13)	0.313	1.12 (1.04-1.22)	0.004	0.95 (0.82-1.10)	0.479	0.98 (0.83-1.17)	0.853	1.07 (0.96-1.18)	0.206
**Diabetes classification at trial baseline**												
Non-diabetes	1.00 (reference)		1.00 (reference)		1.00 (reference)		1.00 (reference)		1.00 (reference)		1.00 (reference)	
Diabetes	1.53 (1.48-1.58)	<0.001	1.65 (1.53-1.78)	<0.001	1.69 (1.57-1.81)	<0.001	1.45 (1.26-1.65)	<0.001	1.76 (1.50-2.06)	<0.001	1.28 (1.17-1.41)	<0.001
**History of Coronary Heart Disease (CHD) at trial baseline**												
No	1.00 (reference)		1.00 (reference)		1.00 (reference)		1.00 (reference)		1.00 (reference)		1.00 (reference)	
Yes	0.96 (0.92-1.01)	0.085	0.91 (0.82-1.01)	0.088	1.08 (0.98-1.19)	0.122	0.85 (0.71-1.02)	0.080	1.03 (0.83-1.28)	0.791	0.88 (0.77-1.01)	0.062
**Atherosclerotic Cardiovascular Disease (ASCVD) at trial baseline**												
No	1.00 (reference)		1.00 (reference)		1.00 (reference)		1.00 (reference)		1.00 (reference)		1.00 (reference)	
Yes	1.09 (1.03-1.16)	0.002	1.23 (1.08-1.40)	0.002	1.14 (1.01-1.29)	0.030	1.04 (0.83-1.31)	0.734	1.50 (1.14-1.97)	0.004	1.15 (0.97-1.35)	0.103
**History of myocardial infarction (MI) or stroke at trial baseline**												
No	1.00 (reference)		1.00 (reference)		1.00 (reference)		1.00 (reference)		1.00 (reference)		1.00 (reference)	
Yes	1.28 (1.22-1.34)	<0.001	1.43 (1.28-1.59)	<0.001	1.49 (1.35-1.64)	<0.001	1.54 (1.27-1.87)	<0.001	1.04 (0.83-1.30)	0.736	1.30 (1.13-1.50)	<0.001
**History of coronary artery bypass graft (CABG) at trial baseline**												
No	1.00 (reference)		1.00 (reference)		1.00 (reference)		1.00 (reference)		1.00 (reference)		1.00 (reference)	
Yes	1.11 (1.05-1.17)	<0.001	1.20 (1.07-1.35)	0.002	1.41 (1.28-1.57)	<0.001	0.97 (0.78-1.19)	0.748	1.20 (0.94-1.54)	0.141	1.03 (0.88-1.20)	0.732
**Other ASCVD at trial baseline**												
No	1.00 (reference)		1.00 (reference)		1.00 (reference)		1.00 (reference)		1.00 (reference)		1.00 (reference)	
Yes	1.15 (1.09-1.20)	<0.001	1.13 (1.02-1.26)	0.020	1.19 (1.09-1.31)	<0.001	1.31 (1.08-1.57)	0.005	0.90 (0.72-1.13)	0.363	1.06 (0.92-1.21)	0.433
**Major ST segment depression at trial baseline**												
No	1.00 (reference)		1.00 (reference)		1.00 (reference)		1.00 (reference)		1.00 (reference)		1.00 (reference)	
Yes	0.97 (0.92-1.03)	0.335	0.95 (0.84-1.09)	0.479	0.98 (0.87-1.10)	0.704	0.95 (0.76-1.19)	0.660	0.89 (0.68-1.17)	0.412	0.91 (0.77-1.07)	0.247
**LVH by Minnesota code at trial baseline**												
No/Soft LVH	1.00 (reference)		1.00 (reference)		1.00 (reference)		1.00 (reference)		1.00 (reference)		1.00 (reference)	
Hard LVH	1.33 (1.25-1.42)	<0.001	1.68 (1.46-1.92)	<0.001	1.53 (1.34-1.75)	<0.001	1.73 (1.37-2.18)	<0.001	1.89 (1.44-2.49)	<0.001	1.63 (1.38-1.93)	<0.001
**Lipid Lowering Trial (LLT) Participant**												
No	1.00 (reference)		1.00 (reference)		1.00 (reference)		1.00 (reference)		1.00 (reference)		1.00 (reference)	
Yes	0.93 (0.90-0.97)	<0.001	0.95 (0.88-1.03)	0.238	0.98 (0.91-1.06)	0.605	0.99 (0.86-1.14)	0.863	0.85 (0.71-1.02)	0.077	1.01 (0.92-1.11)	0.844
**Obesity (BMI ≥ 30 kg/m²) at trial baseline**												
No	1.00 (reference)		1.00 (reference)		1.00 (reference)		1.00 (reference)		1.00 (reference)		1.00 (reference)	
Yes	0.96 (0.93-0.99)	0.008	0.99 (0.92-1.06)	0.753	0.99 (0.93-1.06)	0.862	0.86 (0.76-0.98)	0.025	1.15 (0.99-1.34)	0.064	0.98 (0.89-1.07)	0.599
**Baseline blood pressure reading, per 10 mmHg**												
Systolic BP	1.11 (1.09-1.12)	<0.001	1.12 (1.08-1.15)	<0.001	1.15 (1.11-1.18)	<0.001	1.13 (1.07-1.20)	<0.001	1.10 (1.03-1.17)	0.005	1.11 (1.07-1.16)	<0.001

© 2023 Yamal JM et al. *JAMA Network Open.*

	All-Cause Mortality		CVD Mortality		CHD Mortality		Stroke Mortality		CHF Mortality		Other CVD Mortality	
	Adjusted HR (95% CI)	P value	Adjusted HR (95% CI)	P value	Adjusted HR (95% CI)	P value	Adjusted HR (95% CI)	P value	Adjusted HR (95% CI)	P value	Adjusted HR (95% CI)	P value
Diastolic BP	0.88 (0.86-0.90)	<0.001	0.90 (0.86-0.94)	<0.001	0.87 (0.84-0.91)	<0.001	0.99 (0.91-1.07)	0.759	0.78 (0.71-0.86)	<0.001	0.95 (0.90-1.01)	0.079

HR= Hazard ratio; 95% CI= 95% Confidence interval; CHD= coronary heart disease; CVD= cardiovascular disease; ASCVD= Atherosclerotic Cardiovascular Disease; OASCVD= Other ASCVD; HDL= high density lipoprotein; eGFR= estimated glomerular filtration rate; LVH= left ventricular hypertrophy; SBP= systolic blood pressure; DBP= diastolic blood pressure

[a] Adjusted for each covariate shown in that column. Covariates not included in multivariable analysis are indicated with dashes.

[b] First randomization date in the Mortality cohort.

Conversion factors: 1 mL/min/1.73m^2 = 0.0167 mL/s/m^2; 1 mg/dl = 0.0259 mmol/L; 1 mmHg = 0.133 kPa

© 2023 Yamal JM et al. *JAMA Network Open.*

eTable 3. Adjusted[a] Hazard Ratios for Morbidity With Follow-up From 3/10/1994[b] to 12/31/2017

	CVD Morbidity		CHD Morbidity		CHF Morbidity		Stroke Morbidity		Cancer Morbidity		Kidney Disease Morbidity	
	**Adjusted HR (95% CI)**	**P value**	**Adjusted HR (95% CI)**	**P value**	**Adjusted HR (95% CI)**	**P value**	**Adjusted HR (95% CI)**	**P value**	**Adjusted HR (95% CI)**	**P value**	**Adjusted HR (95% CI)**	**P value**
**Randomized group**												
Amlodipine vs. Chlorthalidone	1.01 (0.96-1.06)	0.628	0.99 (0.92-1.06)	0.795	1.02 (0.97-1.09)	0.426	1.04 (0.96-1.12)	0.389	0.99 (0.92-1.07)	0.842	1.05 (0.90-1.22)	0.535
Lisinopril vs Chlorthalidone	1.04 (0.99-1.09)	0.157	0.98 (0.91-1.05)	0.508	1.03 (0.97-1.09)	0.412	**1.11 (1.03-1.20)**	**0.008**	1.02 (0.94-1.10)	0.632	1.12 (0.97-1.30)	0.117
**Age group at trial baseline**												
Age <65	1.00 (reference)		1.00 (reference)		1.00 (reference)		1.00 (reference)		1.00 (reference)		1.00 (reference)	
Age ≥65	1.76 (1.67-1.84)	<0.001	1.66 (1.55-1.78)	<0.001	1.84 (1.74-1.95)	<0.001	1.77 (1.63-1.91)	<0.001	1.63 (1.51-1.76)	<0.001	1.02 (0.89-1.17)	0.807
**Gender**												
Male	1.00 (reference)		1.00 (reference)		1.00 (reference)		1.00 (reference)		1.00 (reference)		1.00 (reference)	
Female	0.91 (0.87-0.96)	<0.001	0.80 (0.75-0.86)	<0.001	0.96 (0.91-1.02)	0.151	0.94 (0.88-1.01)	0.117	0.69 (0.64-0.74)	<0.001	0.63 (0.55-0.73)	<0.001
**Race/Ethnicity**												
Non-Black	1.00 (reference)		1.00 (reference)		1.00 (reference)		1.00 (reference)		1.00 (reference)		1.00 (reference)	
Black	0.97 (0.93-1.02)	0.265	0.90 (0.84-0.97)	0.006	0.99 (0.93-1.04)	0.630	1.03 (0.95-1.11)	0.481	1.05 (0.97-1.13)	0.214	1.92 (1.67-2.21)	<0.001
**Hispanic/Latino Ethnicity**												
Non-Hispanic	1.00 (reference)		1.00 (reference)		1.00 (reference)		1.00 (reference)		1.00 (reference)		1.00 (reference)	
Hispanic	0.72 (0.68-0.77)	<0.001	0.80 (0.73-0.87)	<0.001	0.63 (0.59-0.68)	<0.001	0.76 (0.69-0.84)	<0.001	0.63 (0.56-0.69)	<0.001	1.02 (0.85-1.22)	0.836
**Education level at trial baseline**												
More than high school	1.00 (reference)		1.00 (reference)		1.00 (reference)		1.00 (reference)		1.00 (reference)		1.00 (reference)	
High school or less	1.19 (1.13-1.25)	<0.001	1.20 (1.12-1.29)	<0.001	1.19 (1.12-1.26)	<0.001	1.14 (1.06-1.23)	<0.001	1.12 (1.04-1.21)	0.003	1.06 (0.92-1.23)	0.407
**Treatment with antihypertensive drugs prior to trial baseline**												
Untreated	1.00 (reference)		1.00 (reference)		1.00 (reference)		1.00 (reference)		1.00 (reference)		1.00 (reference)	
Treated	1.27 (1.17-1.38)	<0.001	1.09 (0.97-1.21)	0.132	1.23 (1.12-1.35)	<0.001	1.36 (1.19-1.54)	<0.001	1.08 (0.96-1.22)	0.184	1.29 (1.02-1.63)	0.031
**Aspirin use at trial baseline**												
No	1.00 (reference)		1.00 (reference)		1.00 (reference)		1.00 (reference)		1.00 (reference)		1.00 (reference)	
Yes	1.06 (1.01-1.11)	0.018	1.05 (0.98-1.12)	0.151	1.02 (0.96-1.08)	0.490	1.15 (1.07-1.23)	<0.001	0.91 (0.84-0.98)	0.013	0.90 (0.78-1.04)	0.141
**GFR <60 mL/min/1.73 m^2 at trial baseline**												
No	1.00 (reference)		1.00 (reference)		1.00 (reference)		1.00 (reference)		1.00 (reference)		1.00 (reference)	
Yes	1.42 (1.34-1.49)	<0.001	1.42 (1.32-1.53)	<0.001	1.55 (1.46-1.65)	<0.001	1.31 (1.21-1.42)	<0.001	1.19 (1.09-1.29)	<0.001	5.30 (4.66-6.04)	<0.001
**HDL cholesterol <35 mg/dl at trial baseline**												
No	1.00 (reference)		1.00 (reference)		1.00 (reference)		1.00 (reference)		1.00 (reference)		1.00 (reference)	
Yes	0.96 (0.90-1.03)	0.260	1.09 (0.99-1.19)	0.078	0.94 (0.87-1.02)	0.160	0.89 (0.80-0.99)	0.040	1.05 (0.95-1.16)	0.354	1.01 (0.82-1.25)	0.920
**Cigarette smoking at trial baseline**												

© 2023 Yamal JM et al. *JAMA Network Open.*

	CVD Morbidity		CHD Morbidity		CHF Morbidity		Stroke Morbidity		Cancer Morbidity		Kidney Disease Morbidity	
	Adjusted HR (95% CI)	P value	Adjusted HR (95% CI)	P value	Adjusted HR (95% CI)	P value	Adjusted HR (95% CI)	P value	Adjusted HR (95% CI)	P value	Adjusted HR (95% CI)	P value
Never smoker	1.00 (reference)		1.00 (reference)		1.00 (reference)		1.00 (reference)		1.00 (reference)		1.00 (reference)	
Current smoker	1.42 (1.33-1.51)	<0.001	1.50 (1.37-1.64)	<0.001	1.40 (1.30-1.51)	<0.001	1.42 (1.29-1.57)	<0.001	2.08 (1.90-2.28)	<0.001	1.79 (1.49-2.14)	<0.001
Former Smoker	1.09 (1.04-1.15)	<0.001	1.13 (1.05-1.21)	<0.001	1.08 (1.02-1.14)	0.009	1.11 (1.03-1.20)	0.008	1.24 (1.15-1.35)	<0.001	1.23 (1.07-1.43)	0.004
**Diabetes classification at trial baseline**												
Non-diabetes	1.00 (reference)		1.00 (reference)		1.00 (reference)		1.00 (reference)		1.00 (reference)		1.00 (reference)	
Diabetes	1.57 (1.50-1.64)	<0.001	1.70 (1.59-1.81)	<0.001	1.69 (1.60-1.79)	<0.001	1.49 (1.38-1.60)	<0.001	1.11 (1.03-1.20)	0.004	3.01 (2.62-3.45)	<0.001
**History of Coronary Heart Disease (CHD) at trial baseline**												
No	1.00 (reference)		1.00 (reference)		1.00 (reference)		1.00 (reference)		1.00 (reference)		1.00 (reference)	
Yes	0.97 (0.91-1.03)	0.283	1.08 (0.99-1.18)	0.074	1.06 (0.98-1.14)	0.121	0.84 (0.76-0.93)	<0.001	0.96 (0.87-1.07)	0.464	0.86 (0.71-1.05)	0.147
**Atherosclerotic Cardiovascular Disease (ASCVD) at trial baseline**												
No	1.00 (reference)		1.00 (reference)		1.00 (reference)		1.00 (reference)		1.00 (reference)		1.00 (reference)	
Yes	1.13 (1.05-1.23)	0.002	1.13 (1.01-1.26)	0.030	1.11 (1.01-1.22)	0.025	1.13 (1.00-1.27)	0.050	0.98 (0.87-1.11)	0.772	1.00 (0.79-1.27)	0.996
**History of myocardial infarction (MI) or stroke at trial baseline**												
No	1.00 (reference)		1.00 (reference)		1.00 (reference)		1.00 (reference)		1.00 (reference)		1.00 (reference)	
Yes	1.39 (1.30-1.48)	<0.001	1.40 (1.29-1.53)	<0.001	1.36 (1.26-1.46)	<0.001	1.39 (1.25-1.53)	<0.001	1.08 (0.97-1.20)	0.142	1.22 (0.99-1.49)	0.060
**History of coronary artery bypass graft (CABG) at trial baseline**												
No	1.00 (reference)		1.00 (reference)		1.00 (reference)		1.00 (reference)		1.00 (reference)		1.00 (reference)	
Yes	1.26 (1.18-1.36)	<0.001	1.41 (1.28-1.55)	<0.001	1.24 (1.14-1.35)	<0.001	1.32 (1.18-1.47)	<0.001	1.05 (0.93-1.18)	0.451	1.36 (1.08-1.71)	0.009
**Other ASCVD at trial baseline**												
No	1.00 (reference)		1.00 (reference)		1.00 (reference)		1.00 (reference)		1.00 (reference)		1.00 (reference)	
Yes	1.12 (1.05-1.19)	<0.001	1.14 (1.05-1.24)	0.003	1.10 (1.03-1.19)	0.008	1.16 (1.05-1.28)	0.003	1.18 (1.06-1.31)	0.002	1.11 (0.90-1.35)	0.329
**Major ST segment depression at trial baseline**												
No	1.00 (reference)		1.00 (reference)		1.00 (reference)		1.00 (reference)		1.00 (reference)		1.00 (reference)	
Yes	1.03 (0.95-1.11)	0.433	0.95 (0.85-1.06)	0.358	1.11 (1.02-1.22)	0.018	0.94 (0.83-1.06)	0.336	1.09 (0.96-1.23)	0.176	1.17 (0.93-1.48)	0.188
**LVH by Minnesota code at trial baseline**												
No/Soft LVH	1.00 (reference)		1.00 (reference)		1.00 (reference)		1.00 (reference)		1.00 (reference)		1.00 (reference)	
Hard LVH	1.55 (1.42-1.69)	<0.001	1.46 (1.28-1.65)	<0.001	1.73 (1.57-1.91)	<0.001	1.48 (1.30-1.69)	<0.001	0.99 (0.85-1.16)	0.924	1.35 (1.07-1.72)	0.013
**Lipid Lowering Trial (LLT) Participant**												
No	1.00 (reference)		1.00 (reference)		1.00 (reference)		1.00 (reference)		1.00 (reference)		1.00 (reference)	
Yes	0.94 (0.90-0.99)	0.020	0.94 (0.87-1.01)	0.080	0.94 (0.89-1.00)	0.038	0.95 (0.88-1.02)	0.162	0.93 (0.86-1.00)	0.063	1.00 (0.87-1.15)	0.986
**Obesity (BMI ≥ 30 kg/m²) at trial baseline**												
No	1.00 (reference)		1.00 (reference)		1.00 (reference)		1.00 (reference)		1.00 (reference)		1.00 (reference)	
Yes	1.09 (1.05-1.14)	<0.001	0.94 (0.88-1.00)	0.054	1.25 (1.19-1.32)	<0.001	0.94 (0.88-1.01)	0.106	1.01 (0.94-1.08)	0.881	1.19 (1.04-1.35)	0.009
**Baseline blood pressure reading, per 10 mmHg**												
Systolic BP	1.11 (1.09-1.14)	<0.001	1.13 (1.10-1.16)	<0.001	1.12 (1.10-1.15)	<0.001	1.16 (1.13-1.20)	<0.001	1.02 (0.99-1.05)	0.269	1.32 (1.24-1.39)	<0.001

© 2023 Yamal JM et al. *JAMA Network Open.*

	CVD Morbidity		CHD Morbidity		CHF Morbidity		Stroke Morbidity		Cancer Morbidity		Kidney Disease Morbidity	
	**Adjusted HR (95% CI)**	**P value**	**Adjusted HR (95% CI)**	**P value**	**Adjusted HR (95% CI)**	**P value**	**Adjusted HR (95% CI)**	**P value**	**Adjusted HR (95% CI)**	**P value**	**Adjusted HR (95% CI)**	**P value**
Diastolic BP	0.89 (0.86-0.91)	<0.001	0.91 (0.88-0.95)	<0.001	0.86 (0.83-0.89)	<0.001	0.91 (0.87-0.95)	<0.001	0.92 (0.88-0.96)	<0.001	0.89 (0.82-0.96)	0.005

HR= Hazard ratio; 95% CI= 95% Confidence interval; CHD= coronary heart disease; CVD= cardiovascular disease; ASCVD= Atherosclerotic Cardiovascular Disease; OASCVD= Other ASCVD; HDL= high density lipoprotein; eGFR= estimated glomerular filtration rate; LVH= left ventricular hypertrophy; SBP= systolic blood pressure; DBP= diastolic blood pressure

[a] Adjusted for each covariate shown in that column. Covariates not included in multivariable analysis are indicated with dashes.

[b] First randomization date in the full Morbidity cohort.

Conversion factors: 1 mL/min/1.73m^2 = 0.0167 mL/s/m^2; 1 mg/dl = 0.0259 mmol/L; 1 mmHg = 0.133 kPa

© 2023 Yamal JM et al. *JAMA Network Open.*

eTable 4. Adjusted[a] Hazard Ratios for Non-CVD Mortality With Follow-up From 2/23/1994[b] to 12/31/2017

	All Non-CVD Mortality		Cancer Mortality		Kidney Disease Mortality		Mortality Accident/Suicide		Other Non-CVD Mortality		Mortality Unknown Cause	
	**Adjusted* HR (95% CI)**	**P value**	**Adjusted* HR (95% CI)**	**P value**	**Adjusted* HR (95% CI)**	**P value**	**Adjusted* HR (95% CI)**	**P value**	**Adjusted* HR (95% CI)**	**P value**	**Adjusted* HR (95% CI)**	**P value**
**Randomized group**												
Amlodipine vs. Chlorthalidone	0.99 (0.95-1.04)	0.722	0.96 (0.88-1.04)	0.334	1.00 (0.81-1.23)	0.995	0.86 (0.67-1.09)	0.219	1.02 (0.96-1.08)	0.590	1.01 (0.74-1.38)	0.940
Lisinopril vs Chlorthalidone	1.01 (0.96-1.06)	0.651	1.05 (0.97-1.14)	0.237	1.18 (0.97-1.44)	0.094	1.08 (0.86-1.35)	0.522	0.97 (0.91-1.03)	0.301	0.97 (0.70-1.33)	0.828
**Age group at trial baseline**												
Age <65	1.00 (reference)		1.00 (reference)		1.00 (reference)		1.00 (reference)		1.00 (reference)		1.00 (reference)	
Age ≥65	2.02 (1.93-2.11)	<0.001	1.73 (1.60-1.87)	<0.001	1.71 (1.42-2.06)	<0.001	1.94 (1.56-2.41)	<0.001	2.25 (2.12-2.38)	<0.001	1.80 (1.33-2.43)	<0.001
**Gender**												
Male	1.00 (reference)		1.00 (reference)		1.00 (reference)		1.00 (reference)		1.00 (reference)		1.00 (reference)	
Female	0.78 (0.74-0.81)	<0.001	0.67 (0.62-0.72)	<0.001	0.69 (0.58-0.84)	<0.001	0.49 (0.39-0.62)	<0.001	0.88 (0.83-0.93)	<0.001	1.03 (0.77-1.38)	0.830
**Race/Ethnicity**												
Non-Black	1.00 (reference)		1.00 (reference)		1.00 (reference)		1.00 (reference)		1.00 (reference)		1.00 (reference)	
Black	0.94 (0.90-0.99)	0.013	1.08 (1.00-1.17)	0.040	1.32 (1.09-1.60)	0.004	0.73 (0.58-0.93)	0.010	0.85 (0.80-0.91)	<0.001	1.76 (1.32-2.34)	<0.001
**Hispanic/Latino Ethnicity**												
Non-Hispanic	1.00 (reference)		1.00 (reference)		1.00 (reference)		1.00 (reference)		1.00 (reference)		1.00 (reference)	
Hispanic	0.82 (0.78-0.87)	<0.001	0.62 (0.56-0.70)	<0.001	1.11 (0.88-1.40)	0.362	0.58 (0.43-0.80)	<0.001	0.93 (0.87-1.00)	0.050	1.03 (0.70-1.52)	0.863
**Education level at trial baseline**												
More than high school	1.00 (reference)		1.00 (reference)		1.00 (reference)		1.00 (reference)		1.00 (reference)		1.00 (reference)	
High school or less	1.20 (1.15-1.26)	<0.001	1.27 (1.18-1.38)	<0.001	1.43 (1.16-1.75)	<0.001	1.22 (0.98-1.52)	0.073	1.14 (1.07-1.21)	<0.001	0.91 (0.68-1.22)	0.535
**Treatment with antihypertensive drugs prior to trial baseline**												
Untreated	1.00 (reference)		1.00 (reference)		1.00 (reference)		1.00 (reference)		1.00 (reference)		1.00 (reference)	
Treated	1.10 (1.02-1.18)	0.011	1.02 (0.90-1.15)	0.740	1.30 (0.95-1.78)	0.106	0.92 (0.64-1.31)	0.632	1.14 (1.04-1.26)	0.007	1.27 (0.77-2.11)	0.346
**Aspirin use at trial baseline**												
No	1.00 (reference)		1.00 (reference)		1.00 (reference)		1.00 (reference)		1.00 (reference)		1.00 (reference)	
Yes	0.97 (0.92-1.01)	0.124	0.93 (0.86-1.00)	0.057	0.85 (0.70-1.04)	0.111	1.11 (0.89-1.37)	0.362	0.99 (0.93-1.05)	0.681	1.05 (0.79-1.41)	0.718
**GFR <60 mL/min/1.73 m^2 at trial baseline**												
No	1.00 (reference)		1.00 (reference)		1.00 (reference)		1.00 (reference)		1.00 (reference)		1.00 (reference)	
Yes	1.44 (1.37-1.51)	<0.001	1.22 (1.11-1.34)	<0.001	3.85 (3.20-4.62)	<0.001	1.15 (0.88-1.50)	0.313	1.43 (1.34-1.53)	<0.001	1.82 (1.35-2.47)	<0.001
**HDL cholesterol <35 mg/dl at trial baseline**												
No	1.00 (reference)		1.00 (reference)		1.00 (reference)		1.00 (reference)		1.00 (reference)		1.00 (reference)	
Yes	0.90 (0.85-0.96)	0.001	1.04 (0.94-1.15)	0.460	0.91 (0.69-1.20)	0.498	0.68 (0.49-0.95)	0.022	0.84 (0.78-0.92)	<0.001	1.21 (0.81-1.80)	0.352
**Cigarette smoking at trial baseline**												

© 2023 Yamal JM et al. *JAMA Network Open.*

	All Non-CVD Mortality		Cancer Mortality		Kidney Disease Mortality		Mortality Accident/Suicide		Other Non-CVD Mortality		Mortality Unknown Cause	
	Adjusted* HR (95% CI)	P value	Adjusted* HR (95% CI)	P value	Adjusted* HR (95% CI)	P value	Adjusted* HR (95% CI)	P value	Adjusted* HR (95% CI)	P value	Adjusted* HR (95% CI)	P value
Never smoker	1.00 (reference)		1.00 (reference)		1.00 (reference)		1.00 (reference)		1.00 (reference)		1.00 (reference)	
Current smoker	1.91 (1.81-2.02)	<0.001	2.59 (2.35-2.86)	<0.001	2.01 (1.58-2.56)	<0.001	1.36 (1.03-1.78)	0.030	1.62 (1.50-1.75)	<0.001	1.91 (1.32-2.76)	<0.001
Former Smoker	1.14 (1.09-1.20)	<0.001	1.33 (1.22-1.45)	<0.001	1.25 (1.02-1.53)	0.034	0.77 (0.61-0.97)	0.030	1.09 (1.03-1.16)	0.004	1.30 (0.94-1.80)	0.109
**Diabetes classification at trial baseline**												
Non-diabetes	1.00 (reference)		1.00 (reference)		1.00 (reference)		1.00 (reference)		1.00 (reference)		1.00 (reference)	
Diabetes	1.52 (1.45-1.58)	<0.001	1.10 (1.02-1.19)	0.014	2.43 (2.02-2.92)	<0.001	1.35 (1.09-1.68)	0.007	1.75 (1.65-1.85)	<0.001	1.74 (1.32-2.31)	<0.001
**History of Coronary Heart Disease (CHD) at trial baseline**												
No	1.00 (reference)		1.00 (reference)		1.00 (reference)		1.00 (reference)		1.00 (reference)		1.00 (reference)	
Yes	0.94 (0.88-1.00)	0.050	0.99 (0.89-1.10)	0.829	0.96 (0.73-1.26)	0.753	0.88 (0.65-1.19)	0.396	0.91 (0.84-0.99)	0.033	1.12 (0.76-1.66)	0.566
**Atherosclerotic Cardiovascular Disease (ASCVD) at trial baseline**												
No	1.00 (reference)		1.00 (reference)		1.00 (reference)		1.00 (reference)		1.00 (reference)		1.00 (reference)	
Yes	1.07 (1.00-1.16)	0.066	0.95 (0.83-1.08)	0.403	1.01 (0.73-1.39)	0.976	1.60 (1.09-2.34)	0.017	1.14 (1.03-1.26)	0.013	0.76 (0.47-1.23)	0.265
**History of myocardial infarction (MI) or stroke at trial baseline**												
No	1.00 (reference)		1.00 (reference)		1.00 (reference)		1.00 (reference)		1.00 (reference)		1.00 (reference)	
Yes	1.17 (1.10-1.25)	<0.001	1.15 (1.03-1.29)	0.013	1.28 (0.97-1.70)	0.085	0.80 (0.58-1.11)	0.186	1.20 (1.10-1.31)	<0.001	1.69 (1.12-2.54)	0.012
**History of coronary artery bypass graft (CABG) at trial baseline**												
No	1.00 (reference)		1.00 (reference)		1.00 (reference)		1.00 (reference)		1.00 (reference)		1.00 (reference)	
Yes	1.01 (0.94-1.09)	0.755	1.03 (0.91-1.16)	0.688	1.21 (0.88-1.65)	0.243	0.84 (0.59-1.20)	0.344	1.00 (0.91-1.10)	0.991	0.79 (0.50-1.27)	0.333
**Other ASCVD at trial baseline**												
No	1.00 (reference)		1.00 (reference)		1.00 (reference)		1.00 (reference)		1.00 (reference)		1.00 (reference)	
Yes	1.13 (1.06-1.21)	<0.001	1.27 (1.14-1.41)	<0.001	1.06 (0.80-1.40)	0.680	0.90 (0.65-1.24)	0.507	1.09 (1.00-1.19)	0.053	1.51 (1.03-2.23)	0.036
**Major ST segment depression at trial baseline**												
No	1.00 (reference)		1.00 (reference)		1.00 (reference)		1.00 (reference)		1.00 (reference)		1.00 (reference)	
Yes	0.97 (0.90-1.05)	0.445	1.06 (0.93-1.21)	0.412	1.29 (0.94-1.76)	0.114	0.76 (0.51-1.14)	0.182	0.91 (0.82-1.02)	0.095	1.67 (1.09-2.56)	0.020
**LVH by Minnesota code at trial baseline**												
No/Soft LVH	1.00 (reference)		1.00 (reference)		1.00 (reference)		1.00 (reference)		1.00 (reference)		1.00 (reference)	
Hard LVH	1.12 (1.02-1.23)	0.016	0.98 (0.83-1.16)	0.793	1.19 (0.84-1.70)	0.327	1.20 (0.75-1.92)	0.441	1.20 (1.06-1.35)	0.004	1.16 (0.69-1.95)	0.572
**Lipid Lowering Trial (LLT) Participant**												
No	1.00 (reference)		1.00 (reference)		1.00 (reference)		1.00 (reference)		1.00 (reference)		1.00 (reference)	
Yes	0.91 (0.87-0.95)	<0.001	0.95 (0.87-1.02)	0.166	0.93 (0.76-1.13)	0.461	0.97 (0.77-1.21)	0.771	0.88 (0.83-0.94)	<0.001	0.71 (0.51-1.00)	0.047
**Obesity (BMI ≥ 30 kg/m^2) at trial baseline**												
No	1.00 (reference)		1.00 (reference)		1.00 (reference)		1.00 (reference)		1.00 (reference)		1.00 (reference)	
Yes	0.94 (0.91-0.98)	0.006	0.93 (0.87-1.00)	0.049	1.08 (0.91-1.29)	0.358	0.88 (0.72-1.08)	0.225	0.95 (0.90-1.00)	0.043	0.83 (0.63-1.10)	0.188
**Baseline blood pressure reading, per 10 mmHg**												
Systolic BP	1.09 (1.07-1.11)	<0.001	1.04 (1.01-1.07)	0.008	1.26 (1.17-1.36)	<0.001	0.96 (0.88-1.05)	0.349	1.12 (1.09-1.15)	<0.001	1.09 (0.97-1.22)	0.156

© 2023 Yamal JM et al. *JAMA Network Open.*

	All Non-CVD Mortality		Cancer Mortality		Kidney Disease Mortality		Mortality Accident/Suicide		Other Non-CVD Mortality		Mortality Unknown Cause	
	**Adjusted* HR (95% CI)**	**P value**	**Adjusted* HR (95% CI)**	**P value**	**Adjusted* HR (95% CI)**	**P value**	**Adjusted* HR (95% CI)**	**P value**	**Adjusted* HR (95% CI)**	**P value**	**Adjusted* HR (95% CI)**	**P value**
Diastolic BP	0.86 (0.84-0.89)	<0.001	0.88 (0.84-0.92)	<0.001	0.89 (0.80-0.99)	0.029	0.84 (0.74-0.96)	0.008	0.86 (0.83-0.89)	<0.001	1.06 (0.89-1.25)	0.520

HR= Hazard ratio; 95% CI= 95% Confidence interval; CHD= coronary heart disease; CVD= cardiovascular disease; ASCVD= Atherosclerotic Cardiovascular Disease; OASCVD= Other ASCVD; HDL= high density lipoprotein; eGFR= estimated glomerular filtration rate; LVH= left ventricular hypertrophy; SBP= systolic blood pressure; DBP= diastolic blood pressure

[a] Adjusted for each covariate shown in that column. Covariates not included in multivariable analysis are indicated with dashes.

[b] First randomization date in the Mortality cohort.

Conversion factors: 1 mL/min/1.73m^2 = 0.0167 mL/s/m^2; 1 mg/dl = 0.0259 mmol/L; 1 mmHg = 0.133 kPa

© 2023 Yamal JM et al. *JAMA Network Open.*

eTable 5. Hazard Ratios Adjusted[a] by Variables Selected Through Penalized Regression for All-Cause and CVD Mortality With Follow-up From 2/23/1994[b] to 12/31/2017[c]

	All-Cause Mortality		CVD Mortality		CHD Mortality		Stroke Mortality		CHF Mortality		Other CVD Mortality	
	Adjusted HR (95% CI)	P value	Adjusted HR (95% CI)	P value	Adjusted HR (95% CI)	P value	Adjusted HR (95% CI)	P value	Adjusted HR (95% CI)	P value	Adjusted HR (95% CI)	P value
**Randomized group**												
Amlodipine vs. Chlorthalidone	1.00 (0.96-1.03)	0.827	0.96 (0.88-1.05)	0.388	1.01 (0.94-1.10)	0.763	1.00 (0.86-1.16)	0.968	1.00 (0.84-1.19)	0.990	1.01 (0.91-1.11)	0.914
Lisinopril vs Chlorthalidone	1.00 (0.97-1.04)	0.785	1.06 (0.98-1.16)	0.151	0.97 (0.90-1.05)	0.493	**1.19 (1.03-1.37)**	**0.017**	1.02 (0.86-1.21)	0.830	0.95 (0.86-1.05)	0.331
**Age group at trial baseline**												
Age <65	1.00 (reference)		1.00 (reference)		1.00 (reference)		1.00 (reference)		1.00 (reference)		1.00 (reference)	
Age ≥65	2.18 (2.11-2.25)	<0.001	2.35 (2.17-2.55)	<0.001	2.08 (1.93-2.24)	<0.001	2.54 (2.20-2.92)	<0.001	3.05 (2.56-3.63)	<0.001	2.47 (2.24-2.72)	<0.001
**Gender**												
Male	1.00 (reference)		1.00 (reference)		1.00 (reference)		1.00 (reference)		1.00 (reference)		1.00 (reference)	
Female	0.79 (0.76-0.82)	<0.001	0.85 (0.78-0.92)	<0.001	0.72 (0.67-0.78)	<0.001	0.90 (0.79-1.03)	0.143	0.90 (0.77-1.05)	0.176	0.80 (0.72-0.88)	<0.001
**Race/Ethnicity**												
Non-Black	1.00 (reference)		1.00 (reference)		1.00 (reference)		1.00 (reference)		-------	--------	1.00 (reference)	
Black	0.97 (0.94-1.00)	0.062	0.93 (0.86-1.01)	0.081	0.93 (0.86-1.01)	0.080	0.93 (0.81-1.08)	0.338	-------	--------	1.19 (1.08-1.31)	<0.001
**Hispanic/Latino Ethnicity**												
Non-Hispanic	1.00 (reference)		1.00 (reference)		1.00 (reference)		1.00 (reference)		1.00 (reference)		1.00 (reference)	
Hispanic	0.79 (0.76-0.83)	<0.001	0.85 (0.76-0.94)	0.002	0.79 (0.71-0.87)	<0.001	0.74 (0.62-0.89)	0.001	0.70 (0.56-0.88)	0.002	0.69 (0.60-0.79)	<0.001
**Education level at trial baseline**												
More than high school	1.00 (reference)		1.00 (reference)		1.00 (reference)		1.00 (reference)		1.00 (reference)		1.00 (reference)	
High school or less	1.19 (1.15-1.23)	<0.001	1.21 (1.11-1.31)	<0.001	1.24 (1.15-1.34)	<0.001	1.12 (0.97-1.29)	0.111	1.16 (0.98-1.36)	0.083	1.05 (0.96-1.16)	0.302
**Treatment with antihypertensive drugs prior to trial baseline**												
Untreated	-------	--------	1.00 (reference)		1.00 (reference)		1.00 (reference)		-------	--------	1.00 (reference)	
Treated	-------	--------	1.02 (0.90-1.16)	0.731	0.97 (0.86-1.09)	0.587	1.20 (0.95-1.51)	0.126	-------	--------	0.96 (0.82-1.11)	0.552
**Aspirin use at trial baseline**												
No	-------	--------	1.00 (reference)		-------	--------	1.00 (reference)		-------	--------	1.00 (reference)	
Yes	-------	--------	1.06 (0.98-1.14)	0.159	-------	--------	1.14 (0.99-1.30)	0.061	-------	--------	1.08 (0.98-1.18)	0.125
**GFR <60 mL/min/1.73 m^2 at trial baseline**												
No	1.00 (reference)		1.00 (reference)		1.00 (reference)		1.00 (reference)		1.00 (reference)		1.00 (reference)	
Yes	1.50 (1.44-1.55)	<0.001	1.59 (1.46-1.73)	<0.001	1.48 (1.37-1.61)	<0.001	1.60 (1.38-1.85)	<0.001	1.71 (1.44-2.03)	<0.001	1.62 (1.46-1.81)	<0.001
**HDL cholesterol <35 mg/dl at trial baseline**												
No	1.00 (reference)		1.00 (reference)		1.00 (reference)		1.00 (reference)		1.00 (reference)		1.00 (reference)	
Yes	0.89 (0.84-0.93)	<0.001	0.84 (0.75-0.94)	0.002	0.97 (0.88-1.07)	0.582	0.69 (0.55-0.85)	<0.001	0.79 (0.62-1.00)	0.049	0.82 (0.71-0.94)	0.006
	All-Cause Mortality		CVD Mortality		CHD Mortality		Stroke Mortality		CHF Mortality		Other CVD Mortality	

© 2023 Yamal JM et al. *JAMA Network Open.*

	Adjusted HR (95% CI)	P value	Adjusted HR (95% CI)	P value	Adjusted HR (95% CI)	P value	Adjusted HR (95% CI)	P value	Adjusted HR (95% CI)	P value	Adjusted HR (95% CI)	P value
**Cigarette smoking at trial baseline**												
Never smoker	1.00 (reference)		1.00 (reference)		1.00 (reference)		1.00 (reference)		-------	--------	1.00 (reference)	
Current smoker	1.68 (1.61-1.75)	<0.001	1.29 (1.17-1.44)	<0.001	1.46 (1.32-1.61)	<0.001	1.11 (0.92-1.33)	0.261	-------	--------	1.46 (1.29-1.65)	<0.001
Former Smoker	1.11 (1.07-1.15)	<0.001	1.05 (0.96-1.14)	0.299	1.12 (1.04-1.21)	0.004	0.95 (0.83-1.10)	0.517	-------	--------	1.07 (0.96-1.18)	0.219
**Diabetes classification at trial baseline**												
Non-diabetes	1.00 (reference)		1.00 (reference)		1.00 (reference)		1.00 (reference)		1.00 (reference)		1.00 (reference)	
Diabetes	1.54 (1.49-1.59)	<0.001	1.65 (1.53-1.78)	<0.001	1.70 (1.59-1.82)	<0.001	1.48 (1.29-1.68)	<0.001	1.64 (1.41-1.91)	<0.001	1.29 (1.18-1.42)	<0.001
**History of Coronary Heart Disease (CHD) at trial baseline**												
No	-------	--------	-------	--------	-------	--------	-------	--------	-------	--------	-------	--------
Yes	-------	--------	-------	--------	-------	--------	-------	--------	-------	--------	-------	--------
**Atherosclerotic Cardiovascular Disease (ASCVD) at trial baseline**												
No	-------	--------	-------	--------	-------	--------	-------	--------	-------	--------	-------	--------
Yes	-------	--------	-------	--------	-------	--------	-------	--------	-------	--------	-------	--------
**History of myocardial infarction (MI) or stroke at trial baseline**												
No	1.00 (reference)		1.00 (reference)		1.00 (reference)		1.00 (reference)		1.00 (reference)		1.00 (reference)	
Yes	1.31 (1.27-1.36)	<0.001	1.52 (1.41-1.65)	<0.001	1.63 (1.51-1.75)	<0.001	1.47 (1.28-1.70)	<0.001	1.26 (1.06-1.49)	0.008	1.33 (1.20-1.47)	<0.001
**History of coronary artery bypass graft (CABG) at trial baseline**												
No	1.00 (reference)		1.00 (reference)		1.00 (reference)		1.00 (reference)		1.00 (reference)		-------	--------
Yes	1.12 (1.07-1.17)	<0.001	1.21 (1.09-1.34)	<0.001	1.51 (1.39-1.65)	<0.001	0.91 (0.75-1.09)	0.303	1.39 (1.14-1.70)	<0.001	-------	--------
**Other ASCVD at trial baseline**												
No	1.00 (reference)		1.00 (reference)		1.00 (reference)		1.00 (reference)		1.00 (reference)		1.00 (reference)	
Yes	1.20 (1.16-1.24)	<0.001	1.24 (1.14-1.34)	<0.001	1.30 (1.20-1.40)	<0.001	1.30 (1.13-1.50)	<0.001	1.10 (0.93-1.32)	0.262	1.11 (1.00-1.24)	0.044
**Major ST segment depression at trial baseline**												
No	1.00 (reference)		1.00 (reference)		1.00 (reference)		1.00 (reference)		-------	--------	1.00 (reference)	
Yes	1.02 (0.97-1.08)	0.345	1.05 (0.94-1.18)	0.365	1.05 (0.94-1.17)	0.390	0.97 (0.79-1.19)	0.775	-------	--------	0.98 (0.85-1.13)	0.758
**LVH by Minnesota code at trial baseline**												
No/Soft LVH	1.00 (reference)		1.00 (reference)		1.00 (reference)		1.00 (reference)		1.00 (reference)		1.00 (reference)	
Hard LVH	1.40 (1.31-1.49)	<0.001	1.75 (1.53-2.01)	<0.001	1.63 (1.42-1.86)	<0.001	1.81 (1.44-2.28)	<0.001	1.96 (1.50-2.55)	<0.001	1.72 (1.46-2.03)	<0.001
**Lipid Lowering Trial (LLT) Participant**												
No	1.00 (reference)		1.00 (reference)		1.00 (reference)		1.00 (reference)		1.00 (reference)		-------	--------
Yes	0.94 (0.91-0.97)	<0.001	0.96 (0.88-1.04)	0.301	0.97 (0.90-1.05)	0.449	-------	--------	0.86 (0.72-1.02)	0.091	-------	--------
**Obesity (BMI ≥ 30 kg/m²) at trial baseline**												
No	1.00 (reference)		1.00 (reference)		1.00 (reference)		1.00 (reference)		1.00 (reference)		1.00 (reference)	
Yes	0.95 (0.92-0.98)	0.001	0.98 (0.91-1.05)	0.535	0.98 (0.92-1.05)	0.630	0.85 (0.75-0.97)	0.015	1.14 (0.98-1.32)	0.084	0.97 (0.88-1.06)	0.445
**Baseline blood pressure reading, per 10 mmHg**												
	**All-Cause Mortality**		**CVD Mortality**		**CHD Mortality**		**Stroke Mortality**		**CHF Mortality**		**Other CVD Mortality**	

© 2023 Yamal JM et al. *JAMA Network Open.*

	Adjusted HR (95% CI)	P value	Adjusted HR (95% CI)	P value	Adjusted HR (95% CI)	P value	Adjusted HR (95% CI)	P value	Adjusted HR (95% CI)	P value	Adjusted HR (95% CI)	P value
Systolic BP	-------	--------	-------	--------	-------	--------	-------	--------	-------	--------	-------	--------
Diastolic BP	0.94 (0.92-0.96)	<0.001	0.96 (0.92-1.00)	0.064	0.95 (0.92-0.99)	0.013	1.07 (0.99-1.15)	0.072	0.83 (0.77-0.90)	<0.001	1.02 (0.97-1.07)	0.514

HR= Hazard ratio; 95% CI= 95% Confidence interval; CHD= coronary heart disease; CVD= cardiovascular disease; ASCVD= Atherosclerotic Cardiovascular Disease; OASCVD= Other ASCVD; HDL= high density lipoprotein; eGFR= estimated glomerular filtration rate; LVH= left ventricular hypertrophy; SBP= systolic blood pressure; DBP= diastolic blood pressure

[a] Adjusted for each covariate shown in that column using LASSO penalty for variable selection (no penalty for the randomization group). Covariates not included in multivariable analysis are indicated with dashes.

[b] First randomization date in the Mortality cohort.

[c] Correlation between candidate variables was assessed prior to initiation of Least Absolute Shrinkage and Selection Operator (LASSO) regression procedures; variables that demonstrated moderate to high correlation with each other/other candidate variables were subject to preliminary removal. These included ASCVD, history of CHD, and baseline systolic blood pressure reading.

Conversion factors: 1 mL/min/1.73m^2 = 0.0167 mL/s/m^2; 1 mg/dl = 0.0259 mmol/L; 1 mmHg = 0.133 kPa

© 2023 Yamal JM et al. *JAMA Network Open.*

eTable 6. Hazard Ratios Adjusted [a] by Variables Selected Through Penalized Regression for Morbidity With Follow-up From 3/10/1994 [b] to 12/31/2017[c]

	CVD Morbidity		CHD Morbidity		CHF Morbidity		Stroke Morbidity		Cancer Morbidity		Kidney Disease Morbidity	
	**Adjusted* HR (95% CI)**	**P value**	**Adjusted* HR (95% CI)**	**P value**	**Adjusted* HR (95% CI)**	**P value**	**Adjusted* HR (95% CI)**	**P value**	**Adjusted* HR (95% CI)**	**P value**	**Adjusted* HR (95% CI)**	**P value**
**Randomized group**												
Amlodipine vs. Chlorthalidone	1.01 (0.96-1.06)	0.701	0.99 (0.92-1.06)	0.780	1.02 (0.96-1.08)	0.495	1.03 (0.95-1.12)	0.428	0.99 (0.92-1.07)	0.835	1.05 (0.90-1.22)	0.536
Lisinopril vs Chlorthalidone	1.04 (0.99-1.09)	0.150	0.98 (0.91-1.05)	0.520	1.03 (0.97-1.09)	0.364	**1.11 (1.03-1.20)**	**0.007**	1.02 (0.94-1.10)	0.626	1.13 (0.98-1.31)	0.091
**Age group at trial baseline**												
Age <65	1.00 (reference)		1.00 (reference)		1.00 (reference)		1.00 (reference)		1.00 (reference)		1.00 (reference)	
Age ≥65	1.82 (1.74-1.92)	<0.001	1.74 (1.62-1.86)	<0.001	1.92 (1.81-2.03)	<0.001	1.86 (1.72-2.01)	<0.001	1.64 (1.52-1.76)	<0.001	1.10 (0.96-1.26)	0.169
**Gender**												
Male	1.00 (reference)		1.00 (reference)		1.00 (reference)		1.00 (reference)		1.00 (reference)		1.00 (reference)	
Female	0.93 (0.89-0.98)	0.004	0.82 (0.77-0.88)	<0.001	0.98 (0.93-1.04)	0.483	0.97 (0.91-1.05)	0.499	0.69 (0.64-0.74)	<0.001	0.67 (0.58-0.77)	<0.001
**Race/Ethnicity**												
Non-Black	1.00 (reference)		1.00 (reference)		1.00 (reference)		1.00 (reference)		1.00 (reference)		1.00 (reference)	
Black	0.96 (0.91-1.01)	0.110	0.89 (0.83-0.96)	0.002	0.97 (0.92-1.03)	0.328	1.01 (0.94-1.09)	0.766	1.06 (0.98-1.14)	0.157	1.86 (1.63-2.13)	<0.001
**Hispanic/Latino Ethnicity**												
Non-Hispanic	1.00 (reference)		1.00 (reference)		1.00 (reference)		1.00 (reference)		1.00 (reference)		-------	--------
Hispanic	0.71 (0.67-0.76)	<0.001	0.79 (0.72-0.86)	<0.001	0.62 (0.58-0.67)	<0.001	0.74 (0.67-0.82)	<0.001	0.62 (0.56-0.69)	<0.001	-------	--------
**Education level at trial baseline**												
More than high school	1.00 (reference)		1.00 (reference)		1.00 (reference)		1.00 (reference)		1.00 (reference)		1.00 (reference)	
High school or less	1.20 (1.14-1.26)	<0.001	1.21 (1.13-1.30)	<0.001	1.20 (1.13-1.27)	<0.001	1.16 (1.07-1.25)	<0.001	1.13 (1.05-1.22)	0.002	1.09 (0.94-1.26)	0.254
**Treatment with antihypertensive drugs prior to trial baseline**												
Untreated	1.00 (reference)		1.00 (reference)		1.00 (reference)		1.00 (reference)		1.00 (reference)		-------	--------
Treated	1.15 (1.07-1.25)	<0.001	0.98 (0.88-1.09)	0.727	1.11 (1.01-1.22)	0.029	1.18 (1.05-1.34)	0.007	1.07 (0.95-1.21)	0.246	-------	--------
**Aspirin use at trial baseline**												
No	1.00 (reference)		1.00 (reference)		1.00 (reference)		1.00 (reference)		1.00 (reference)		1.00 (reference)	
Yes	1.06 (1.02-1.12)	0.009	1.06 (0.99-1.13)	0.093	1.03 (0.97-1.09)	0.312	1.15 (1.07-1.23)	<0.001	0.92 (0.85-0.98)	0.017	0.90 (0.78-1.04)	0.165
**GFR <60 mL/min/1.73 m² at trial baseline**												
No	1.00 (reference)		1.00 (reference)		1.00 (reference)		1.00 (reference)		1.00 (reference)		1.00 (reference)	
Yes	1.41 (1.34-1.49)	<0.001	1.42 (1.32-1.53)	<0.001	1.55 (1.46-1.64)	<0.001	1.31 (1.21-1.42)	<0.001	1.19 (1.09-1.29)	<0.001	5.25 (4.62-5.97)	<0.001
**HDL cholesterol <35 mg/dl at trial baseline**												
No	1.00 (reference)		1.00 (reference)		1.00 (reference)		1.00 (reference)		1.00 (reference)		1.00 (reference)	
Yes	0.94 (0.87-1.00)	0.066	1.06 (0.97-1.16)	0.217	0.92 (0.85-1.00)	0.045	0.86 (0.77-0.97)	0.010	1.03 (0.93-1.14)	0.599	0.96 (0.78-1.19)	0.704
**Cigarette smoking at trial baseline**												

© 2023 Yamal JM et al. *JAMA Network Open.*

	CVD Morbidity		CHD Morbidity		CHF Morbidity		Stroke Morbidity		Cancer Morbidity		Kidney Disease Morbidity	
	Adjusted* HR (95% CI)	P value	Adjusted* HR (95% CI)	P value	Adjusted* HR (95% CI)	P value	Adjusted* HR (95% CI)	P value	Adjusted* HR (95% CI)	P value	Adjusted* HR (95% CI)	P value
Never smoker	1.00 (reference)		1.00 (reference)		1.00 (reference)		1.00 (reference)		1.00 (reference)		1.00 (reference)	
Current smoker	1.40 (1.31-1.49)	<0.001	1.47 (1.34-1.61)	<0.001	1.38 (1.28-1.49)	<0.001	1.41 (1.28-1.55)	<0.001	2.03 (1.86-2.22)	<0.001	1.76 (1.47-2.11)	<0.001
Former Smoker	1.09 (1.04-1.15)	<0.001	1.13 (1.05-1.21)	<0.001	1.08 (1.02-1.15)	0.007	1.11 (1.03-1.20)	0.009	1.24 (1.14-1.34)	<0.001	1.23 (1.06-1.42)	0.005
**Diabetes classification at trial baseline**												
Non-diabetes	1.00 (reference)		1.00 (reference)		1.00 (reference)		1.00 (reference)		-------	--------	1.00 (reference)	
Diabetes	1.58 (1.51-1.65)	<0.001	1.70 (1.60-1.82)	<0.001	1.70 (1.61-1.79)	<0.001	1.51 (1.41-1.62)	<0.001	-------	--------	3.17 (2.77-3.63)	<0.001
**History of Coronary Heart Disease (CHD) at trial baseline**												
No	-------	--------	-------	--------	-------	--------	-------	--------	-------	--------	-------	--------
Yes	-------	--------	-------	--------	-------	--------	-------	--------	-------	--------	-------	--------
**Atherosclerotic Cardiovascular Disease (ASCVD) at trial baseline**												
No	-------	--------	-------	--------	-------	--------	-------	--------	-------	--------	-------	--------
Yes	-------	--------	-------	--------	-------	--------	-------	--------	-------	--------	-------	--------
**History of myocardial infarction (MI) or stroke at trial baseline**												
No	1.00 (reference)		1.00 (reference)		1.00 (reference)		1.00 (reference)		-------	--------	1.00 (reference)	
Yes	1.46 (1.39-1.53)	<0.001	1.52 (1.42-1.63)	<0.001	1.45 (1.37-1.54)	<0.001	1.38 (1.28-1.50)	<0.001	-------	--------	1.17 (1.00-1.36)	0.051
**History of coronary artery bypass graft (CABG) at trial baseline**												
No	1.00 (reference)		1.00 (reference)		1.00 (reference)		1.00 (reference)		-------	--------	1.00 (reference)	
Yes	1.29 (1.22-1.38)	<0.001	1.53 (1.41-1.66)	<0.001	1.32 (1.23-1.42)	<0.001	1.26 (1.15-1.39)	<0.001	-------	--------	1.27 (1.04-1.55)	0.017
**Other ASCVD at trial baseline**												
No	1.00 (reference)		1.00 (reference)		1.00 (reference)		1.00 (reference)		1.00 (reference)		1.00 (reference)	
Yes	1.19 (1.14-1.26)	<0.001	1.23 (1.15-1.32)	<0.001	1.19 (1.12-1.26)	<0.001	1.20 (1.11-1.30)	<0.001	1.13 (1.05-1.22)	0.001	1.08 (0.92-1.27)	0.324
**Major ST segment depression at trial baseline**												
No	1.00 (reference)		1.00 (reference)		1.00 (reference)		1.00 (reference)		-------	--------	1.00 (reference)	
Yes	1.10 (1.03-1.18)	0.006	1.01 (0.91-1.11)	0.873	1.18 (1.09-1.27)	<0.001	1.00 (0.89-1.11)	0.938	-------	--------	1.20 (0.98-1.46)	0.078
**LVH by Minnesota code at trial baseline**												
No/Soft LVH	1.00 (reference)		1.00 (reference)		1.00 (reference)		1.00 (reference)		-------	--------	1.00 (reference)	
Hard LVH	1.61 (1.48-1.76)	<0.001	1.53 (1.35-1.73)	<0.001	1.81 (1.64-2.00)	<0.001	1.57 (1.37-1.79)	<0.001	-------	--------	1.49 (1.17-1.89)	0.001
**Lipid Lowering Trial (LLT) Participant**												
No	1.00 (reference)		1.00 (reference)		1.00 (reference)		1.00 (reference)		1.00 (reference)		-------	--------
Yes	0.95 (0.90-0.99)	0.025	0.93 (0.87-1.00)	0.050	0.94 (0.88-0.99)	0.024	0.96 (0.89-1.04)	0.293	0.93 (0.86-1.00)	0.044	-------	--------
**Obesity (BMI ≥ 30 kg/m²) at trial baseline**												
No	1.00 (reference)		1.00 (reference)		1.00 (reference)		1.00 (reference)		1.00 (reference)		1.00 (reference)	
Yes	1.08 (1.04-1.13)	<0.001	0.93 (0.87-0.99)	0.028	1.24 (1.18-1.30)	<0.001	0.93 (0.87-0.99)	0.034	1.02 (0.95-1.09)	0.609	1.15 (1.02-1.31)	0.027
**Baseline blood pressure reading, per 10 mmHg**												
Systolic BP	-------	--------	-------	--------	-------	--------	-------	--------	-------	--------	-------	--------

© 2023 Yamal JM et al. *JAMA Network Open.*

	CVD Morbidity		CHD Morbidity		CHF Morbidity		Stroke Morbidity		Cancer Morbidity		Kidney Disease Morbidity	
	**Adjusted* HR (95% CI)**	**P value**	**Adjusted* HR (95% CI)**	**P value**	**Adjusted* HR (95% CI)**	**P value**	**Adjusted* HR (95% CI)**	**P value**	**Adjusted* HR (95% CI)**	**P value**	**Adjusted* HR (95% CI)**	**P value**
Diastolic BP	0.95 (0.93-0.98)	<0.001	0.98 (0.95-1.02)	0.411	0.92 (0.90-0.95)	<0.001	1.00 (0.96-1.04)	0.834	0.92 (0.89-0.96)	<0.001	1.06 (0.98-1.14)	0.136

HR= Hazard ratio; 95% CI= 95% Confidence interval; CHD= coronary heart disease; CVD= cardiovascular disease; ASCVD= Atherosclerotic Cardiovascular Disease; OASCVD= Other ASCVD; HDL= high density lipoprotein; eGFR= estimated glomerular filtration rate; LVH= left ventricular hypertrophy; SBP= systolic blood pressure; DBP= diastolic blood pressure

[a] Adjusted for each covariate shown in that column using LASSO penalty for variable selection (no penalty for the randomization group). Covariates not included in multivariable analysis are indicated with dashes.

[b] First randomization date in the full Morbidity cohort

[c] Correlation between candidate variables was assessed prior to initiation of Least Absolute Shrinkage and Selection Operator (LASSO) regression procedures; variables that demonstrated moderate to high correlation with each other/other candidate variables were subject to preliminary removal. These included ASCVD, history of CHD, and baseline systolic blood pressure

Conversion factors: 1 mL/min/1.73m^2 = 0.0167 mL/s/m^2; 1 mg/dl = 0.0259 mmol/L; 1 mmHg = 0.133 kPa

© 2023 Yamal JM et al. *JAMA Network Open.*

eTable 7. Hazard Ratios Adjusted[a] by Variables Selected Through Penalized Regression for Non-CVD Mortality With Follow-up From 2/23/1994[b] to 12/31/2017†

	All Non-CVD Mortality		Cancer Mortality		Kidney Disease Mortality		Mortality Accident/Suicide		Other Non-CVD Mortality		Mortality Unknown Cause	
	**Adjusted HR (95% CI)**	**P value**	**Adjusted HR (95% CI)**	**P value**	**Adjusted HR (95% CI)**	**P value**	**Adjusted HR (95% CI)**	**P value**	**Adjusted HR (95% CI)**	**P value**	**Adjusted HR (95% CI)**	**P value**
**Randomized group**												
Amlodipine vs. Chlorthalidone	0.99 (0.94-1.04)	0.617	0.97 (0.90-1.05)	0.490	1.00 (0.82-1.24)	0.966	0.85 (0.67-1.08)	0.187	1.01 (0.95-1.08)	0.650	0.97 (0.72-1.31)	0.845
Lisinopril vs Chlorthalidone	1.01 (0.96-1.06)	0.681	1.06 (0.98-1.14)	0.154	1.18 (0.97-1.44)	0.094	1.08 (0.86-1.35)	0.518	0.97 (0.91-1.03)	0.292	0.95 (0.70-1.29)	0.762
**Age group at trial baseline**												
Age <65	1.00 (reference)		1.00 (reference)		1.00 (reference)		1.00 (reference)		1.00 (reference)		1.00 (reference)	
Age ≥65	2.09 (2.00-2.18)	<0.001	1.73 (1.61-1.85)	<0.001	1.84 (1.53-2.21)	<0.001	1.92 (1.55-2.37)	<0.001	2.35 (2.22-2.49)	<0.001	1.87 (1.40-2.49)	<0.001
**Gender**												
Male	1.00 (reference)		1.00 (reference)		1.00 (reference)		1.00 (reference)		1.00 (reference)		-------	--------
Female	0.79 (0.76-0.82)	<0.001	0.68 (0.63-0.73)	<0.001	0.73 (0.61-0.88)	<0.001	0.49 (0.39-0.61)	<0.001	0.90 (0.85-0.95)	<0.001	-------	--------
**Race/Ethnicity**												
Non-Black	1.00 (reference)		1.00 (reference)		1.00 (reference)		1.00 (reference)		1.00 (reference)		1.00 (reference)	
Black	0.93 (0.89-0.97)	0.002	1.07 (1.00-1.15)	0.051	1.26 (1.05-1.51)	0.013	0.75 (0.60-0.95)	0.016	0.84 (0.79-0.89)	<0.001	1.65 (1.27-2.15)	<0.001
**Hispanic/Latino Ethnicity**												
Non-Hispanic	1.00 (reference)		1.00 (reference)		-------	--------	1.00 (reference)		1.00 (reference)		-------	--------
Hispanic	0.82 (0.77-0.86)	<0.001	0.64 (0.58-0.70)	<0.001	-------	--------	0.61 (0.45-0.83)	0.002	0.93 (0.87-1.00)	0.046	-------	--------
**Education level at trial baseline**												
More than high school	1.00 (reference)		1.00 (reference)		1.00 (reference)		1.00 (reference)		1.00 (reference)		-------	--------
High school or less	1.21 (1.16-1.27)	<0.001	1.26 (1.17-1.36)	<0.001	1.48 (1.21-1.81)	<0.001	1.22 (0.98-1.51)	0.074	1.15 (1.09-1.22)	<0.001	-------	--------
**Treatment with antihypertensive drugs prior to trial baseline**												
Untreated	-------	--------	1.00 (reference)		-------	--------	-------	--------	-------	--------	-------	--------
Treated	-------	--------	0.97 (0.87-1.09)	0.651	-------	--------	-------	--------	-------	--------	-------	--------
**Aspirin use at trial baseline**												
No	1.00 (reference)		1.00 (reference)		1.00 (reference)		1.00 (reference)		-------	--------	-------	--------
Yes	0.97 (0.93-1.01)	0.181	0.90 (0.84-0.97)	0.006	0.86 (0.71-1.04)	0.113	1.12 (0.92-1.38)	0.255	-------	--------	-------	--------
**GFR <60 mL/min/1.73 m^2 at trial baseline**												
No	1.00 (reference)		1.00 (reference)		1.00 (reference)		1.00 (reference)		1.00 (reference)		1.00 (reference)	
Yes	1.44 (1.36-1.51)	<0.001	1.21 (1.10-1.32)	<0.001	3.83 (3.19-4.59)	<0.001	1.13 (0.87-1.48)	0.352	1.43 (1.34-1.53)	<0.001	1.86 (1.40-2.48)	<0.001
**HDL cholesterol <35 mg/dl at trial baseline**												
No	1.00 (reference)		1.00 (reference)		1.00 (reference)		1.00 (reference)		1.00 (reference)		-------	--------
Yes	0.89 (0.83-0.94)	<0.001	1.05 (0.95-1.16)	0.343	0.87 (0.66-1.15)	0.329	0.73 (0.54-0.99)	0.046	0.82 (0.76-0.89)	<0.001	-------	--------
	**All Non-CVD Mortality**		**Cancer Mortality**		**Kidney Disease Mortality**		**Mortality Accident/Suicide**		**Other Non-CVD Mortality**		**Mortality Unknown Cause**	

© 2023 Yamal JM et al. *JAMA Network Open.*

	Adjusted HR (95% CI)	P value	Adjusted HR (95% CI)	P value	Adjusted HR (95% CI)	P value	Adjusted HR (95% CI)	P value	Adjusted HR (95% CI)	P value	Adjusted HR (95% CI)	P value
**Cigarette smoking at trial baseline**												
Never smoker	1.00 (reference)		1.00 (reference)		1.00 (reference)		1.00 (reference)		1.00 (reference)		1.00 (reference)	
Current smoker	1.90 (1.80-2.01)	<0.001	2.58 (2.36-2.82)	<0.001	1.99 (1.57-2.53)	<0.001	1.27 (0.97-1.66)	0.082	1.59 (1.48-1.72)	<0.001	1.87 (1.33-2.63)	<0.001
Former Smoker	1.15 (1.09-1.20)	<0.001	1.32 (1.22-1.44)	<0.001	1.25 (1.02-1.53)	0.033	0.75 (0.60-0.95)	0.015	1.09 (1.03-1.16)	0.004	1.26 (0.94-1.68)	0.126
**Diabetes classification at trial baseline**												
Non-diabetes	1.00 (reference)		1.00 (reference)		1.00 (reference)		1.00 (reference)		1.00 (reference)		1.00 (reference)	
Diabetes	1.53 (1.47-1.60)	<0.001	1.12 (1.05-1.20)	0.001	2.52 (2.12-3.00)	<0.001	1.25 (1.01-1.53)	0.037	1.77 (1.67-1.87)	<0.001	1.77 (1.36-2.30)	<0.001
**History of Coronary Heart Disease (CHD) at trial baseline**												
No	-------	--------	-------	--------	-------	--------	-------	--------	-------	--------	-------	--------
Yes	-------	--------	-------	--------	-------	--------	-------	--------	-------	--------	-------	--------
**Atherosclerotic Cardiovascular Disease (ASCVD) at trial baseline**												
No	-------	--------	-------	--------	-------	--------	-------	--------	-------	--------	-------	--------
Yes	-------	--------	-------	--------	-------	--------	-------	--------	-------	--------	-------	--------
**History of myocardial infarction (MI) or stroke at trial baseline**												
No	1.00 (reference)		1.00 (reference)		1.00 (reference)		-------	--------	1.00 (reference)		1.00 (reference)	
Yes	1.18 (1.13-1.24)	<0.001	1.15 (1.06-1.24)	<0.001	1.27 (1.03-1.57)	0.024	-------	--------	1.24 (1.16-1.32)	<0.001	1.54 (1.16-2.04)	0.002
**History of coronary artery bypass graft (CABG) at trial baseline**												
No	1.00 (reference)		1.00 (reference)		1.00 (reference)		-------	--------	1.00 (reference)		1.00 (reference)	
Yes	1.00 (0.94-1.07)	0.922	1.00 (0.90-1.11)	0.989	1.18 (0.90-1.55)	0.224	-------	--------	1.00 (0.92-1.08)	0.967	0.80 (0.54-1.20)	0.284
**Other ASCVD at trial baseline**												
No	1.00 (reference)		1.00 (reference)		-------	--------	1.00 (reference)		1.00 (reference)		1.00 (reference)	
Yes	1.17 (1.12-1.23)	<0.001	1.20 (1.11-1.30)	<0.001	-------	--------	1.12 (0.89-1.42)	0.342	1.16 (1.09-1.23)	<0.001	1.31 (0.98-1.76)	0.072
**Major ST segment depression at trial baseline**												
No	1.00 (reference)		1.00 (reference)		1.00 (reference)		-------	--------	1.00 (reference)		1.00 (reference)	
Yes	1.01 (0.95-1.08)	0.680	1.02 (0.92-1.14)	0.673	1.32 (1.01-1.71)	0.039	-------	--------	0.99 (0.90-1.08)	0.763	1.46 (1.01-2.10)	0.042
**LVH by Minnesota code at trial baseline**												
No/Soft LVH	1.00 (reference)		-------	--------	1.00 (reference)		1.00 (reference)		1.00 (reference)		1.00 (reference)	
Hard LVH	1.16 (1.06-1.28)	0.001	-------	--------	1.29 (0.90-1.83)	0.162	1.12 (0.71-1.79)	0.622	1.25 (1.11-1.42)	<0.001	1.16 (0.70-1.92)	0.555
**Lipid Lowering Trial (LLT) Participant**												
No	1.00 (reference)		1.00 (reference)		1.00 (reference)		-------	--------	1.00 (reference)		1.00 (reference)	
Yes	0.91 (0.87-0.95)	<0.001	0.95 (0.88-1.02)	0.162	0.93 (0.77-1.13)	0.478	-------	--------	0.89 (0.84-0.94)	<0.001	0.68 (0.49-0.94)	0.018
**Obesity (BMI ≥ 30 kg/m²) at trial baseline**												
No	1.00 (reference)		1.00 (reference)		-------	--------	1.00 (reference)		1.00 (reference)		1.00 (reference)	
Yes	0.94 (0.90-0.98)	0.002	0.91 (0.85-0.97)	0.005	-------	--------	0.89 (0.73-1.09)	0.262	0.94 (0.89-0.99)	0.023	0.83 (0.64-1.08)	0.158
**Baseline blood pressure reading, per 10 mmHg**												
	**All Non-CVD Mortality**		**Cancer Mortality**		**Kidney Disease Mortality**		**Mortality Accident/Suicide**		**Other Non-CVD Mortality**		**Mortality Unknown Cause**	

© 2023 Yamal JM et al. *JAMA Network Open.*

	Adjusted HR (95% CI)	P value	Adjusted HR (95% CI)	P value	Adjusted HR (95% CI)	P value	Adjusted HR (95% CI)	P value	Adjusted HR (95% CI)	P value	Adjusted HR (95% CI)	P value
Systolic BP	-------	--------	-------	--------	-------	--------	-------	--------	-------	--------	-------	--------
Diastolic BP	0.91 (0.89-0.93)	<0.001	0.90 (0.87-0.94)	<0.001	-------	--------	0.82 (0.74-0.92)	<0.001	0.92 (0.89-0.94)	<0.001	1.04 (0.91-1.20)	0.557

HR= Hazard ratio; 95% CI= 95% Confidence interval; CHD= coronary heart disease; CVD= cardiovascular disease; ASCVD= Atherosclerotic Cardiovascular Disease; OASCVD= Other ASCVD; HDL= high density lipoprotein; eGFR= estimated glomerular filtration rate; LVH= left ventricular hypertrophy; SBP= systolic blood pressure; DBP= diastolic blood pressure

†Correlation between candidate variables was assessed prior to initiation of Least Absolute Shrinkage and Selection Operator (LASSO) regression procedures; variables that demonstrated moderate to high correlation with each other/other candidate variables were subject to preliminary removal. These included ASCVD, history of CHD, and baseline systolic blood pressure reading.

[a] Adjusted for each covariate shown in that column using LASSO penalty for variable selection (no penalty for the randomization group). Covariates not included in multivariable analysis are indicated with dashes.

[b] First randomization date in the Mortality cohort

Conversion factors: 1 mL/min/1.73m^2 = 0.0167 mL/s/m^2; 1 mg/dl = 0.0259 mmol/L; 1 mmHg = 0.133 kPa

© 2023 Yamal JM et al. *JAMA Network Open.*

eFigure 1A. Kaplan-Meier Plots of Primary and Secondary Outcomes Through the Extended Follow-up (by 12/31/2017)

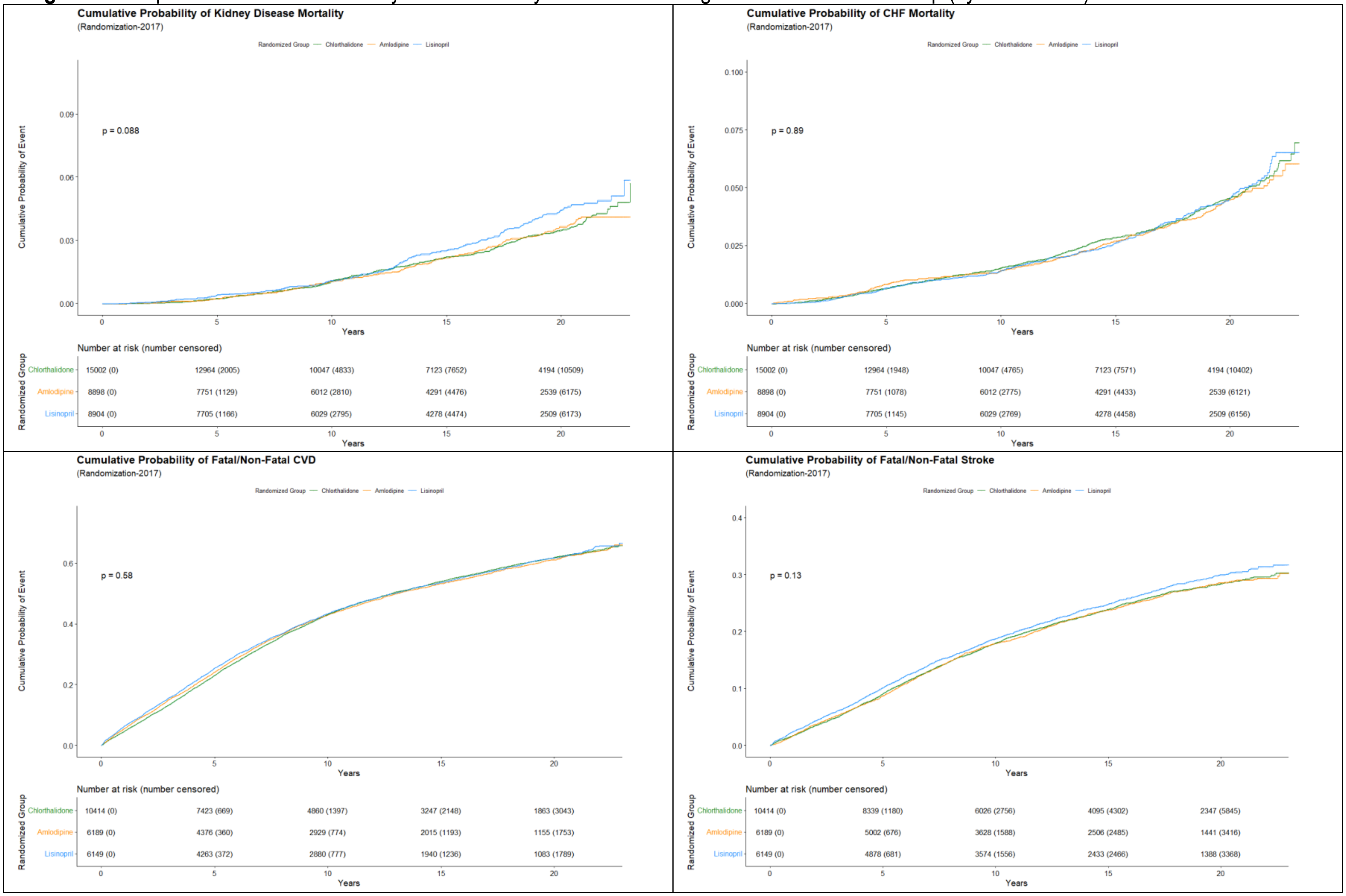

© 2023 Yamal JM et al. *JAMA Network Open.*

eFigure 1B. Kaplan-Meier Plots of Primary and Secondary Outcomes Through the Extended Follow-up (by 12/31/2017)

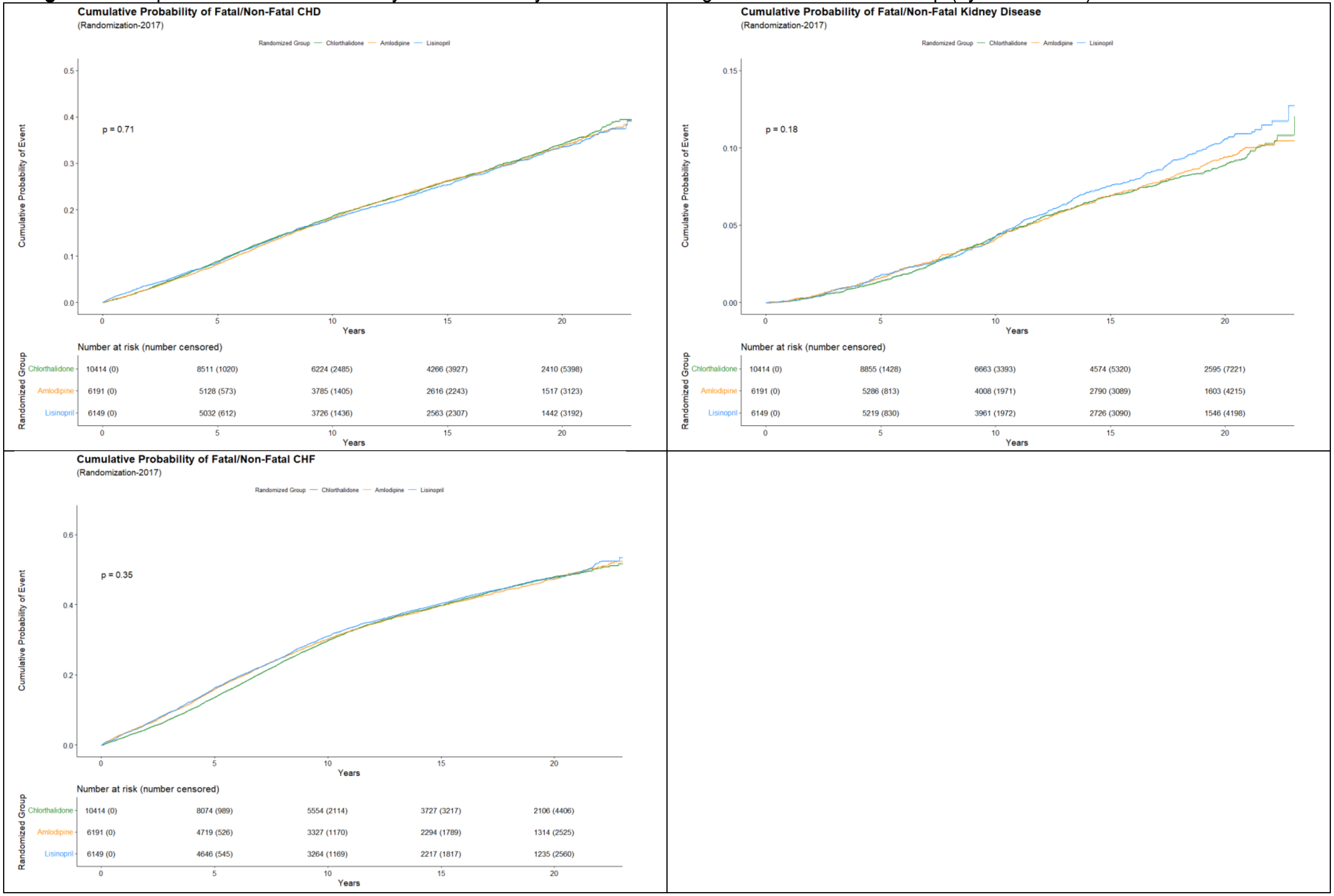

© 2023 Yamal JM et al. *JAMA Network Open.*

eFigure 2A. Unadjusted Hazard Ratios (95% CI) and Forest Plots of Overall and Subgroup Analyses of Mortality and Morbidity Outcomes Through the Extended Follow-up (by 12/31/2017) – Amlodipine vs. Chlorthalidone and Lisinopril vs. Chlorthalidone[a]

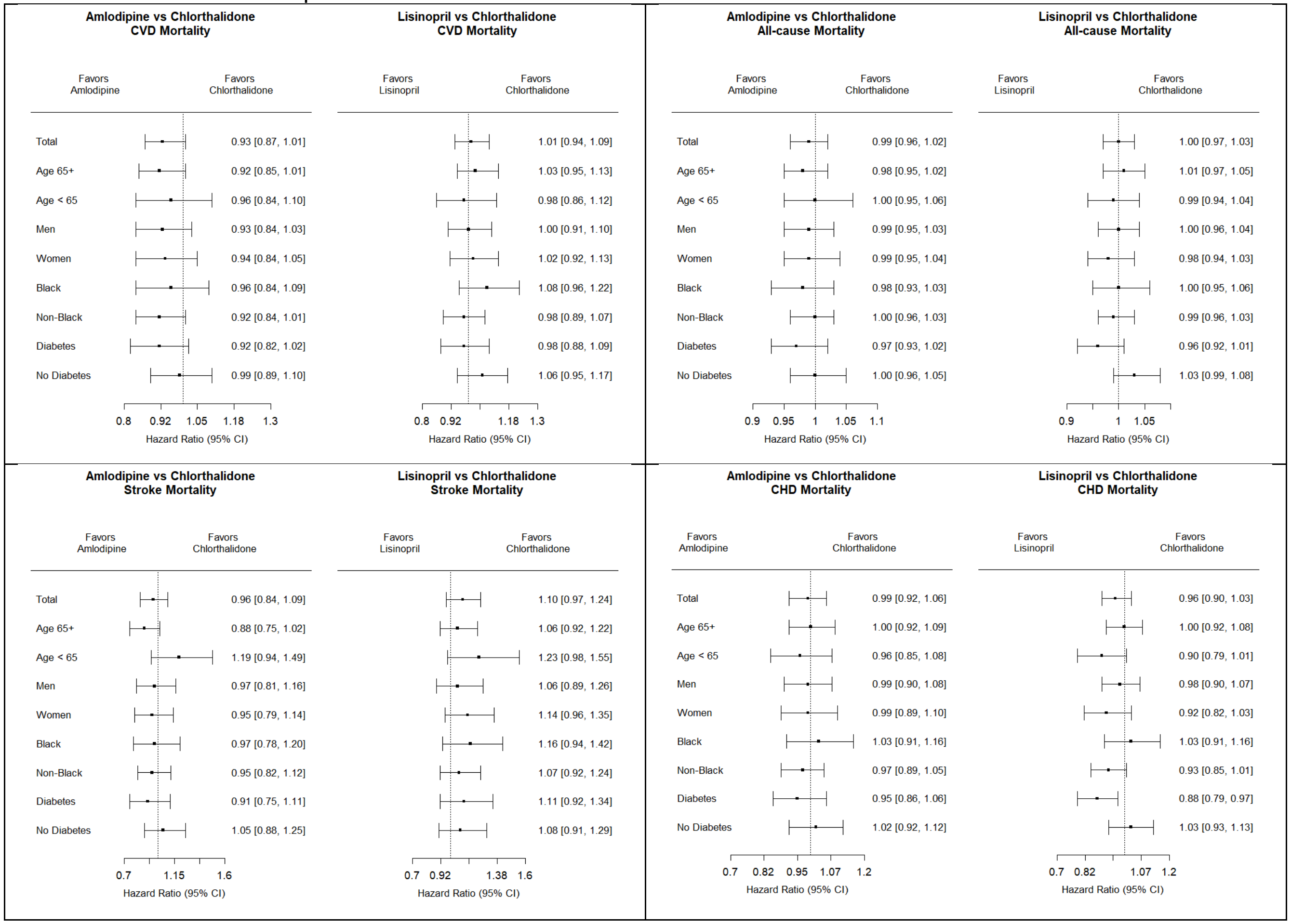

[a] Significant interactions were detected between lisinopril/chlorthalidone × diabetes status for all-cause mortality (p=0.024) and for CHD mortality (p=0.027). Significant interaction was detected for amlodipine/chlorthalidone × age 65+ for stroke mortality (p=0.033).

© 2023 Yamal JM et al. *JAMA Network Open.*

eFigure 2B. Unadjusted Hazard Ratios (95% CI) and Forest Plots of Overall and Subgroup Analyses of Mortality and Morbidity Outcomes Through the Extended Follow-up (by 12/31/2017) – Amlodipine vs. Chlorthalidone and Lisinopril vs. Chlorthalidone[a]

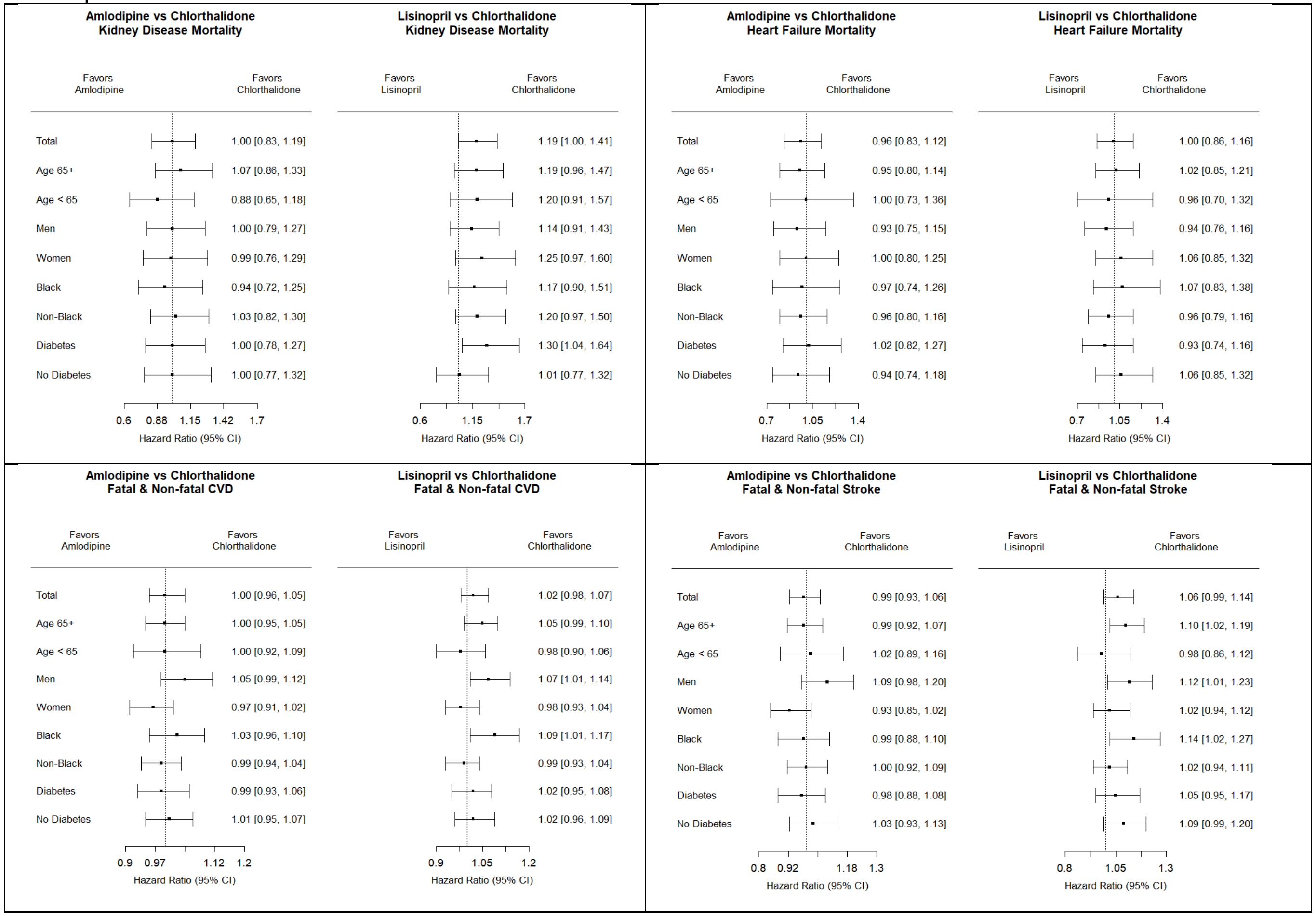

[a] Significant interactions were detected between Lisinopril/Chlorthalidone × female/male for fatal and non-fatal CVD (p=0.043), between Lisinopril/Chlorthalidone × Black/non-Black for fatal and nonfatal CVD (p=0.031), and between amlodipine/chlorthalidone × male/female for fatal and non-fatal stroke (p=0.027).

© 2023 Yamal JM et al. *JAMA Network Open.*

eFigure 2C. Unadjusted Hazard Ratios (95% CI) and Forest Plots of Overall and Subgroup Analyses of Mortality and Morbidity Outcomes Through the Extended Follow-up (by 12/31/2017) – Amlodipine vs. Chlorthalidone and Lisinopril vs. Chlorthalidone[a]

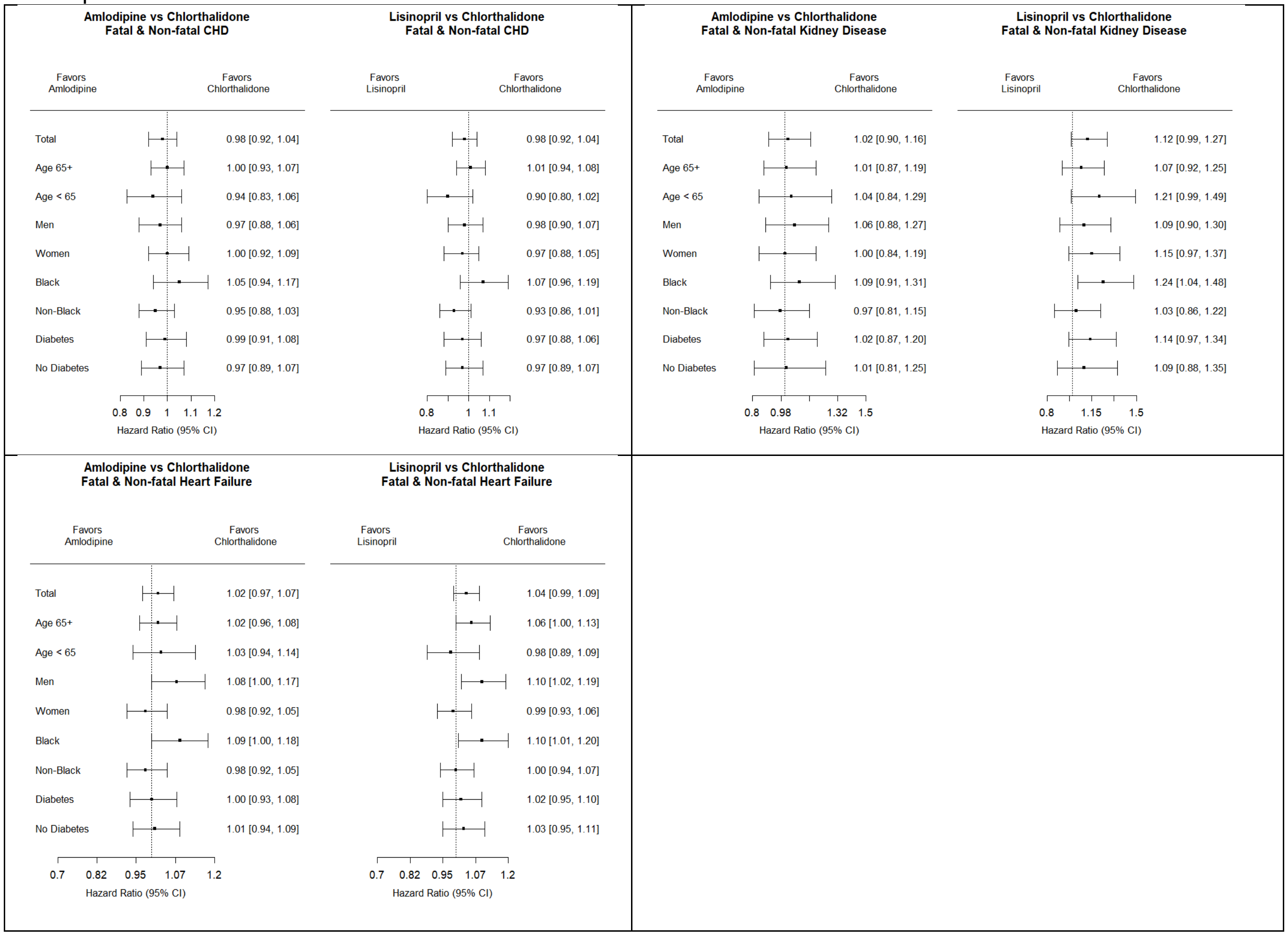

[a] Significant interactions were detected between Lisinopril/Chlorthalidone × Black/non-Black for fatal and non-fatal CHD (p=0.042) and between Lisinopril/Chlorthalidone × female/male for fatal and non-fatal heart failure (p=0.046).

© 2023 Yamal JM et al. *JAMA Network Open.*
