# Supplementary material for: Mortality and Morbidity Among Individuals With Hypertension Receiving a Diuretic, ACE Inhibitor, or Calcium Channel Blocker: A Secondary Analysis of a Randomized Clinical Trial
Source: JAMA Netw Open. 2023 Dec 4;6(12):e2344998. doi: 10.1001/jamanetworkopen.2023.44998 (PMC10696481; doi:10.1001/jamanetworkopen.2023.44998)
Supplement: Supplement 3. — Data Sharing Statement [file jamanetwopen-e2344998-s003.pdf]

Data Sharing Statement

Yamal. Mortality and Morbidity Among Individuals With Hypertension Receiving a Diuretic, ACE Inhibitor, or Calcium Channel Blocker. *JAMA Netw Open*. Published December 04, 2023. doi:10.1001/jamanetworkopen.2023.44998

Data
Data available: No

Additional Information
Explanation for why data not available: Limited dataset is available at https://biolincc.nhlbi.nih.gov/studies/allhat/
